# Supplementary material for: Loss of TET2 in human hematopoietic stem cells alters the development and function of neutrophils
Source: Cell Stem Cell. 2023 Jun 1;30(6):781–799.e9. doi: 10.1016/j.stem.2023.05.004 (PMC12356769; doi:10.1016/j.stem.2023.05.004)
Supplement: Document S1. Figures S1–S7 [file mmc1.pdf]

Cell Stem Cell, Volume *30*

Supplemental Information

Loss of TET2 in human hematopoietic stem cells alters the development and function of neutrophils

Hector Huerga Encabo, Iker Valle Aramburu, Manuel Garcia-Albornoz, Marion Piganeau, Henry Wood, Anna Song, Alessandra Ferrelli, Aneesh Sharma, Carlos M. Minutti, Marie-Charlotte Domart, Despoina Papazoglou, Kristian Gurashi, Miriam Llorian Sopena, Robert Goldstone, Todd Fallesen, Qian Wang, Linda Ariza-McNaughton, Daniel H. Wiseman, Kiran Batta, Rajeev Gupta, Venizelos Papayannopoulos, and Dominique Bonnet

Figure Supplementary 1

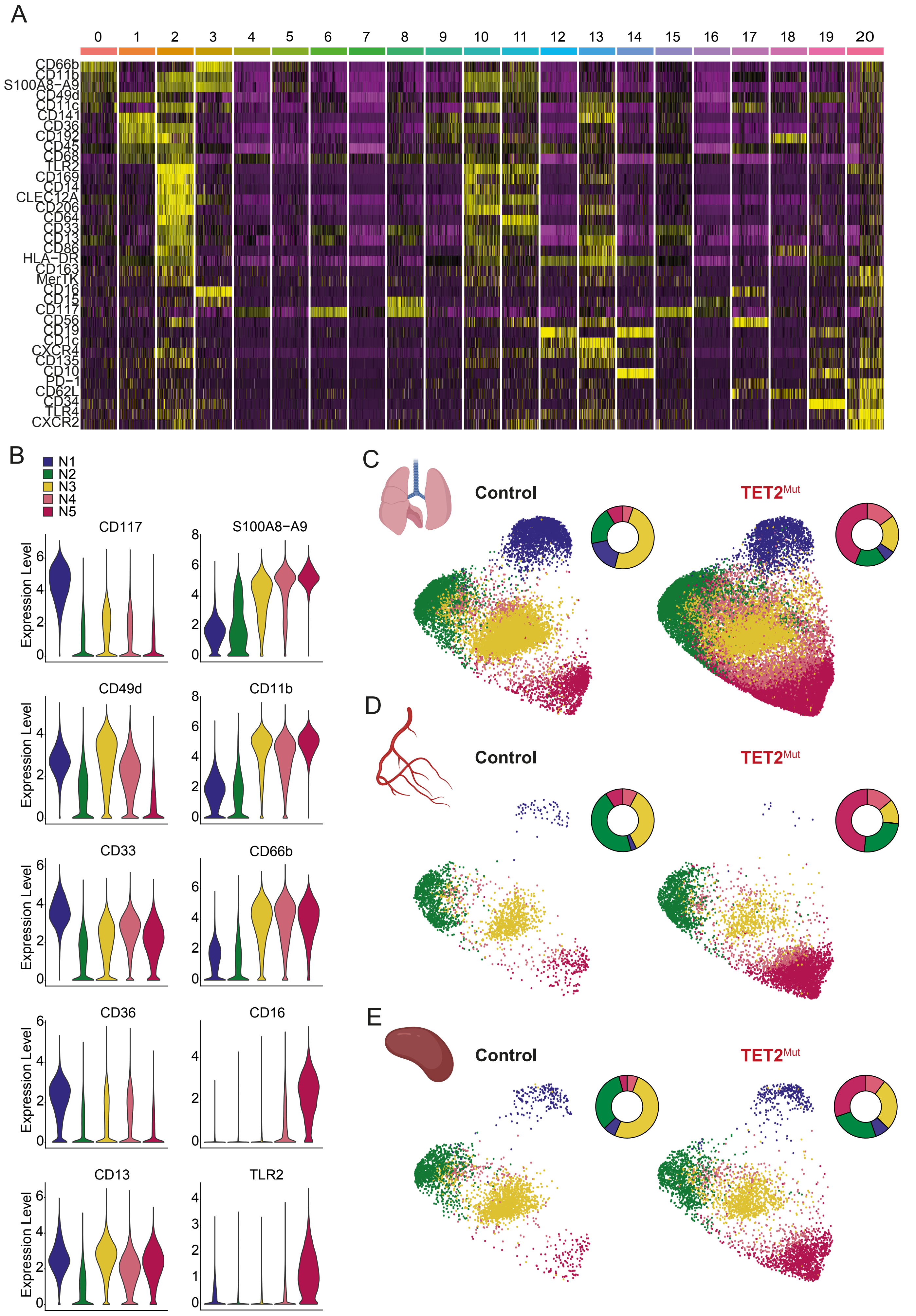

Figure S1. Mass cytometry analysis of neutrophil subsets in bone marrow and peripheral tissues of NSG mice engrafted with $TET2^{Mut}$ hHSCs. Related to Figure 1.

A. Heatmap showing the expression of the markers used in the CyTOF panel for each of the 21 clusters annotated in Fig. 1B-C.

B. Violin plots showing the expression of markers associated with different stages of the neutrophil differentiation across N1-N5 subsets.

C. Comparison of the neutrophil heterogeneity in the lung derived from control (14,983 cells) and $TET2^{Mut}$ (34,695 cells) hHSPCs by UMAP and pie-chart representing the percentage of each subset.

D. Comparison of the neutrophil heterogeneity in the blood derived from control (2283 cells) and $TET2^{Mut}$ (5,337 cells) hHSPCs by UMAP and pie-chart representing the percentage of each subset.

E. Comparison of the neutrophil heterogeneity in the spleen derived from control (3,093 cells) and $TET2^{Mut}$ (3,943 cells) hHSPCs by UMAP and pie-chart representing the percentage of each subset.

Figure Supplementary 2

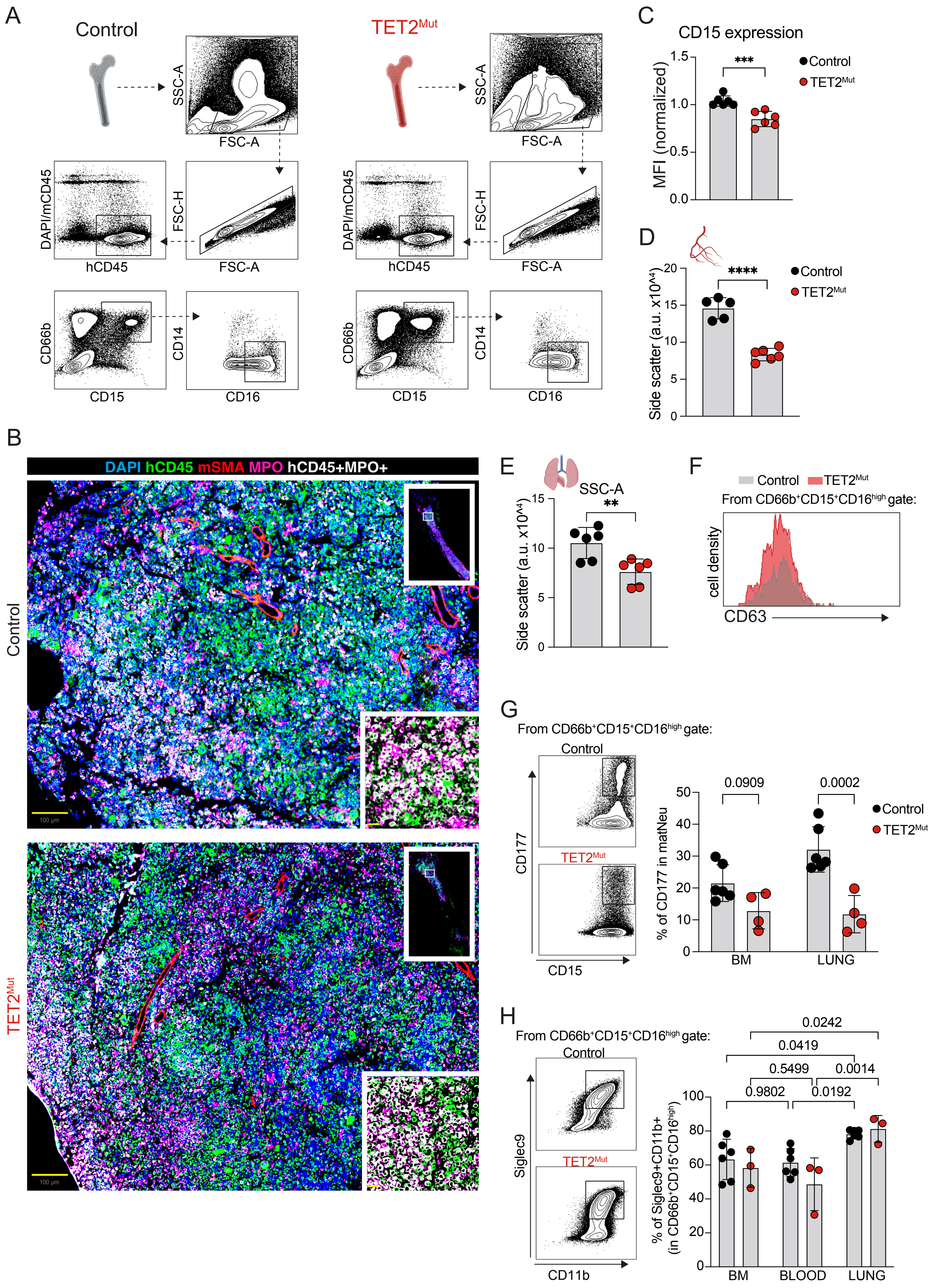

Figure S2. TET2^{Mut} hHSPCs exhibit exacerbated neutrophilia in the bone marrow and the lung. Related to Figure 2.

A. Gating strategy to identify human mature neutrophils in the bone marrow of mice reconstituted with control (black) or TET2^{Mut} (red) hHSPCs.

B. Representative large images from bone sections (top right) of mice engrafted with control or TET2^{Mut} hHSPCs. Enlarged areas (original magnification, 20X, scale bar: 100μm) to visualize the human hematopoietic system (green), vasculature (red) and MPO signal (pink). Amplified sections (bottom right, scale bar: 20μm) displaying human neutrophils co-stained with hCD45 and MPO (white signal).

C. Quantification of mean fluorescence intensity (MFI) of CD15 expression in CD66b$^+$CD15$^+$CD16^{high} neutrophils. (n = 6 mice, data from 3 independent experiments). For each experiment, CD15 MFI of one control neutrophil population was normalized to 1. Unpaired t test, *** $p<0.005$.

D-E. Quantification of the side scatter-area (SSC-A) of human CD66b$^+$CD15$^+$CD16^{high} neutrophils from the blood (D) and the lung (E) of humanized mice. (n = 5-6 mice, data representative of 3 independent experiments). Unpaired t test used for significance, ** $p<0.01$; **** $p<0.001$.

F. Representative histogram of CD63 expression in CD66b$^+$CD15$^+$CD16^{high} neutrophils. See Figure 2E for quantification.

G. Representative density plots of CD177$^+$ neutrophils within the CD66b$^+$CD15$^+$CD16^{high} cell population in the bone marrow (BM) and quantification of these cells in BM and lung of humanized mice reconstituted with control (black, n = 6) or TET2^{Mut} (red, n = 3) hHSPCs. Unpaired t test used for significance. p-values are displayed.

H. Representative density plots of Siglec9$^+$CD11b$^+$ neutrophils within the CD66b$^+$CD15$^+$CD16^{high} cell population in the bone marrow (BM) and quantification of these cells in the BM, blood and lung of humanized mice reconstituted with control (black, n = 6) or TET2^{Mut} (red, n = 3) hHSPCs. Unpaired t test used for significance. p-values are displayed.

Figure Supplementary 3

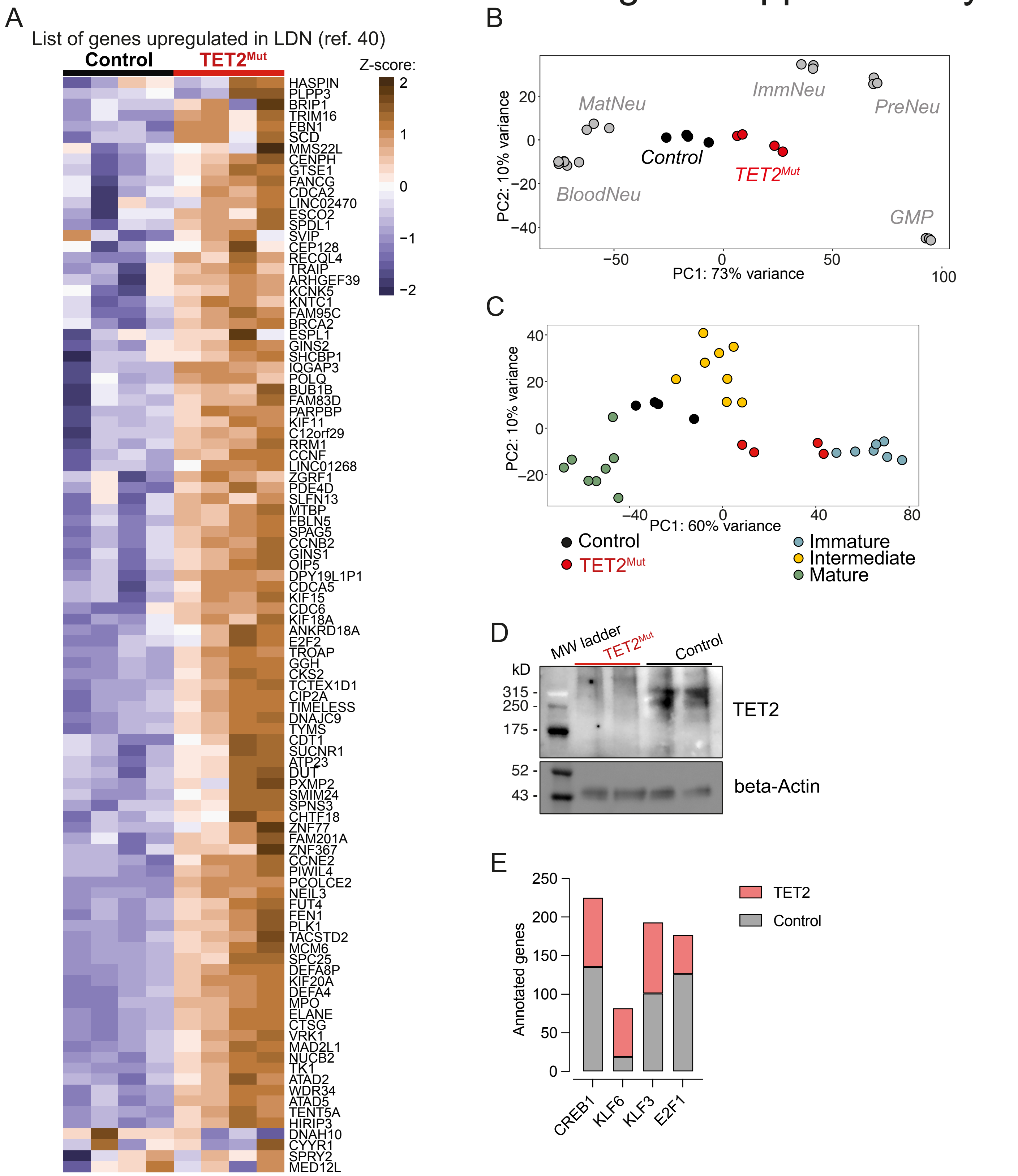

Figure S3. The transcriptome of TET2^{Mut} neutrophils is related to immature neutrophils. Related to Figure 3.

A. Heatmap showing the expression level of the gene set previously associated with LDN in neutrophils derived from control (black) or TET2^{Mut} (red) hHSPCs.

B. Principal component analysis comparing the transcriptional profile of our control (black) and TET2^{Mut} (red) neutrophils with the different bone marrow neutrophil subsets identified in the dataset GSE109467: GMP (granulocyte-myeloid progenitor), PreNeu (pre-neutrophils), ImmNeu (immature neutrophils), MatNeu (mature neutrophils) and BloodNeu (blood neutrophils). Analysis was done after filtering for ortholog genes between human and mouse.

C. Principal component analysis comparing the transcriptional profile of our control (black) and TET2^{Mut} (red) neutrophils with the 3 subsets (immature, intermediate and mature) of human neutrophils identified in the dataset GSE150021.

D. Western blot of TET2 performed in human CD66b+ cells isolated from the bone marrow of mice engrafted with control (black) or TET2^{Mut} (red) hHSPCs.

E. Transcription factor motif enrichment analysis from annotated peaks in control (grey) or TET2^{Mut} (red) neutrophil genome generated by gProfiler. The bars represent the number of annotated genes regulated by each transcription factor. See Table S3 for complete list of TF motifs and annotated genes.

Figure Supplementary 4

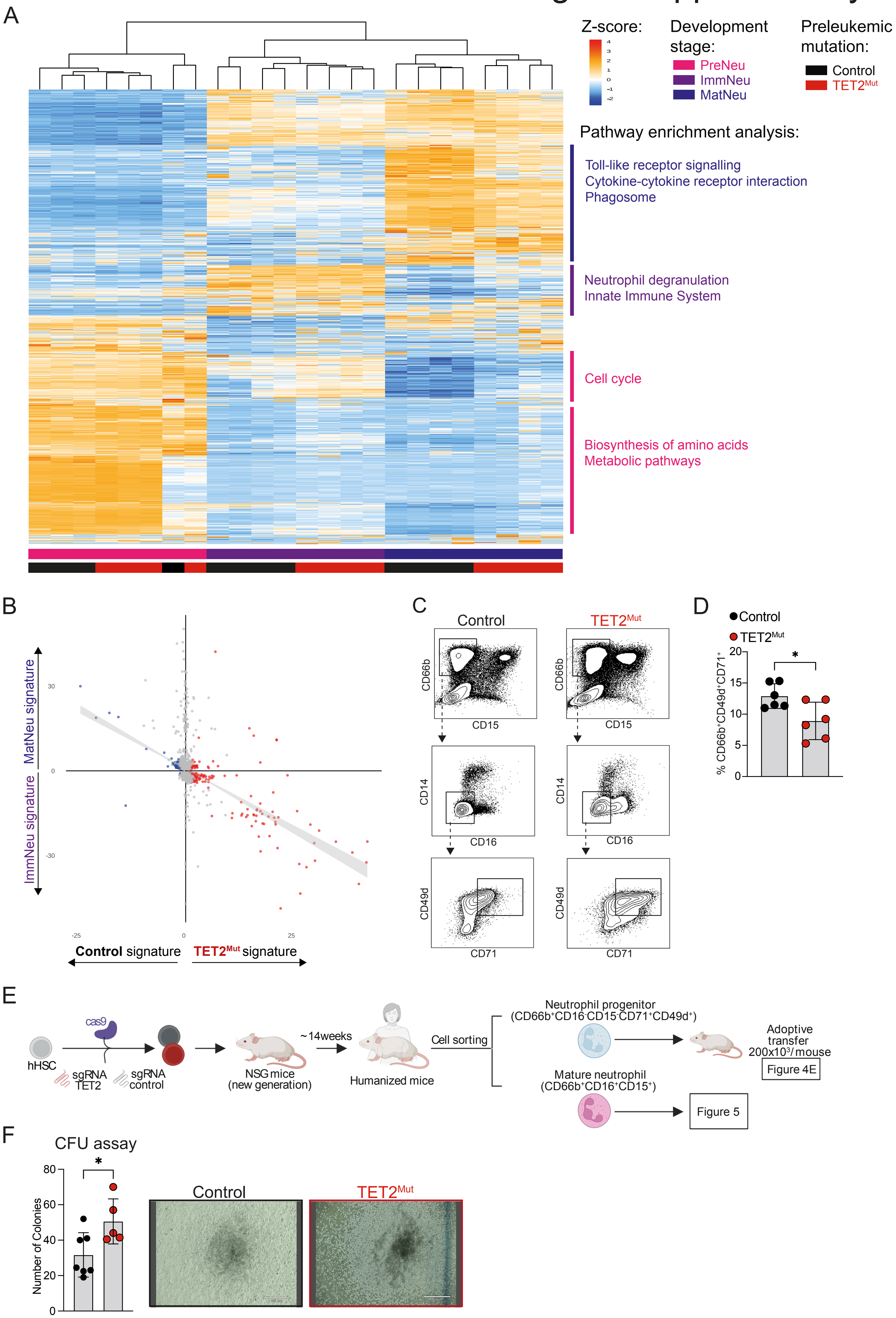

Figure S4. TET2 mutations impact on neutrophil precursors and transcriptome identity over different stages of the neutrophil development. Related to Figure 4.

A. Heatmap of the top 500 differentially expressed genes among the different cell populations. Enrichment pathway analysis was done by selecting characteristic gene sets for each cell state as indicated. See Table S4.

B. Scatterplots comparing the log2 fold changes of genes with adjusted p-values of <= 0.05 in the immNeu vs matNeu (y axis) comparison with the log2 fold changes of genes with adjusted p-values of <= 0.05 that appear downregulated (blue) or upregulated (red) in $TET2^{Mut}$ matNeu compared to control counterparts (x axis).

C. Representative gating strategy to identify and sort the neutrophil precursors (defined as $CD66b^{+}CD49d^{+}CD71^{+}CD14^{-}CD15^{-}CD16^{-}$) to perform the adoptive transfer.

D. Percentage of neutrophil precursors in the bone marrow of control or $TET2^{Mut}$ humanized mice. n = 6 mice, data representative of 3 independent experiments, each performed with hHSPCs from different human donors. Each dot represents one humanized mouse and error bars: mean ± SEM. Unpaired t test used for significance, * p<0.05.

E. Schematic representation of the generation humanized mice to study TET2-derived CH and the functional impact of TET2 mutations in human neutrophil progenitors and mature neutrophils. Created with BioRender.

F. Quantification of the colonies counted in the in vitro CFU assay. n = 5-7 mice, data from 2 independent experiments; each dot represents one biological donor and error bars: mean ± SEM. Unpaired t test used for significance, * p<0.05; Representative pictures of individual colonies are displayed at the right.

Figure Supplementary 5

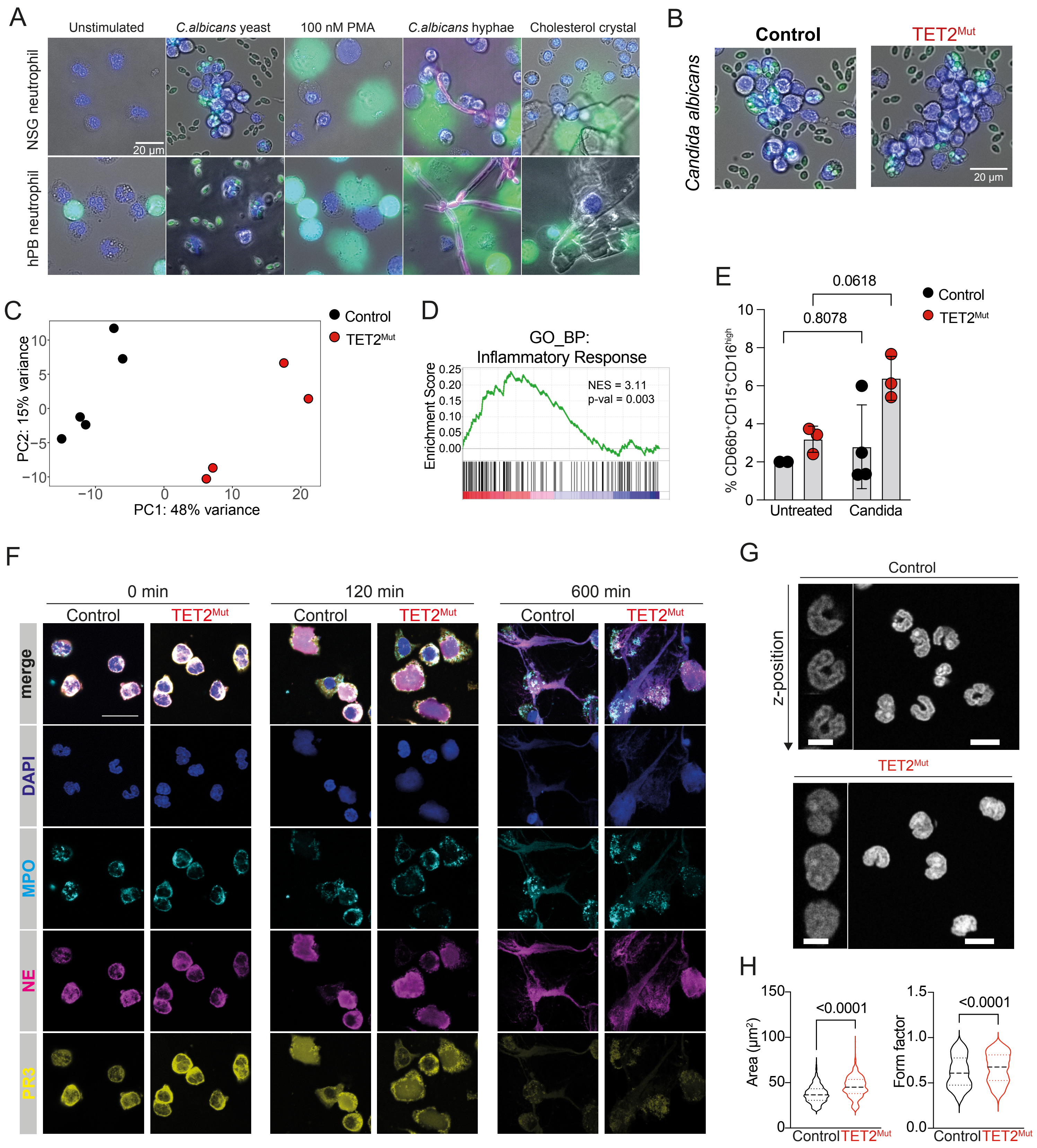

Figure S5. Exacerbated inflammatory response by TET2^{Mut} neutrophil upon LPS or *C.albicans* stimulation. Related to Figure 5.

A. Selection of snapshots of wide-field fluorescence microscopy images from timelapse imaging. Sorted human neutrophils from humanized mice (upper panel) and neutrophils isolated from peripheral human blood (lower panel) were stimulated with 100 nM PMA, yeast heat inactivated *C.albicans* (green), life *C.albicans* hyphae (magenta) and cholesterol crystals. Nuclei are stained with Hoechst and NETs are stained with sytox green. Scale bar 20 microns.

B. Representative fields of view used in the quantifications of phagocytosis capacity of control and TET2^{Mut} neutrophils of yeast heat inactivated *C.albicans*. Nuclei are stained with Hoechst and *C.albicans* are stained with sytox green. Scale bar 20 microns.

C. RNA-sequencing principal component analysis of control (black) and TET2^{Mut} (red) neutrophils after 3 hours of LPS stimulation (1μg/ml). n = 4 mice.

D. Gene Set Enrichment Analysis (GSEA) showing upregulation of inflammatory response pathway using the Gene Ontology biological process (GO_BP) database. See Table S5 for complete list of pathways.

E. Quantification by flow cytometry of human neutrophil percentage in the lung of mice engrafted with control (black) or TET2^{Mut} (red) hHSPCs in homeostasis (untreated) or 24 hours post *C.albicans* challenge. One-way ANOVA test used for significance; p-values are displayed in the figure.

F. Representative confocal microscopy images of sorted human control and TET2^{Mut} neutrophils stimulated with 100 nM PMA and fixed at timepoints 0, 120 and 600 minutes. Cells were stained with antibodies against MPO (cyan), NE (magenta) and PR3 (yellow). Nuclei are stained with DAPI. Scale bar 20 microns.

G-H. Representative confocal microscopy image (G) and quantification (H) of nuclear shape from sorted human control and TET2^{Mut} neutrophils stained with DAPI shown as a z-montage of a selected nucleus (scalebar 5 microns) or a selection of nuclei (scalebar 10 microns). H. Violin plot of quantified area or form factor per cell of neutrophils obtained from 3 different mice per group. Unpaired t test was used for significance.

Figure Supplementary 6

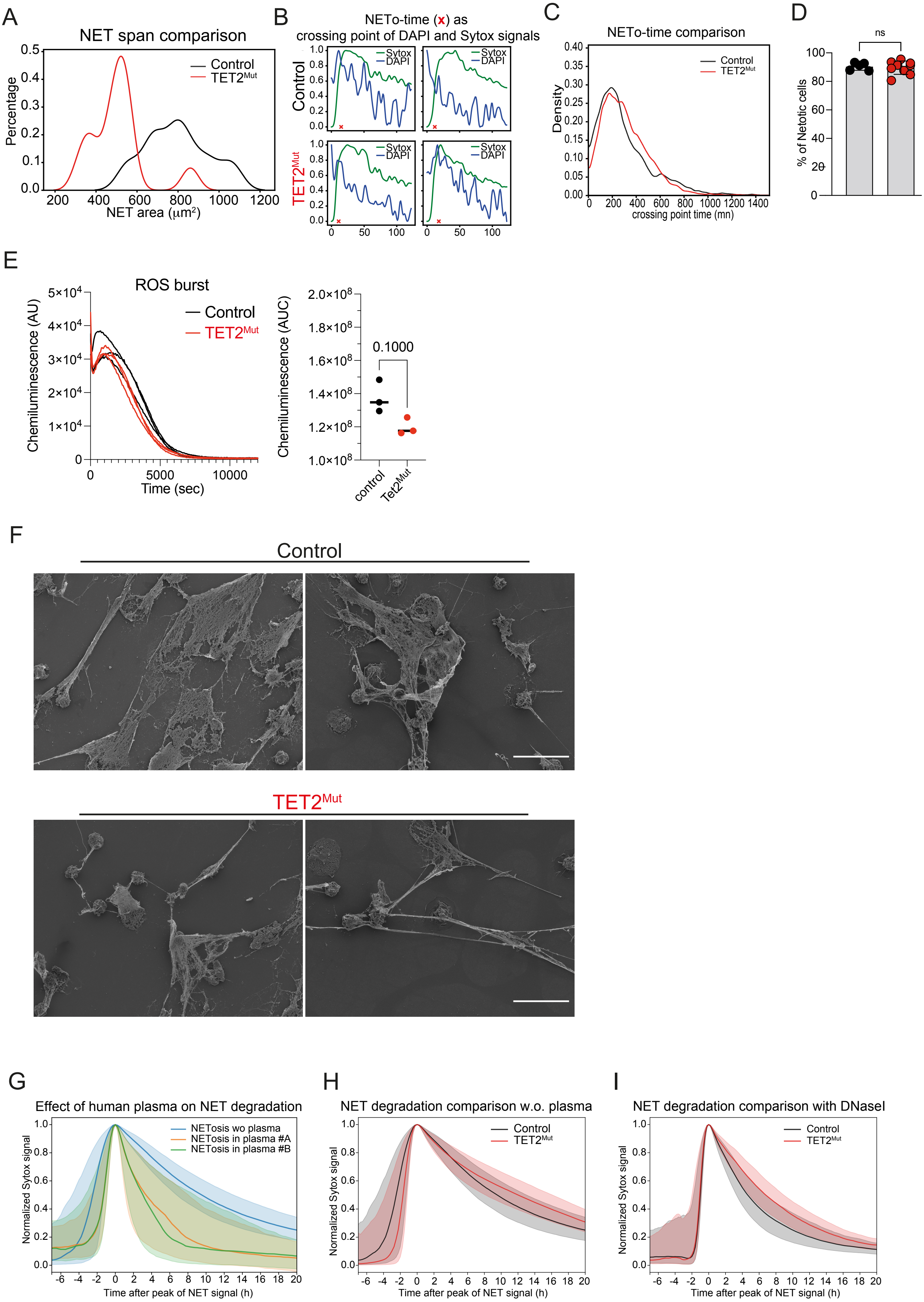

Figure S6. Analysis of NET formation and clearance in TET2^{Mut} neutrophils. Related to Figure 6.

A. Distribution of NET spans for control and TET2^{Mut} neutrophils. The NET span was estimated for each video as the contribution of an individual netotic cell to the total NET coverage based on the pixROI classification. The total NET coverage corresponds to the maximal spatial span ever reached by all possible NETs formed over time.

B. Examples of crossing time points between Sytox and DAPI signal used to estimate times of NETosis. NETosis is assumed to happen when the normalized DAPI signal crosses the normalized Sytox signal.

C. Distributions of NETo-times (times of NETosis) for control and TET2^{Mut} neutrophils. The distribution is computed after gathering 1000 random netotic crossing points for all videos corresponding to the mouse type and experimental condition.

D. Percentage of control and TET2^{Mut} neutrophils undergoing NETosis upon 100nM PMA. Each dot represents one biological replicate. Data representative from 2 independent experiments. Control neutrophil (black dots) n = 5; TET2^{Mut} neutrophils (red dots) n = 9.

E. ROS production by control and TET2^{Mut} neutrophils upon PMA stimulation, detected by chemiluminescence of luminol over time (left panel). Area under the curve (AUC) of each ROS burst (right panel). n=3 per group, representative of 2 independent experiments.

F. Scanning electron microscopy images of NETs formed upon 100 nM PMA stimulation from control or TET2^{Mut} neutrophils sorted from the bone marrow of humanized mice. 1000x magnification was used with dwell times of 10 microseconds. Scale-bar 30 microns.

G. NET degradation traces from control neutrophils in the absence and presence of human plasma. Median curves (solid lines) in the absence of plasma (blue line) or in the presence of 2 different plasmas (orange and green lines) of netotic pixel signals aligned and normalized to their peak as classified by the pixROI method. Shaded regions are bounded by the Q1 and Q3 curves corresponding to the 25% and 75% quartiles. The curves are computed on signals gathering 1000 randomly sampled pixels for all videos corresponding to control neutrophils.

H. NET degradation profiles of control and TET2^{Mut} neutrophils in the absence of plasma. Median curves (solid lines) of netotic pixel signals aligned and normalized to their peak as classified by the pixROI method. Shaded regions are bounded by the Q1 and Q3 curves corresponding to the 25% and 75% quartiles. The half-life was 585 min (Q1 = 420 min; Q3 = 780 min) for control NETs and 720 min (Q1 = 495 min; Q3 = 975 min) for TET2^{Mut} NETs. The curves are computed on signals gathering 1000 randomly sampled pixels for all videos corresponding to the mouse type and experimental condition.

I. NET degradation profiles of control and TET2^{Mut} neutrophils when adding DNAse. Median curves (solid lines) of netotic pixel signals aligned and normalized to their peak as classified by the pixROI method. Shaded regions are bounded by the Q1 and Q3 curves corresponding to the 25% and 75% quartiles. The half-life of control NETs was 288 min (Q1 = 204 min; Q3 = 408 min) and the half-life of TET2^{Mut} NETs was 408 min (Q1 = 300 min; Q3 = 540 min). The curves are computed on signals gathering 1000 randomly sampled pixels for all videos corresponding to the mouse type and experimental condition.

Figure Supplementary 7

A

Humanized mice bone marrow analysis:

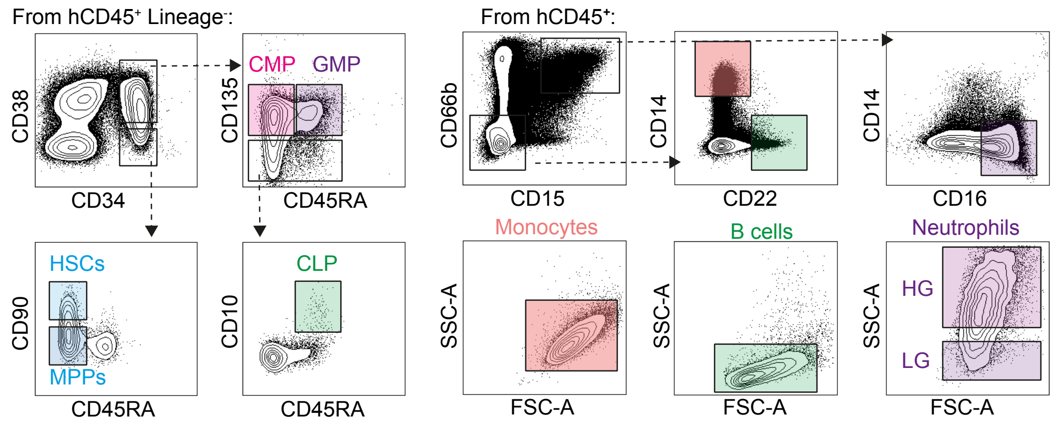

B

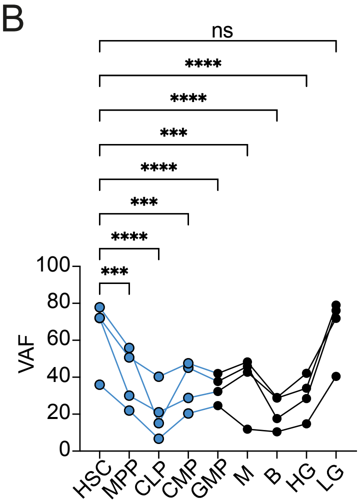

C

UCLH cohort analysis:

From bone marrow aspirates: mature Neutrophils defined as $CD45^+CD34^-CD33^+CD10^+Lin^-$ and myeloid compartment defined as CD45+CD34-CD3-CD19-CD5-CD14-CD20-CD56-

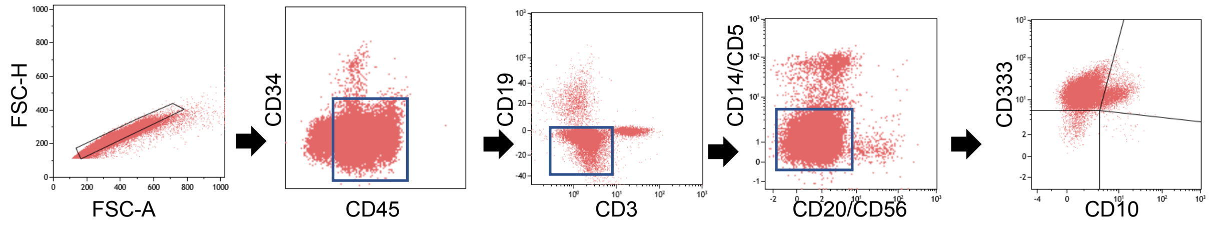

D

Manchester cohort analysis:

From Neutrophils isolated from peripheral blood samples: immmature Neutrophils defined as $CD66b^+CD16^-$

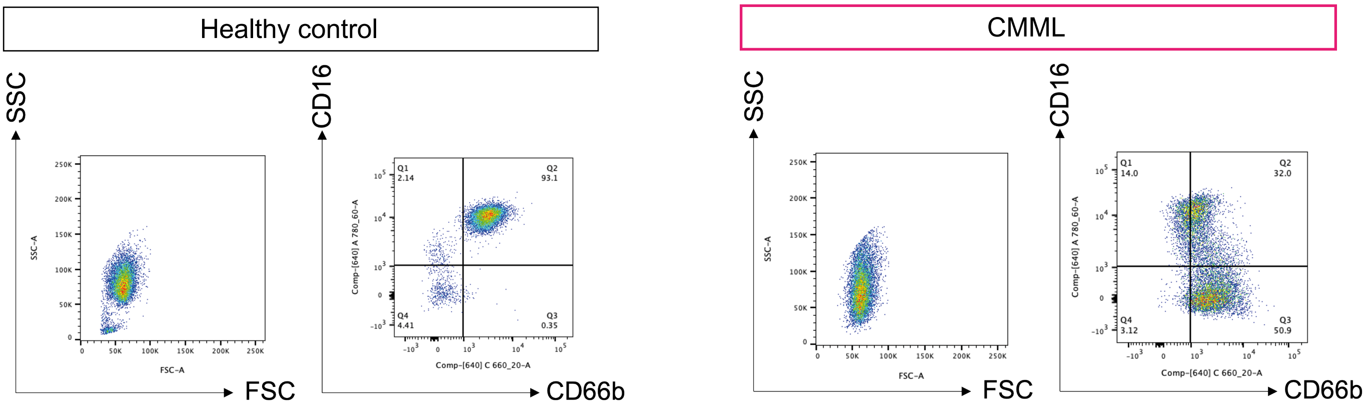

Figure S7. Isolation and identification of the different immune cells in TET2-derived human clonal hematopoiesis. Related to Figure 7.

A. Representative sorting strategy to analyze the TET2 VAF in different stem and progenitor cells and in differentiated hematopoietic cells.

B. Sequencing raw data from the different cell populations analyzed in the bone marrow to show that VAF of HSC is significantly higher compared to all populations except to LG neutrophils. Two-way ANOVA test, * $p<0.05$; ** $p<0.01$; *** $p<0.005$; **** $p<0.001$.

C. Representative flow gating strategy to analyze mature neutrophils defined as $CD45^{+}CD34^{-}CD3^{-}CD19^{-}CD5^{-}CD14^{-}CD20^{-}CD56^{-}CD33^{+}CD10^{+}$ in the bone marrow aspirate of the 22 UCLH cohort patients.

D. Representative flow gating strategy after the neutrophil isolation from peripheral blood samples of the 24 Manchester cohort patients.
