# Supplementary material for: Loss of TET2 in human hematopoietic stem cells alters the development and function of neutrophils
Source: Cell Stem Cell. 2023 Jun 1;30(6):781–799.e9. doi: 10.1016/j.stem.2023.05.004 (PMC12356769; doi:10.1016/j.stem.2023.05.004)
Supplement: Document S2. Article plus supplemental information [file mmc9.pdf]

Cell Stem Cell

Article

Loss of TET2 in human hematopoietic stem cells alters the development and function of neutrophils

Graphical abstract

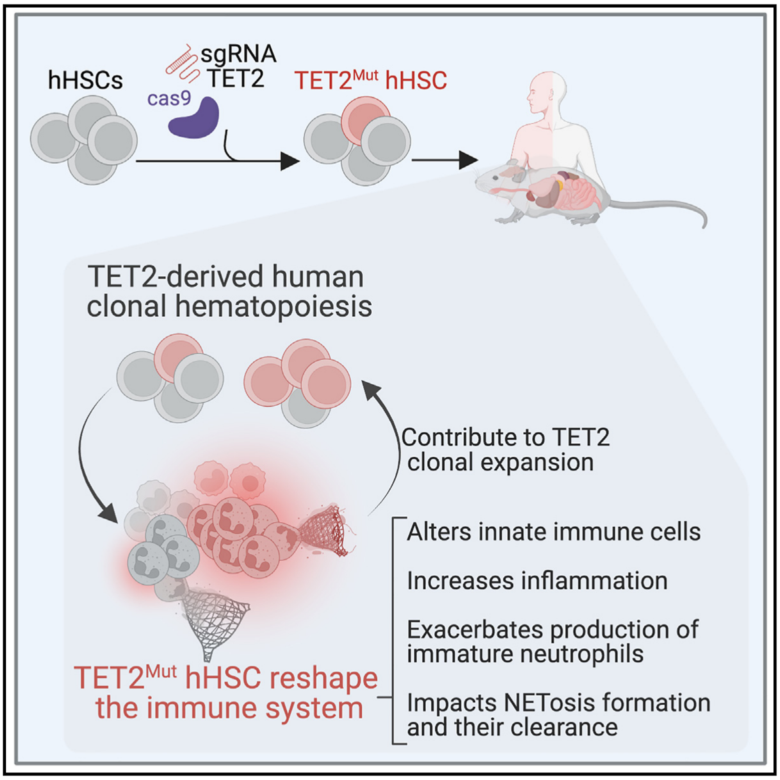

Highlights

- TET2 mutations in human HSCs shape the innate immune system development in NSG mice
- $TET2^{Mut}$ human HSCs show an exacerbated production of aberrant neutrophils
- $TET2^{Mut}$ neutrophils show defects in antimicrobial strategies and NET clearance
- TET2 mutations in humans correlate with the presence of low-granule neutrophils

Authors

Hector Huerga Encabo,
Iker Valle Aramburu,
Manuel Garcia-Albornoz, ...,
Rajeev Gupta,
Venizelos Papayannopoulos,
Dominique Bonnet

Correspondence

hector.encabo@crick.ac.uk (H.H.E.),
veni.p@crick.ac.uk (V.P.),
dominique.bonnet@crick.ac.uk (D.B.)

In brief

Huerga Encabo et al. describe how TET2 mutations affect the human innate immune system in immunodeficient mice. They focus on human neutrophil development using $TET2^{Mut}$ hHSCs to describe immature transcriptomic and epigenomic signatures and impaired immune effector functions that link to pathologies associated with clonal hematopoiesis.

Check for updates

Huerga Encabo et al., 2023, Cell Stem Cell *30*, 781–799
June 1, 2023 © 2023 The Authors. Published by Elsevier Inc.
https://doi.org/10.1016/j.stem.2023.05.004

CellPress

Cell Stem Cell

CellPress OPEN ACCESS

Article

Loss of TET2 in human hematopoietic stem cells alters the development and function of neutrophils

Hector Huerga Encabo,[1,9,*] Iker Valle Aramburu,[2,9] Manuel Garcia-Albornoz,[1] Marion Piganeau,[1] Henry Wood,[1] Anna Song,[1] Alessandra Ferrelli,[1] Aneesh Sharma,[1] Carlos M. Minutti,[3] Marie-Charlotte Domart,[4] Despoina Papazoglou,[1] Kristian Gurashi,[8] Miriam Llorian Sopena,[5] Robert Goldstone,[5] Todd Fallesen,[6] Qian Wang,[2] Linda Ariza-McNaughton,[1] Daniel H. Wiseman,[8] Kiran Batta,[8] Rajeev Gupta,[7] Venizelos Papayannopoulos,[2,*] and Dominique Bonnet[1,10,*]

[1]Haematopoietic Stem Cell Laboratory, The Francis Crick Institute, 1 Midland Road, London NW1 1AT, UK
[2]Laboratory of Antimicrobial Defence, The Francis Crick Institute, 1 Midland Road, London NW1 1AT, UK
[3]Immunobiology Laboratory, The Francis Crick Institute, 1 Midland Road, London NW1 1AT, UK
[4]Electron Microscopy Science Technology Platform, The Francis Crick Institute, 1 Midland Road, London NW1 1AT, UK
[5]Bioinformatics and Biostatistics, The Francis Crick Institute, 1 Midland Road, London NW1 1AT, UK
[6]Advanced Light Microscopy Science Technology Platform, The Francis Crick Institute, 1 Midland Road, London NW1 1AT, UK
[7]Haematology Stem Cell Group, UCL Cancer Institute, London, UK
[8]Division of Cancer Sciences, The University of Manchester, Manchester, UK
[9]These authors contributed equally
[10]Lead contact
*Correspondence: hector.encabo@crick.ac.uk (H.H.E.), veni.p@crick.ac.uk (V.P.), dominique.bonnet@crick.ac.uk (D.B.)
https://doi.org/10.1016/j.stem.2023.05.004

SUMMARY

Somatic mutations commonly occur in hematopoietic stem cells (HSCs). Some mutant clones outgrow through clonal hematopoiesis (CH) and produce mutated immune progenies shaping host immunity. Individuals with CH are asymptomatic but have an increased risk of developing leukemia, cardiovascular and pulmonary inflammatory diseases, and severe infections. Using genetic engineering of human HSCs (hHSCs) and transplantation in immunodeficient mice, we describe how a commonly mutated gene in CH, TET2, affects human neutrophil development and function. TET2 loss in hHSCs produce a distinct neutrophil heterogeneity in bone marrow and peripheral tissues by increasing the repopulating capacity of neutrophil progenitors and giving rise to low-granule neutrophils. Human neutrophils that inherited TET2 mutations mount exacerbated inflammatory responses and have more condensed chromatin, which correlates with compact neutrophil extracellular trap (NET) production. We expose here physiological abnormalities that may inform future strategies to detect TET2-CH and prevent NET-mediated pathologies associated with CH.

INTRODUCTION

Neutrophils are constantly generated by hematopoietic stem and progenitor cells (HSPCs) from the bone marrow and exhibit a rapid turnover under homeostasis that is estimated to be 10^9 cells/kg per day.[1] Recent studies using single-cell transcriptomics (scRNA-seq) and cytometry by time of flight (CyTOF) have characterized the cell heterogeneity within the myeloid compartment in the bone marrow and defined a precise stepwise model of differentiation from HSPCs, which includes common myeloid progenitor cells (CMPs), granulocyte monocyte progenitor cells (GMPs), proliferating neutrophil progenitors (preN), non-proliferating differentiated immature neutrophil (immNeu), and mature neutrophils.[2–5] Therefore, somatic mutations that naturally occur in HSPCs during our lifespan will be transmitted to functional neutrophils shaping the immune response of the host. Accumulation of mutations in HSPCs resulting in the outgrowth of mutant clones is a natural process referred to as clonal hematopoiesis (CH). Individuals with CH have a higher risk of developing hematological malignancies such as acute myeloid leukemia (AML) or myelodysplastic syndrome (MDS); thus, mutations implicated in CH are also known as preleukemic mutations.[6–8]

The two most affected genes in CH are DNMT3A and TET2, two epigenetic factors involved in DNA methylation and demethylation, respectively.[9,10] Preleukemic mutations in HSC have been described to confer a repopulating advantage upon stress-induced hematopoiesis in mouse models, and recent works have aided our understanding of how preleukemic clones expand upon inflammatory environments.[11–13] CH has also been associated with a higher risk of developing cardiovascular disease, chronic obstructive pulmonary disease (COPD), and severe symptoms upon certain microbial infections.[14–17] The connection between CH and non-hematologic disorders has mainly been approached from the perspective of the impact of mutations in the monocytic compartment. In this regard, it has been described that monocytes and macrophages from TET2-knockout (KO) mice produce higher levels of interleukin IL-1β and IL-6 leading to an exacerbated inflammatory response.[18–21]

This is an open access article under the CC BY license (http://creativecommons.org/licenses/by/4.0/).

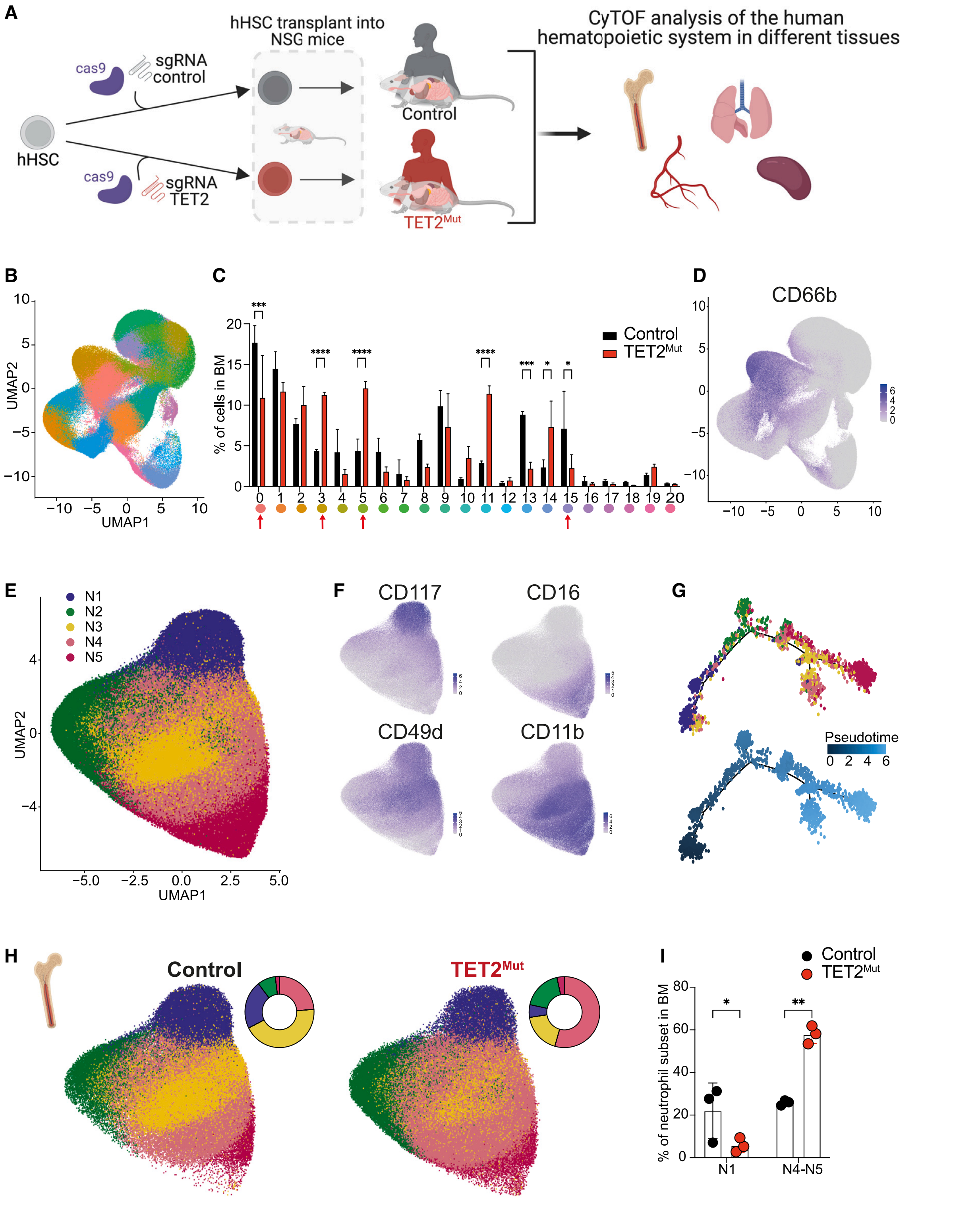

Figure 1. Identification of human neutrophil heterogeneity derived from TET2^{Mut} hHSPCs in bone marrow and peripheral tissues of humanized mice

(A) Schematic representation of the design to study the impact of TET2 loss in hHSPCs in the reconstitution of the human immune system in different tissues of NSG mice. Created with BioRender.

(B) Uniform manifold approximation and projection (UMAP) dimensionality reduction of human hematopoietic cells isolated from bone marrow of mice engrafted with wild-type human HSCs (hHSPCs). 1.3 million cells are represented into 21 clusters.

(legend continued on next page)

Defects in neutrophil antimicrobial functions such as degranulation, phagocytosis, and specially the release of neutrophil extracellular traps (NETs) have been linked to the pathologies associated with CH patients. NETs are extracellular web-like structures composed of decondensed chromatin and antimicrobial proteins, and dysregulated NET formation or defects in NET clearance is linked to the development of atherosclerosis, rheumatoid arthritis, thrombosis, cystic fibrosis, and COPD.[22–30] Therefore, preleukemic mutations acquired by HSC might modify the function of neutrophil progeny and contribute to the development of hematological diseases. As an example, in a Jak2 mutant mouse model, neutrophils showed an increase in NET production contributing to the thrombosis associated with myeloproliferative neoplasms.[31] Nevertheless, the potential role that preleukemic human neutrophils can play in the pathologies associated with CH individuals remains mainly unexplored.[32]

In this work, we uncover that human HSPCs (hHSPCs) carrying TET2 mutations generate neutrophils that show characteristics of immature stages. We also show that the repopulating advantage that TET2 mutations are known to confer is not only restricted to HSPCs but also occurs in committed progenitors such as neutrophil progenitors. Finally, we define the functional properties of the TET2^{Mut} neutrophils, which are characterized by reduced phagocytic capacity and by production of compact NETs that are more resilient to degradation. Our results provide new cellular and molecular evidence to understand why individuals with TET2-derived CH have an increased risk to develop neutrophil-associated pathologies such as severe microbial infections, cardiovascular diseases, and COPD.[33]

RESULTS

TET2 mutations in human hematopoietic stem/progenitor cells alter neutrophil dynamics in the bone marrow and peripheral tissues

Using CRISPR-Cas9 editing, we introduced TET2 loss-of-function mutations in hHSPCs (Lineage^{-}CD34^{+}CD38^{-}). These cells were transplanted into non-conditioned NSG-SGM3-ckit$^{W41/W41}$ mice.[34,35] After 12–14 weeks to allow the human reconstitution, we used CyTOF mass cytometry to define and compare the different human immune cell types present in the bone marrow and different tissues of mice engrafted with wild-type (control) or TET2 mutant hHSPCs (TET2^{Mut}) (Figure 1A). We identified 21 different cell subsets with our antibody panel (Table S1) (Figures 1B and S1A). In the bone marrow, 7 cell subsets appear differentially represented (Figure1C) between control and TET2^{Mut}, and of those, 4 cell subsets (0, 3, 5, and 15) were related to granulopoiesis (Figure 1D). To focus on granulocyte heterogeneity, we selected these 4 subsets for further clustering analysis, revealing 5 main subsets (N1–N5) (Figure 1E). Cells in subset N1 express CD117 and CD49d, markers previously annotating neutrophil progenitors, and show a low expression of CD11b, CD16, and S100A8-9, markers associated with mature neutrophils (Figures 1F and S1B). Expression of markers associated with differentiation increase progressively across subsets N1–N5, defining a clear trajectory of neutrophil development (Figures 1G and S1B). Strikingly, the neutrophil heterogeneity found in humanized mice resembles the previous heterogeneity reported in human bone marrow.[2] We also characterized the human neutrophil heterogeneity in the blood, lung, and spleen of these mice, something that has not been explored previously. Interestingly, although N1 subset was rare in the blood and the spleen, the lung contained a similar N1 proportion to the one found in the BM (Figures 1H and S1C–S1E), suggesting that in humanized mice, the lung might act as an extramedullary organ for neutrophil supply. Thus, humanized mice serve as a powerful tool to study the impact of certain mutations in human hematopoietic stem cells and how these mutations could affect neutrophil development in the BM and the periphery.

Focusing on granulopoiesis from TET2^{Mut} hHSPCs, we observed an imbalance in the neutrophil heterogeneity, toward increased differentiated subsets and decreased neutrophil progenitor in the BM (Figures 1H and 1I). Similar results show that exacerbated neutropoiesis from TET2^{Mut} HSPCs were obtained in the lung, blood, and spleen (Figures S1C–S1E). Overall, our CyTOF analysis reveals that TET2 mutations in hHSPCs shape the immune system and alter neutrophil heterogeneity within the bone marrow and peripheral tissues of humanized mice.

TET2^{Mut} hHSPCs undergo exacerbated neutropoeisis composed of low-granule neutrophils

To validate these observations and consistent with the CyTOF data, we also observed by flow cytometry that the bone marrow of mice engrafted with TET2^{Mut} hHSPCs is characterized by an increased proportion of neutrophils defined as CD66b^{+}CD15^{+}CD16^{hi} (Figures 2A and S2A). We also confirmed that the human hematopoietic cells and, in particular, the neutrophils were homogenously engrafted in the bone marrow of mice transplanted with control or TET2^{Mut} hHSPCs (Figure S2B). When analyzing specific traits of preleukemic neutrophils (TET2^{Mut}CD66b^{+}CD15^{+}CD16^{hi}) by flow cytometry, we observed a significant reduction of CD15 surface expression and, interestingly, a lower side-scatter

(C) Frequencies of the 21 clusters identified in the bone marrow of mice reconstituted with wild-type (black bars) or TET2 mutant (red bars) hHSPC (data showing mean and SD from 3 biological replicates). Two-way ANOVA test used for significance, *$p < 0.05$; **$p < 0.01$; ***$p < 0.005$; ****$p < 0.001$.

(D) Single-cell expression of CD66b used to define clusters containing granulocytes. See Figure S1A. Clusters 0, 3, 5, and 15 were selected for downstream analysis of neutrophil heterogeneity.

(E) UMAP dimensionality reduction of 365 thousand cells to define the 5 neutrophil subsets (labeled as N1–N5).

(F) Single-cell expression of progenitor markers CD117 and CD49d and maturation markers CD16 and CD11b.

(G) Pseudo-time trajectory to determine the neutrophil development across N1–N5 subsets. A sub-setting of 500 cells per cluster was obtained to perform the pseudo-time analysis.

(H) Comparison of the neutrophil heterogeneity in the bone marrow derived from control (148,464 cells) and TET2 mutant (152,708) hHSPCs by UMAP and pie-chart representing the percentage of each subset.

(I) Frequencies of N1–N3 neutrophils and N4–N5 neutrophils in the bone marrow of mice reconstituted with wild-type (black dots) or TET2 mutant (red dots) hHSPC (each dot represents one biological replicate). n = 3 mice. Two-way ANOVA test used for significance, *$p < 0.05$; **$p < 0.01$; ***$p < 0.005$; ****$p < 0.001$.

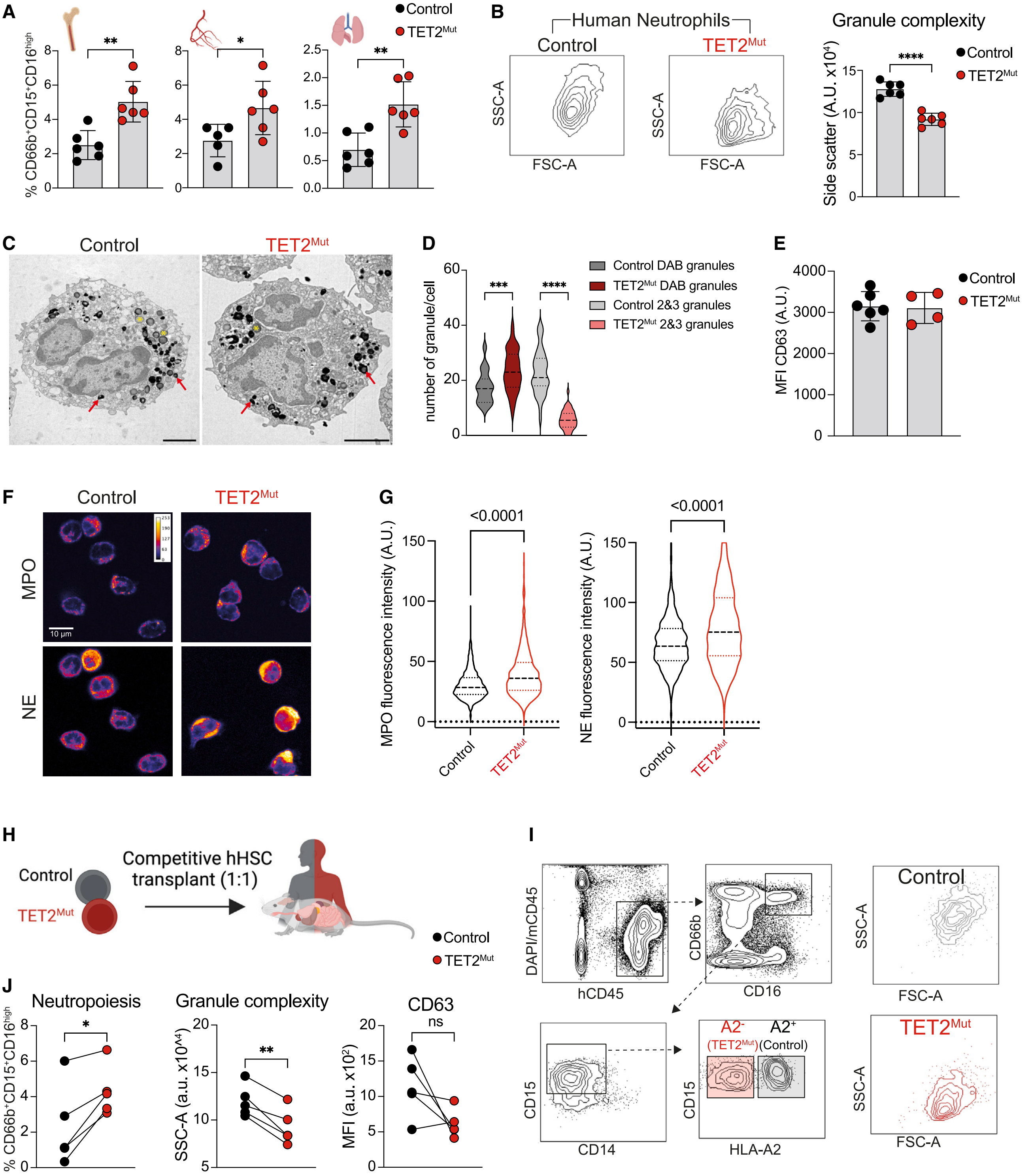

Figure 2. Neutrophils derived from TET2^{Mut} hHSPCs have reduced granule complexity

(A) Percentage of CD66b$^+$CD15$^+$CD16^{high} cells within human hematopoietic system in bone marrow, blood, and lung of mice engrafted with control (black) or TET2^{Mut} (red) hHSPCs (n = 6 mice, data representative of 3 independent experiments, each performed with hHSPCs from different human donors). Each dot represents one humanized mouse and error bars: mean ± SEM. Unpaired t test used for significance, *$p < 0.05$; **$p < 0.01$.

(B) Representative side-scatter plots of mature neutrophils and quantification of side-scatter area (a.u., arbitrary units acquired from the analyzer). n = 6 mice, data representative of 3 independent experiments, each performed with hHSPCs from different human donors. Each dot represents one humanized mouse and error bars: mean ± SEM. Unpaired t test used for significance, *$p < 0.05$; **$p < 0.01$.

(legend continued on next page)

(SSC) pattern (Figures 2B and 2C, respectively), which reflects that neutrophils derived from TET2^{Mut} HSPCs have low granule (LG) complexity. Also, in peripheral blood and lungs of mice engrafted with TET2^{Mut} hHSPCs, we observed an increased proportion of neutrophils characterized by LG complexity (Figures 2A, S2D, and S2E). Intrigued by the clear shift in the side-scatter profile by flow cytometry, we sought to determine the granule complexity of TET2^{Mut} neutrophils by transmission electron microscopy (TEM). Interestingly, quantification of primary granules that positively stained with diaminobenzidine (DAB)[36] and secondary and tertiary granules revealed that TET2^{Mut} neutrophils show an increased number of primary granules, but the presence of secondary and tertiary granules was significantly reduced (Figures 2C and 2D). This particular granule content was accompanied by an absence of differences in markers associated with degranulation or activation of neutrophils, such as CD63 or CD11b, Siglec9, and CD177[37–39] (Figures 2E and S2F–S2H). The similar levels in these markers suggest that LG complexity in TET2^{Mut} neutrophils could not be explained by an elevated activation status that would lead to loss of granular content. Quantification of primary granule proteins such as myeloperoxidase (MPO) and neutrophil elastase (NE) further support the significant increase of primary granules in TET2^{Mut} neutrophils (Figures 2F and 2G). Of note, we have established that these observations are cell intrinsic as in mice engrafted with a mix of TET2^{WT} control and TET2^{Mut} hHSPCs, TET2^{Mut} neutrophil abundance is increased compared with control counterparts from the same host, and TET2^{Mut} neutrophils were also characterized by LG complexity with no increase in degranulation marker CD63 (Figures 2H–2J). Overall, our data indicate that TET2 mutations in hHSPCs cause an exacerbated clonal production of neutrophils with a higher content of primary granules but a lower content of secondary and tertiary granules.

TET2^{Mut} hHSPCs produce neutrophils with a transcriptional program characteristic of immature stages and low-density neutrophils (LDN)

We then performed bulk RNA-seq on CD66b^{+}CD15^{+}CD16^{hi} sorted control and TET2^{Mut} neutrophils. Principle component analysis of control and TET2^{Mut} neutrophils clustered the samples separately indicating that they have a distinct transcriptional signature (Figure 3A; Table S2). Of note, the most differentially expressed genes in TET2^{Mut} neutrophils were genes associated with neutrophil primary granule compositions, such as MPO, ELANE, CTSG, or PRNT3 (Figure 3B). Intriguingly, the transcriptome of TET2^{Mut} neutrophils also resembles the one described in low-density neutrophils (LDNs), a heterogeneous cell population that appears in the context of stress-induced hematopoiesis.[40] We observed that gene sets that have been recently reported to be highly expressed in LDN are upregulated in TET2^{Mut} neutrophils (Figure S3A). LDN can comprise neutrophils at different maturation stages, and LDN transcriptional signature is indeed related to neutrophil progenitors or immature stages.[40] Because our sorting strategy was restricted to mature neutrophils, to unequivocally define the transcriptional signature of neutrophils derived from TET2^{Mut} HSC, we compared the gene expression program of our control and preleukemic neutrophils with independent RNA-seq datasets that also sort and analyze specific neutrophil subsets. We used one dataset identifying three neutrophil subsets (immature, intermediate, and mature) within the human bone marrow[41] and other dataset containing a detailed trajectory of neutrophil maturation in murine bone marrow.[5] In both datasets, the transcriptional signature of our bone marrow control neutrophils clustered closely to previously identified as mature neutrophils. Interestingly, TET2^{Mut} neutrophils clustered separately from their control human counterparts, and they were more closely associated with the transcriptional profile of immature neutrophils (Figures S3B and S3C). This is supported by the differences in genes involved in granule formation, where TET2^{Mut} neutrophils show an upregulation of genes involved in primary granules and a downregulation of genes involved in secondary and tertiary granule formation (Figure 3C), which is in line with the granule complexity analyzed previously (Figures 2C and 2D). Collectively, our results illustrate how TET2^{Mut} HSPCs produce neutrophils with both cellular and transcriptomic traits of immature neutrophils.

The chromatin of preleukemic neutrophils is hypermethylated and more compacted

TET2 catalyzes the demethylation of DNA, and thus, TET2 loss-of-function mutations found in individuals with CH are associated with widespread CpG hypermethylation in whole blood cells.[42,43] This hypermethylation pattern is also detectable in granulocytes from patients with TET2-mutated clonal cytopenia of undetermined significance (CCUS).[43] We thus performed methylation analysis to examine whether neutrophils derived from TET2^{Mut} hHSPCs exhibited CpG hypermethylation (Figure 3D), which is in accordance with the loss of TET2 protein expression (Figure S3D). The hypermethylated CpG bases were distributed across the genome (Figure 3E) and were found in different gene regions (Figure 3F; Table S3). As DNA

(C and D) Representative transmission electron microscopy (TEM) images of DAB-stained mature neutrophils sorted from bone marrow of humanized mice as indicated in (A). Red arrows show examples of primary (DAB-positive) granules, yellow stars secondary/tertiary (DAB-negative) granules. Scale bars, 2 μm. (D) Quantification of primary and secondary/tertiary granules. Data from 3 mice engrafted with control (gray) and 3 mice engrafted TET2^{Mut} (red/pink) HSPCs, at least 10 cells from each humanized mouse were analyzed (control n = 37, TET2^{Mut} = 40). Violin plots showing median and quartiles. One-way ANOVA test used for significance, ***$p < 0.001$; ****$p < 0.0001$.

(E) Mean fluorescent intensity (MFI) of CD63 in mature neutrophils (a.u., arbitrary units, raw data from the analyzer). Data from 1 experiment (control n = 6, TET2^{Mut} = 4). Each dot represents one humanized mouse and error bars: mean ± SEM.

(F and G) Immunocytochemistry staining of MPO and neutrophil elastase (NE) in control and TET2^{Mut} neutrophils. Fire lookup table, scale bars, 10 μm. (G) Quantification of signal per cell n = 3–4 mice per group. Violin plots showing median and quartiles. Unpaired t test used for significance.

(H and I) Schema (H) and flow gating strategy (I) to identify human neutrophils in chimeric mice engrafted with a 1:1 mix with control (black) or TET2^{Mut} (red) hHSCs. Schema created with BioRender.

(J) Percentage (right panel), SSC (center) and CD63-MFI (right) of control or TET2^{Mut} neutrophils within the same chimeric mouse. n = 6 mice, data representative of 3 experiments (CD63-MFI was evaluated in 1 chimeric mice experiment). Paired t test was used for significance, ns, no-significance, *$p < 0.05$, **$p < 0.01$.

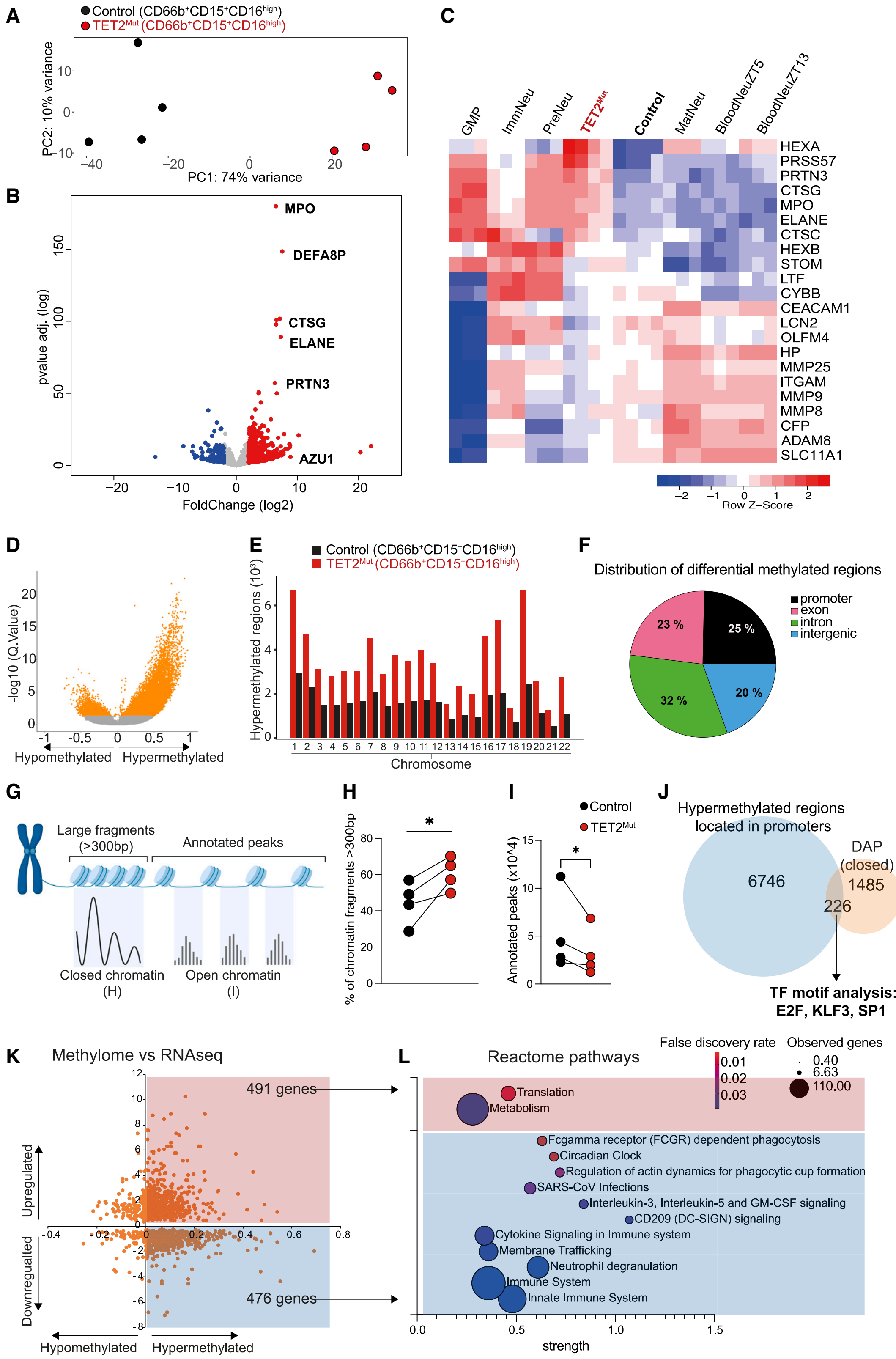

Figure 3. $TET2^{Mut}$ neutrophils are characterized by distinctive epigenome, chromatin architecture, and transcriptome signatures

(A) RNA-sequencing principal component analysis of control and $TET2^{Mut}$ sorted neutrophils ($CD66b^{+}CD15^{+}CD16^{high}$) (n = 4 mice).

(B) Volcano plot showing significantly (p value adjusted < 0.05) downregulated (blue dots) or upregulated (red dots) genes in $TET2^{Mut}$ neutrophils. See Table S2.

(legend continued on next page)

CellPress
OPEN ACCESS

methylation increases chromatin condensation, decreases overall DNA flexibility, and favors the heterochromatin state,[44] we next investigated the chromatin conformation of TET2^{Mut} neutrophils. Assay for transposase-accessible chromatin (ATAC) sequencing (Figure 3G) indicated that TET2^{Mut} neutrophils show more compact chromatin as exemplified by a reduction of annotated peaks in open chromatin regions and a higher proportion of large unfragmented chromatin (Figures 3H and 3I; Table S3). We then mapped the annotated peaks in both the control and TET2^{Mut} neutrophils genome. When analyzing the peaks annotated in gene promoters, we observed an enrichment for regions containing binding motifs for transcription factors (TFs) involved in different stages of neutrophil maturation, like Sp1, KLFs, CEBP1, or E2Fs (Table S3). Interestingly, although we obtained a higher proportion of KLF6 motifs in TET2^{Mut} neutrophils, a TF associated with early stages of neutrophil maturation, we observed a lower proportion of TF motifs associated with more mature neutrophils like CREB1 or E2F1 (Figure S3E). We also ran a differentially accessible peak (DAP) analysis to identify TF motifs that were associated with closed highly methylated promoter regions in TET2^{Mut} neutrophils. This correlative analysis indicated that transcriptional regulators involved in terminal neutrophil maturation such as Sp1, KLF3, or E2Fs[45,46] were less active in TET2^{Mut} neutrophils (Figure 3J; Table S3). Finally, although analyzing the genes with hypermethylated promoters and correlated them to their corresponding RNA levels (Figure 3K), we noticed a downregulation of several pathways related to phagocytosis, neutrophil activation, and response to infection in TET2^{Mut} neutrophils (Figure 3L; Table S3). Overall, granule content and transcriptome and epigenome analysis suggest that the loss of TET2 in HSPCs causes an accumulation of neutrophils blocked at the terminal differentiation step.

Neutrophil development from TET2^{Mut} HSPCs is characterized by transcriptional shifts to primitive stages

We next aimed to understand whether TET2 loss in HSPCs had a general effect on the continuum of neutrophil development beyond the block before the terminal maturation step. We sorted neutrophil precursors (preNeu: CD66b^{+}CD16^{-}CD117^{+}CD49d^{+}), immature neutrophils (immNeu: CD66b^{+}CD16^{low}CD117^{-}CD49d^{-}), and mature neutrophils (matNeu: CD66b^{+}CD16^{high}CD117^{-}CD49d^{-}) (Figure 4A). The three developmental stages clustered separately over a PCA from the preNeu stage toward the immNeu and matNeu at the end (Figure 4B). Recapitulating previous reports done in mice, enrichment pathway analysis revealed that preNeu have upregulated transcriptional programs associated with cell cycle and biosynthetic pathways and downregulate transcriptional programs related to immune effector functions characteristic of immNeu (granule formation) and matNeu (pathogen detection and phagocytosis) (Figure S4A; Table S4). Focusing on the impact of TET2 mutations, we first observed that the mutant counterparts clustered separately in each developmental stage, where the TET2^{Mut} population is always shifted toward the previous developmental stage (i.e., TET2^{Mut} matNeu are closer to immNeu and TET2^{Mut} immNeu closer to preNeu) (Figure 4B). For example, genes involved in the cell cycle (upregulated in preNeu) were enriched in TET2^{Mut} immNeu compared with control immNeu (Figure S4A). We can also appreciate that TET2^{Mut} matNeu shows less upregulation of the characteristic pathway/genes of the mature stage, in accordance with our previous analysis (Figure 3C). To further confirm that TET2 mutations were associated with primitive stages in each cell transition, we cross-compared the transcriptional differences that account for the transition from preNeu to immNeu or from immNeu to matNeu with the transcriptional differences associated with the TET2 mutation in immNeu or matNeu, respectively (Figures 4C, 4D, and S4B). In these cross-comparisons, we observed a general correlation, which for each cell transition, the TET2^{Mut} is more associated with a more primitive stage. For example, pathways upregulated or downregulated in each cell transition appear to follow an opposite pattern when assessing the impact of TET2 mutations in each cell type (Figure 4C). Also, when we cross compare the genes that were upregulated or downregulated by TET2 together with the genes differentially expressed in cell transition, we observed that genes associated with preNeu are significantly upregulated in TET2^{Mut} immNeu (Figure 4D; Table S4). Similarly, most of the genes upregulated in TET2^{Mut} matNeu are enriched in immNeu cell state (Figure S4B; Table S4). Overall, these data indicate that the loss of TET2 in HSPCs rewires the neutrophil development stages providing distinct transcriptome profiles that, for each stage, are associated with a more primitive population.

(C) Heatmap representing the expression of genes associated with primary, secondary, and tertiary granule formation. The expression of these genes in our control and TET2^{Mut} neutrophils was compared with the expression in the different neutrophil subsets identified in GSE109467.

(D) Volcano plot of differentially methylated CpG bases. Orange colored dots indicate adjusted p value < 0.05.

(E) Bar plot showing the distribution of the differentially methylated regions across the chromosomes.

(F) Distribution of the differentially methylated regions according to their location in promoter, exon, intron, or intergenic regions. See Table S3.

(G) ATAC-seq schematic representation of the distinguishable closed and open chromatin regions. For this experiment we used and compared control and TET2^{Mut} neutrophils sorted from the same mouse engrafted with a mix of wild-type and TET2-mutant hHSPCs.

(H) Comparison of the percentage of large chromatin fragments (>300 bp) between control and TET2^{Mut} neutrophils from the same mouse. Paired t test used for significance, *$p < 0.05$. See Table S3.

(I) Comparison of annotated peaks between control and TET2^{Mut} neutrophils from the same mouse. Paired t test used for significance, *$p < 0.05$. See Table S3.

(J) Venn diagram showing that 226 gene promoter regions were found both hypermethylated and less accessible in the differential accessible peak (DAP) analysis. These promoter regions were enriched in binding motifs associated with E2F, KLF3, or SP1 transcription factors. See Table S3.

(K) Overlap of differentially expressed genes identified in the RNA-seq analysis and genes containing differentially methylated promoter identified in the methylome sequencing. Numbers in the corners indicate the number of genes differentially regulated and containing hypermethylated CpG sites in the promoters. See Table S3.

(L) Selection of differentially regulated pathways in reactome using the genes containing hypermethylated CpGs in the promoters that were upregulated (in red) or downregulated (blue) in the RNA-seq. See Table S3 for complete list of pathways.

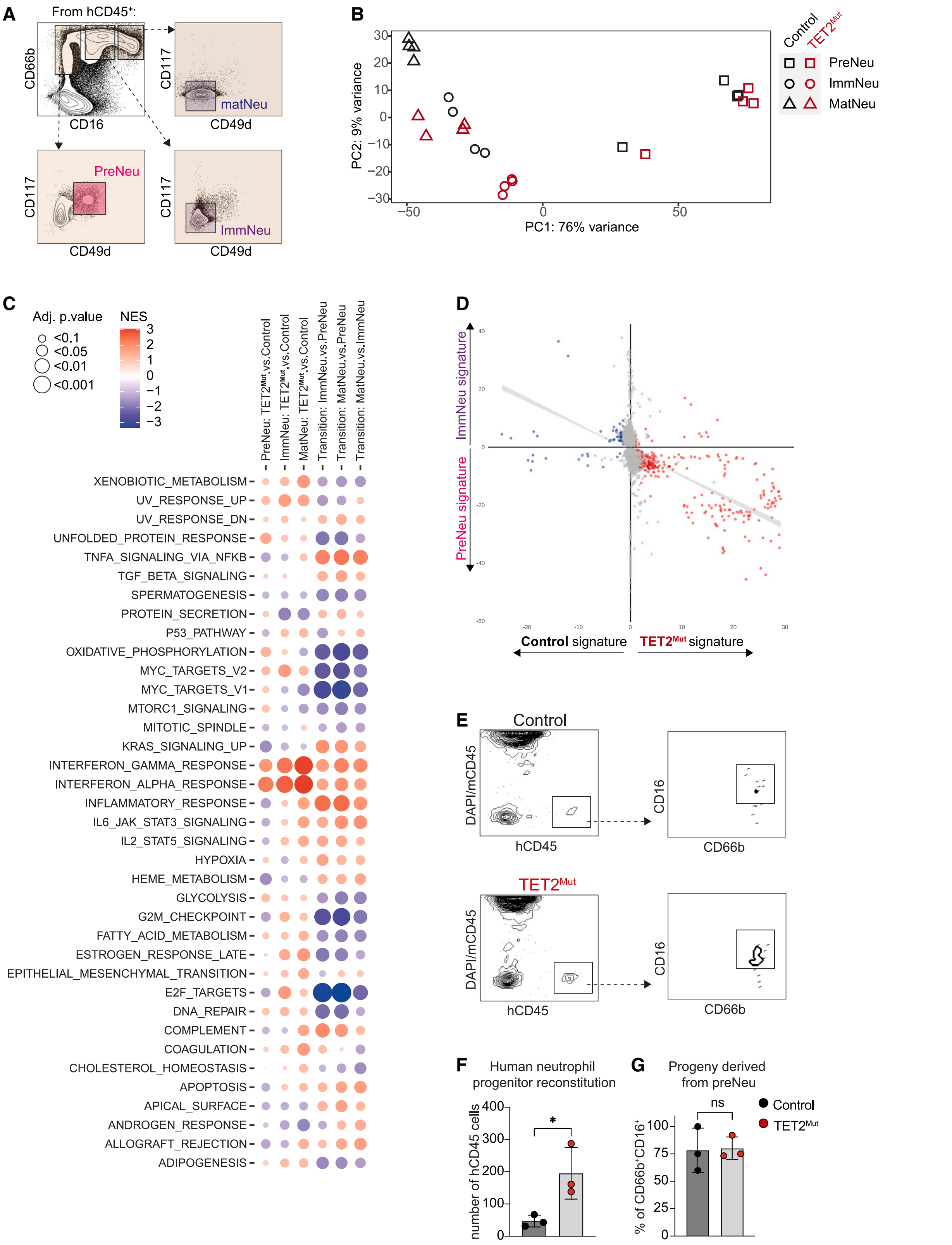

Figure 4. TET2 mutations alter the transcriptome of different neutrophil developmental stages and increase neutrophil progenitor potential

(A) Representative sorting strategy to isolate from bone marrow human CD45$^+$ cells: preNeu (CD66b$^+$CD16$^-$CD117$^+$CD49d$^+$), immNeu (CD66b^{+-}CD16^{mid}CD117$^-$CD49d$^-$), and matNeu (CD66b$^+$CD16^{high}CD117$^+$CD49d$^+$).

(legend continued on next page)

Loss of TET2 confers higher repopulating capacity to neutrophil progenitors

Considering the transcriptomic shift toward primitive stages and the increased percentage of human neutrophils present in the BM and peripheral tissues of mice engrafted with TET2^{Mut} hHSPCs and the fact that TET2 mutations have been reported to confer repopulating advantage to HSPCs,[47–49] we sought to determine if TET2 mutations could also impact on the capacity of neutrophil progenitors to generate neutrophils. To analyze the functionality of neutrophil progenitors, we performed an adoptive transfer of control or TET2^{Mut} human neutrophil progenitors sorted from primary immunodeficient mice (Figures S4C and S4D) into secondary recipient mice and analyzed the human engraftment after 3 days (Figure S4E). The human reconstitution found in the secondary recipients from neutrophil progenitors was higher in TET2^{Mut} compared with recipients of TET2^{WT} control cells (Figures 4E and 4F). As expected, the human hematopoietic system constituted mainly of differentiated neutrophils in both recipients (Figures 4E and 4G). We also proved the higher potential of TET2^{Mut} neutrophil progenitors by *in vitro* colony-forming unit (CFU) assay, where we observed a significant increase of colonies derived from TET2^{Mut} progenitors (Figure S4F). These results indicate that TET2 mutations confer a proliferative advantage on neutrophil progenitors.

TET2^{Mut} neutrophils exhibit decreased phagocytic capacity and exacerbated inflammatory responses

Based on the transcriptomic and epigenomic analysis indicating downregulation of different immune response pathways in TET2^{Mut} neutrophils, we investigated the impact on their canonical function. To perform different functional assays *ex vivo*, we first sorted control neutrophils from bone marrow of humanized mice and tested whether these human CD66b^{+}CD15^{+}CD16^{hi} cells would respond to known NETosis or phagocytosis stimuli. As described for mature human neutrophils isolated from peripheral blood of healthy donors (HDs), we found that neutrophils from humanized mice were able to phagocytize *C. albicans* yeast and formed NETs upon PMA (phorbol 12-myristate 13-acetate), *C. albicans* hyphae, or cholesterol crystal stimulation (Figure S5A). We then investigated the capacity of preleukemic neutrophils to sense and respond to pathogens *ex vivo* and *in vivo*. First, *ex vivo*, consistent with the downregulation of phagocytosis receptors (Figure 3L; Table S2), we observed a significant reduction in the phagocytic capacity of TET2^{Mut} neutrophils against zymosan fluorescent beads or *C. albicans* yeast (Figures 5A, 5B, and S5B). Then, we analyzed the transcriptional response to LPS stimulation. Interestingly, the inflammatory response was hyperactivated in TET2^{Mut} neutrophils exemplified by the increased expression of different chemokines such as CCL7 and CXCL2 (Figures 5C, 5D, S5C, and S5D; Table S5). Of note, we also noticed that classic pro-inflammatory cytokines such as IL1β or IL6, reported to be upregulated in macrophages from TET2-KO mouse models,[21] appear to be upregulated in TET2^{Mut} human neutrophils. Then, we tested *in vivo* some of these observations. In accordance with the increase of inflammatory cytokine induction, TET2^{Mut} human neutrophil recruitment *in vivo* was significantly increased in the lungs, 24 h after intranasal LPS exposure (Figures 5E and 5F). We also attempt to test *in vivo* the response to live pathogens such as *C. albicans*. We administrated *C. albicans* intranasally and analyzed neutrophil recruitment and the presence of *C. albicans* in the lung after 24 h of the exposure. Consistent with the observations in the pulmonary LPS challenge model, the recruitment of human neutrophils was also increased in mice reconstituted with TET2^{Mut} hHSPCs (Figure S5E). We also noticed a trend toward an increased presence of *C. albicans* 24 h after exposure (Figures 5G, 5H, and S5F), which would suggest a defect in the phagocytosis of TET2^{Mut} neutrophils, in line with the phagocytosis analysis done *ex vivo*. Overall, our results show that TET2 mutations in hHSPCs interfere with the ability of derived neutrophils to sense and respond to microbes and mount a balanced inflammatory response to sites of infection.

TET2^{Mut} neutrophils display altered NET architecture and function

We then analyzed the capacity of TET2^{Mut} neutrophils to undergo NETosis. PMA-stimulated control and mutant neutrophils were fixed at different time points post-stimulation. Confocal imaging of NE, MPO, and proteinase 3 (PR3) displayed the typical stages of NETosis, such as migration of NE to the nucleus, nuclear decondensation, and NET formation decorated with granular proteins such as MPO or NE[28,50] (Figure S5F). These results confirmed that both control and TET2^{Mut} neutrophils generated in humanized mice are capable of undergoing NETosis. Nevertheless, the differences in chromatin architecture (Figures 3G–3I) and nuclear morphology (Figures S5G and S5H) lead us to hypothesize that TET2^{Mut} neutrophils may produce distinctive NETs. To this end, we analyzed the properties of NETs released by TET2^{Mut} neutrophils in greater detail using live-cell imaging. NETs released by TET2^{Mut} neutrophils were more condensed and displayed a brighter sytox signal (Figure 6A; Mendeley Data S1). We used two independent image-analysis methods to quantify the area of the NETs based on the sytox signal, and we concluded that NETs produced by TET2^{Mut} neutrophils had a significantly smaller area compared with the NETs produced by control neutrophils (Figures 6B and S6A). Interestingly, upon PMA stimulation, control and TET2^{Mut} neutrophils showed no major differences in the levels of ROS,

(B) RNA-sequencing principal component analysis of the different neutrophil populations from mice reconstituted with control and TET2^{Mut} hHSPCs (n = 4 mice.).
(C) Gene-set enrichment analysis using Hallmark database for the different comparisons displayed in the columns. See Table S4.
(D) Scatterplots comparing the fold changes of genes with adjusted p values of ≤ 0.05 in the preNeu vs. immNeu (y axis) comparison with the fold changes of genes with adjusted p values of ≤ 0.05 that appear downregulated (blue) or upregulated (red) in TET2^{Mut} immNeu (x axis).
(E–G) Representative density plots from the secondary recipient mice engrafted with control or TET2^{Mut} neutrophil progenitors (E). Number of human CD45 cells (F) and percentage of CD66b^{+}CD16^{+} (G) in reconstituted secondary recipient mice. Each dot represents one secondary recipient mouse. 3 secondary recipient mice per group, each received the neutrophil progenitors from different primary humanized mouse, and error bars: mean ± SEM. Unpaired t test used for significance, *$p < 0.05$.

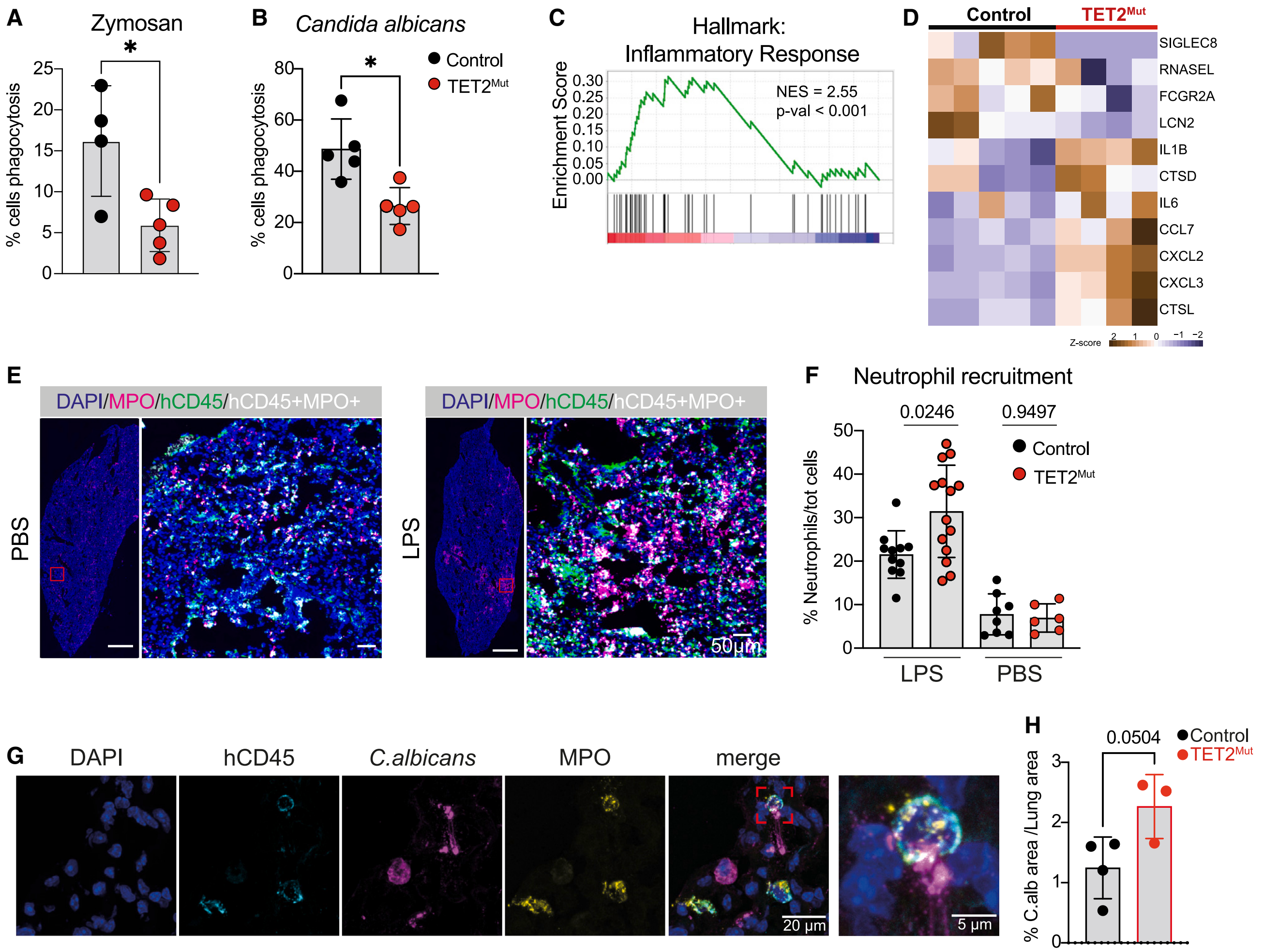

Figure 5. TET2^{Mut} neutrophils show exacerbated inflammation and recruitment to sites of infection

(A and B) Percentage of control or TET2^{Mut} human neutrophils undergoing phagocytosis of zymosan 488 beads (A) or yeast *C. albicans* (B) (n = 4–5 per group, representative of 3 independent experiments). Mann-Whitney statistical test used for significance, $^*p < 0.05$.

(C) Gene-set enrichment analysis (GSEA) showing upregulation of inflammatory response pathway using the Hallmark database. See Table S5 for complete list of pathways.

(D) Heatmap of selected genes differentially expressed (p value < 0.05) in TET2^{Mut} neutrophils after LPS stimulation (3 h, 1 µg/mL). See Table S5 for complete list of differentially expressed genes.

(E) Immunohistochemistry image of a lung section from a mouse engrafted with control hHSPCs challenged with PBS (top row) or LPS (bottom row) intranasally. Overview of a control lung section (left) with DAPI (blue) and MPO (magenta) staining, scale bars, 100 µm. Magnification of a region of interest of the lung with DAPI (blue), human CD45 (green), MPO (magenta), and cells containing CD45 and MPO signal (white) scale bars, 50 µm.

(F) Quantification of percentage of neutrophil infiltration (%MPO+ cells) normalized to the total number of cells in the tissue. Each dot represents a lung section. 3–4 sections were used per lung (n = 3–4 mice in LPS group and n = 2–3 mice in PBS group). Mann-Whitney test used for significance.

(G) Confocal immunohistochemistry images of a lung section from a mouse engrafted with control hHSPCs challenged with *C. albicans* showing human CD45+ MPO+ neutrophils attached to a *C. albicans* hyphae and phagocytosing a *C. albicans* yeast with MPO colocalized. DAPI (blue), hCD45 (cyan), *C. albicans* (magenta), and MPO (yellow). Scale bars, 20 and 5 µm.

(H) Quantification of the percentage of *Candida albicans* signal over the total lung area in control and TET2^{Mut} mice. Mean of 2–3 sections per mouse, unpaired t test used for significance.

and no significant differences were detected at the time of NETosis or the percentage of NETotic cells (Figures S6B–S6E), indicating that NET architecture is the main feature disrupted by TET2 mutations. These observations were further supported by scanning electron microscopy where we observed that TET2^{Mut} neutrophils released NETs that had a smaller surface area (Figures 6C and S6F). Since NETosis is a key innate immune response against fungal hyphae,[51–53] we tested how TET2 mutations in neutrophils impact on *C. albicans* hyphal growth control. Interestingly, TET2^{Mut} neutrophils displayed impaired control of the fungal growth (Figure 6D). These findings indicate that human neutrophils derived from TET2^{Mut} HSCs have a distinctive chromatin architecture that correlates with aberrant NETs that can be less efficient to fight fungal infections.

Altogether, our data indicate that human neutrophils derived from TET2^{Mut} HSCs have lower antimicrobial effector capacity

CellPress
OPEN ACCESS

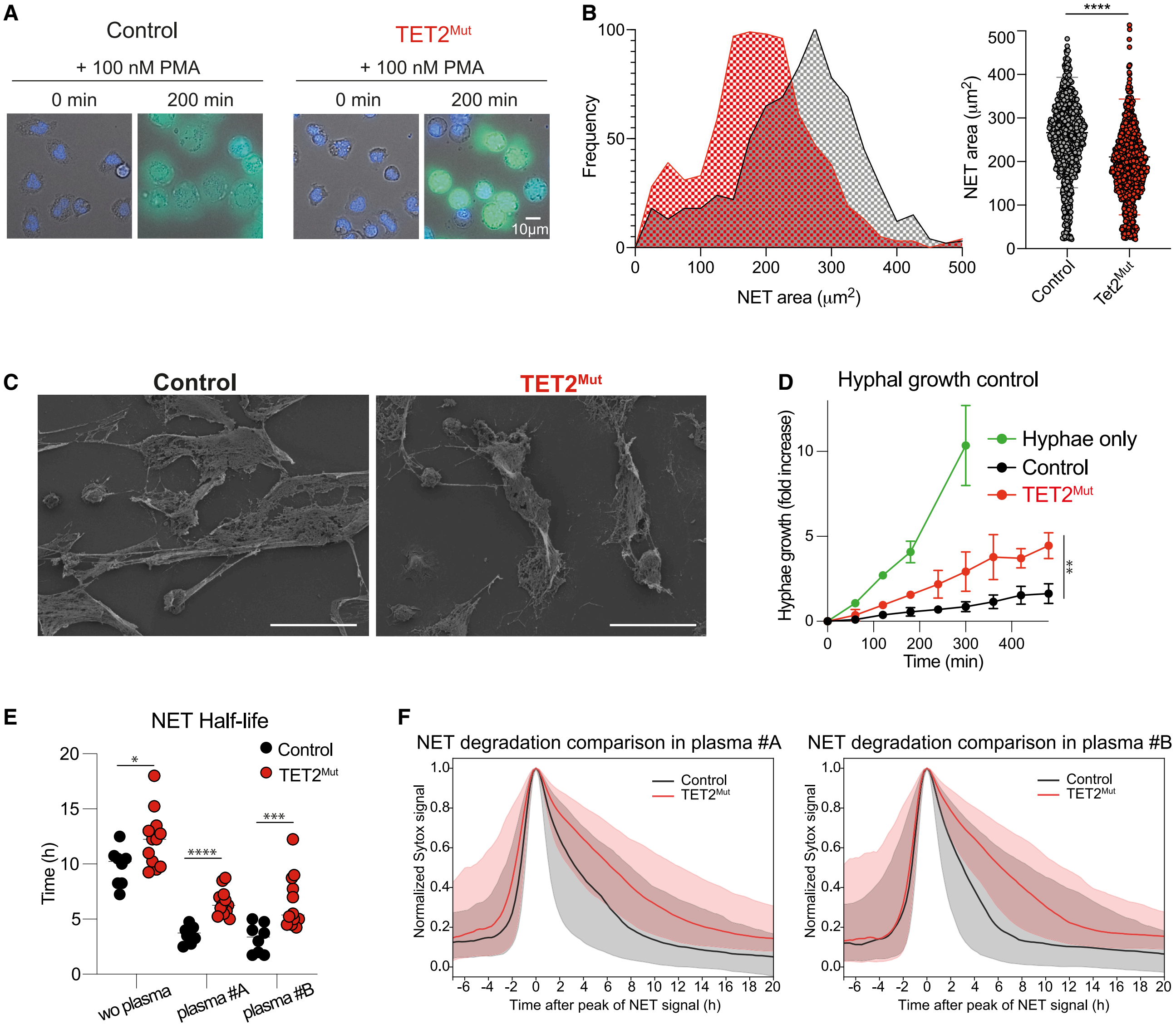

Figure 6. NETs produced by neutrophils derived from TET2^{Mut} HSCs are more compact and difficult to be degraded

(A) Representative magnification images of wide-field fluorescence microscopy from time-lapse imaging of PMA-stimulated control and TET2^{Mut} neutrophils at time point 0 and 200 min. Nucleus are stained with DAPI and NETs are stained with sytox green. Scale bars, 10 μm.

(B) Frequency versus NET area plot displaying the distribution of NET areas of control and TET2^{Mut} neutrophils quantified at time point 200 min (left panel). Distribution of NET area of individual cells in control (gray) and TET2^{Mut} (red) neutrophils (right panel). n = 3 per group, representative of 4 independent experiments. Mann-Whitney statistical test used for significance, ****$p < 0.0001$.

(C) Scanning electron microscopy images of NETs formed upon 100 nM PMA stimulation from control or TET2^{Mut} neutrophils. Scale bars, 30 μm.

(D) Hyphal growth fold increase over time of *C. albicans* hyphae only (green line), hyphae co-cultured with control neutrophils (black line), and TET2^{Mut} neutrophils (red line). n = 3 per group, Wilcoxon matched pair signed rank test, *$p < 0.05$; **$p < 0.01$.

(E) NET half-life values from NET traces obtained by gathering the signal over time of 1,000 randomly sampled pixels previously classified as NET by the pixROI method, median traces were calculated, and the half-life values of the median traces per field of view corresponding with the traces shown in Figure S6H and (F). Mann-Whitney statistical analysis, *$p < 0.05$; **$p < 0.01$; ***$p < 0.001$; ****$p < 0.0001$.

(F) Comparison of NET degradation profiles for control and TET2^{Mut} neutrophils in the presence of different human plasma. Median curves (solid lines) of netotic pixel signals aligned and normalized to their peak as classified by the pixROI method. Shaded regions are bounded by the Q1 and Q3 curves corresponding to the 25% and 75% quartiles. The specific times in plasma #A (left panel) were 210 min (Q1 = 75 min; Q3 = 495 min) for the control NETs and 390 min (Q1 = 180 min; Q3 = 735 min) for the TET2^{Mut} NETs; and for plasma #B (right panel) were 195 min (Q1 = 90 min; Q3 = 525 min) for the control NETs and 390 min (Q1 = 225 min; Q3 = 690 min) for the TET2^{Mut} NETs. The curves are computed on signals gathering 1,000 randomly sampled pixels for all videos corresponding to the mouse type and experimental condition.

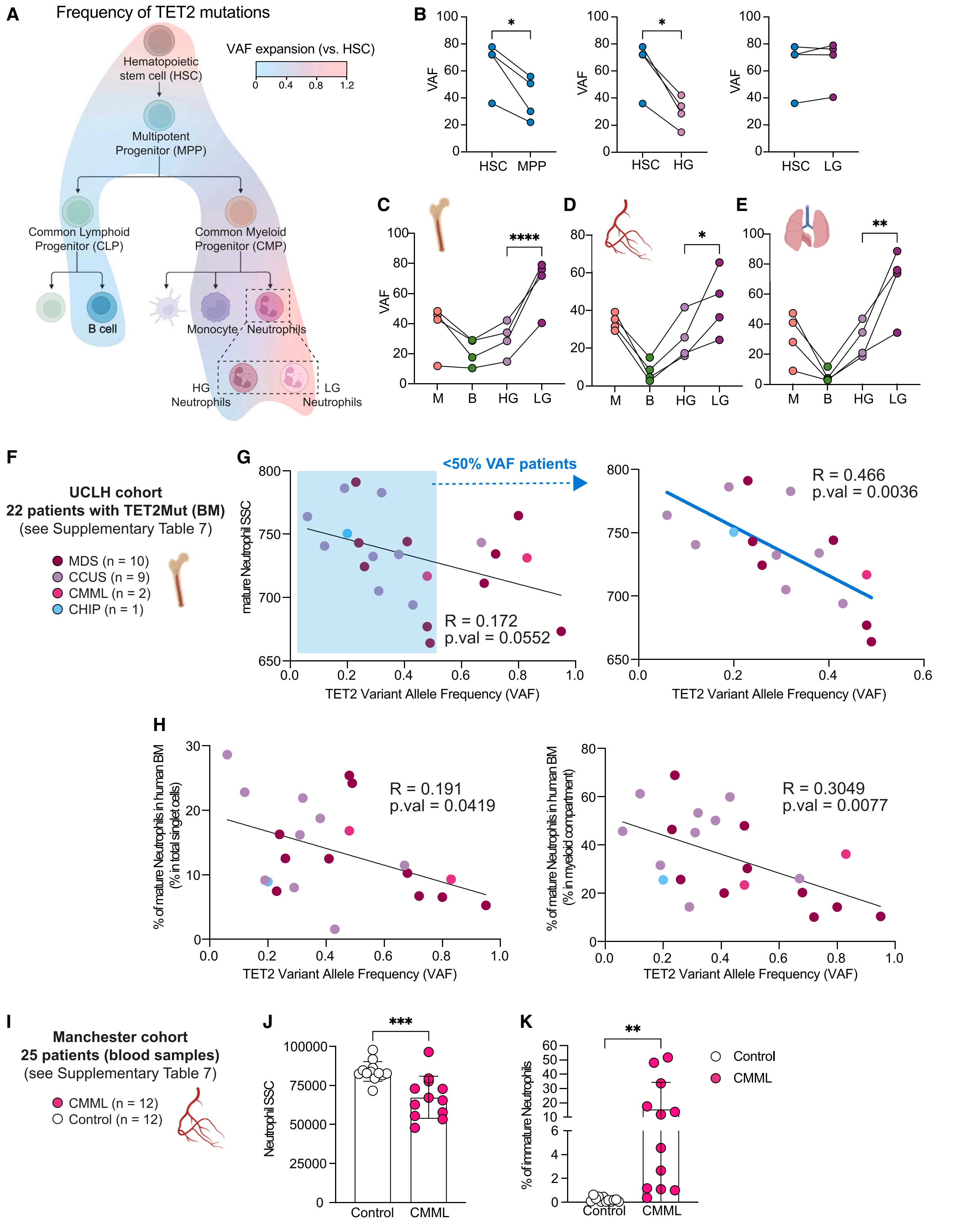

(legend on next page)

and have a higher tendency to produce inflammatory cytokines that were associated with higher neutrophil infiltration in the lung. Both the impairment in phagocytic and NET-mediated fungal control in $TET2^{Mut}$ neutrophils are consistent with the increased susceptibility to microbial infections described in individuals with TET2 mutations.[16]

NETs produced by $TET2^{Mut}$ neutrophils are cleared less efficiently by plasma nucleases

Uncontrolled NET formation or deficiencies in NET clearance have been linked to multiple NET-mediated pathologies.[54] Considering that $TET2^{Mut}$ neutrophils produce smaller and more compact NETs and that chromatin from $TET2^{Mut}$ displays decreased endonuclease accessibility (Figures 3H and 3I), we investigated possible differences in NET degradation by the circulating nucleases present in human plasma from HDs via time-lapse fluorescence microscopy. We first validated that HD plasma, which contains the endonucleases DNase-I and DNase IL3, degraded NETs generated by human neutrophils isolated from the bone marrow of humanized mice (Figure S6G). Next, we compared the stability of NETs generated by control and $TET2^{Mut}$ neutrophils. In the absence of nucleases, $TET2^{Mut}$ NETs were slightly more stable compared with control NETs with a half-life of 9.75 h for control NETs and 12 h for $TET2^{Mut}$ NETs (Figures 6E and S6H; Mendeley Data S1). These small differences in NET degradation were augmented in the presence of human plasma. When 3% of plasma from two different donors (A and B) was added to the medium, the half-life of NETs was 3.5 (plasma A) and 3.25 h (plasma B) for the control NETs and 6.5 h (plasma A and B) for the $TET2^{Mut}$ NETs (Figures 6E and 6F; Mendeley Data S1; Table S6). We also observed that $TET2^{Mut}$ NETs were more stable than control NETs when exogenous bovine pancreatic DNase-I was added. In the presence of 5×10^{-6} U/μL DNase-I the half-life of control NETs was 4.8 h compared with 6.8 h for $TET2^{Mut}$ NETs (Figure S6I).

Overall, these results indicate that NETs produced by $TET2^{Mut}$ neutrophils are more resistant to degradation by circulating nucleases, suggesting that they may persist longer and drive inflammation. These findings uncover an important role for TET2 mutations in regulating NET clearance that may contribute to the higher risk of individuals with CH for developing NET-mediated pathologies such as atherosclerosis or COPD.

TET2-derived human clonal hematopoiesis is enriched in HSCs and LG neutrophils

We finally sought to determine the potential use of the distinct morphological trait of preleukemic neutrophils as a strategy to detect somatic mutations in the physiological scenario of CH in humans. To this end, we injected hHSPCs with a TET2 variant allele frequency (VAF) ≈ 20% and analyzed the VAF expansion from HSCs in different human stems, progenitors, and differentiated cell populations of the hematopoietic tree in the BM (Figures 7A and S7A). Strikingly, the highest VAF was detected in HSCs and LG neutrophils (Figures 7A and S7B). Indeed, the frequency of TET2 mutation dropped in multipotent progenitors (MPPs), and over the progenitor compartment, and was particularly low in the lymphoid lineage (Figures 7A and S7B). Nonetheless, similar VAF levels were observed between HSCs and LG neutrophils, in contrast to HG neutrophils where the VAF level was reduced (Figure 7B). These results conciliate the notion that TET2 mutations confer an advantage to HSC by not only enhancing self-renewal but also causing a myeloid differentiation skewing.[49,55–57] Importantly, we confirmed that LG neutrophils carried the highest percentage of TET2 mutations among the immune cell types analyzed in not only the BM but also the lung or blood circulation (Figures 7C–7E) constituting a suitable cell candidate to estimate the frequency of mutations in the hardly accessible HSPC compartment. Overall, our data highlight the differentiation bias of $TET2^{Mut}$ hHSPCs and the potential utility to screen for LG neutrophils to obtain a faithful readout of the mutation frequency in patients. To validate these observations in primary human samples, we obtain data from two independent cohorts of human patients previously diagnosed with hematological diseases carrying TET2 mutations (Figures 7F and 7I). The first cohort at the University College London Hospital (UCLH) cohort was composed of 22 patients with a different degree of hematological disorders (from CH to MDS) that only carry mutations in the TET2 gene (Table S7). We analyzed the neutrophils from the bone marrow of these patients, and we observed a significant correlation between the frequency of TET2 mutations

Figure 7. TET2-derived human clonal hematopoiesis is associated with the appearance of low-granule neutrophils

(A) Overlayed heatmap into the hematopoietic tree showing the fold expansion of TET2 mutations from HSCs. The following cell populations were analyzed (HSCs, MPP, CLP, CMP, GMP, B cells, monocytes, and HG and LN neutrophils). Data representative of 4 humanized mice. See Figure S7A for sorting strategy and Figure S7B for raw data of VAF for each cell population in each humanized mice.

(B) Comparison of the VAF between HSC and MPP (left panel), high granule (HG) neutrophils(center), and low-granule (LG) neutrophils (right). Each line of connected dots represents one humanized mouse. Paired t test used for significance, $^*p < 0.05$.

(C–E) Percentage of TET2 mutations in different immune cell populations from bone marrow (C), blood (D), and lung (E). Monocytes (M), B cells (B), high granule (HG) neutrophils, and low-granule (LG) neutrophils were sorted from 4 humanized mice engrafted with hHSCs from different human donors, each line of connected dots represents one mouse. Two-way ANOVA test used for significance, $^*p < 0.05$; $^{**}p < 0.01$; $^{****}p < 0.001$.

(F) UCLH cohort consisted of BM aspirates for routine diagnostic purposes at University College London Hospitals (UCLH). 22 patients with hematological disorder diagnosed were identified with mutations of TET2 but who had no other mutations.

(G) Correlation between the median side scatter (SSC) of bone marrow mature neutrophils and the TET2 variant allele frequency (VAF) of these 22 patients (left panel) or the correlation for the patients with less than 50% TET2 VAF (right panel). See Figure S7C for gating strategy. Significant correlation was measured by linear regression analysis.

(H) Correlation between the percentage of bone marrow mature neutrophils within total BM cells (left panel) or within the myeloid compartment (right panel) and the TET2 variant allele frequency (VAF) of these 22 patients. See Figure S7C. Significant correlation was measured by linear regression analysis.

(I) Manchester cohort consisted of peripheral blood samples from CMML patients (n = 12) or healthy volunteers (n = 12) obtained from the Manchester Cancer Research Centre Tissue Biobank.

(J) Side-scatter of neutrophils isolated by immunomagnetic negative isolation from peripheral blood of healthy volunteers or CMML patients. See Figure S7D.

(K) Percentage of the immature neutrophil fraction ($CD66b^{+}CD16^{-/low}$) within the neutrophil population isolated from peripheral blood. See Figure S7D.

and the granule complexity of the neutrophils (Figure 7G, left), validating our observation in the humanized mouse model. Of note, for patients with VAF lower than 50%, which resembles more accurately the common scenario found in individuals with CH, this correlation was more pronounced and significant (Figure 7G, right). The second patient cohort from the Manchester Hospital cohort was composed of 12 patients diagnosed with chronic myelomonocytic leukemia (CMML) and 12 healthy control individuals. We analyzed neutrophils from the peripheral blood samples of these patients. In this cohort, all 12 CMML patients carried not only TET2 mutations but also mutations in other genes commonly affected in hematological disorders (Table S7). Despite the variability and the presence of other mutations, we observed a significant reduction in the granule complexity of neutrophils from CMML patients (Figure 7J). In line with our findings in the humanized mouse model, data from these cohorts also confirmed that TET2 mutations were associated with altered neutrophil development in humans (Figures 7H and 7K). In the UCLH cohort, we observed a decrease in phenotypically defined $CD33^{+}CD117^{-}CD10^{+}$ cells in the bone marrow (Figures 7H and S7C), and in the Manchester cohort, we observed the appearance of immature neutrophils ($CD66b^{+}CD16^{low/-}$) in peripheral blood (Figures 7K and S7D).

DISCUSSION

In this work, we combined CRISPR-mediated genetic engineering of hHSPCs and transplantation in immunodeficient mice to evaluate human neutrophil development and the functional impact associated with TET2 mutations. Loss-of-function TET2 mutations are one of the most common and prevalent mutations found in CH scenarios and constitute a risk factor for the development of hematological malignancies. Hence, mutations affecting TET2 are commonly referred to as preleukemic mutations. Humanized mice have traditionally been used for the study of human HSC functionality and hematological malignancies as patient-derived xenograft models.[58,59] The recent characterization of human DC subsets[60] and the development of human lung macrophages from bone marrow monocytes[61] are good examples of the potentiality of using humanized mice as a tool to also elucidate the development and functionality of different human immune cell types. A recent report has used humanized mice to validate ELANE as a key factor for neutrophil maturation.[62] We have used this model to analyze the effector immune functions of neutrophils and the neutrophil heterogeneity in bone marrow and peripheral tissues derived from genetically modified human HSCs. Focusing on TET2, we show that neutrophils constitute a high-frequent TET2-mutant cell subset among the hematopoietic tree in the different tissues analyzed (BM, blood, and lung), and we provide valuable insights into the cellular heterogeneity that individuals with TET2-derived CH likely display, and how we can improve diagnostic strategies based on such differences. Specifically, we show that TET2 mutations cause an increase in the capacity of neutrophil progenitors to generate differentiated neutrophils, which are characterized by LG complexity and with a distinct transcriptome and epigenome identity related to immature stages. We also provide evidence that patients carrying TET2 mutations have neutrophils with LG complexity and a reduced number of mature neutrophils. Our results aid to understand the recent association established between individuals that have CH or other blood disorders with clinical traits affecting neutrophils such as agranulocytosis and a lower count of mature neutrophils.[63–66] The role described here that TET2 plays in human neutrophil development seems to be conserved through evolution, since our findings are in line with recent reports describing that TET3 and TET3/TET2 double-KO zebrafish show defective granule formation in neutrophils.[67]

Loss of TET2 has been reported to cause a myeloid bias in mice.[49,68,69] Our findings confirm a similar scenario in humanized mice and go further to show that human HSCs not only have a preferential differentiation toward myeloid lineage but also an accentuated granulocytic bias. Indeed, the exacerbated granulopoiesis coupled with the immature signature observed in neutrophils derived from $TET2^{Mut}$ HSPCs recall stress-induced hematopoiesis scenarios such as bone marrow transplantation or infections, where LDNs accumulate. Along these lines, we indeed observed that $TET2^{Mut}$ neutrophils share transcriptomic similarities to LDN.[41] It is intriguing to speculate that HSPCs carrying preleukemic mutations could be developing under a "low-grade" hematopoietic stress, where, although perfectly able to generate all differentiated cells, they do so with myeloid/granulocytic bias shaping their immune effector functions. As an example, it has been reported that macrophages with TET2 mutations show an exacerbated production of classic pro-inflammatory cytokines such as IL1β and IL6 in response to LPS.[21] Similarly, our results show that $TET2^{Mut}$ neutrophils have an upregulated production of IL6 and IL1β and could contribute to the exacerbated inflammatory milieu reported in CH individuals. In addition, the increased levels of CCL7 or CXCL2 and other chemoattractants in $TET2^{Mut}$ neutrophils might also boost the recruitment of other myeloid cells and their associated pathological inflammation. Future investigation is needed to understand how $TET2^{Mut}$ neutrophils (or neutrophils carrying preleukemic mutations in CH patients) will undergo transcriptional reprogramming upon other/different environmental stress signals. Recent evidence suggests that transcriptional adaptation in neutrophils occurs at the cell heterogeneity level and in a tissue-specific manner,[41,70] and thus, we speculate that preleukemic neutrophils will have a different transcriptomic plasticity in the bone marrow and peripheral tissues, and their stress-dependent responses will differ from the healthy/non-mutated neutrophils, shaping the immune response of CH patients.

In contrast to the augmentation of inflammatory responses, TET2 mutations suppress key neutrophil antimicrobial effector functions such as phagocytosis and NETosis. $TET2^{Mut}$ neutrophils are less able to phagocytize small microbes such as yeast and control hyphal growth via NETosis. Moreover, the aberrant more condensed NETs generated by these mutant neutrophils are cleared less efficiently by plasma DNases. Clearance of NETs is a key step of the NETosis cycle that is required to prevent excessive inflammation and tissue damage.[29] For this, the impact of TET2 on NET clearance provides a paradigm for how post-translational chromatin modifications, such as those regulated by demethylating enzymes such as TET2, may regulate inflammation not only by influencing the affinity of chromatin for TLR4 as reported recently[71] but also by extending NET lifespan. The combined impact of TET2 mutation on reduced NET

antimicrobial capacity and increased lifespan may further shift the balance toward pathology by extending the course of infection while interfering with NET clearance. Beyond the impact upon pathogen infection, reduced NET clearance constitutes a potential molecular candidate to understand why CH individuals have a higher risk of developing atherosclerosis and cardiovascular pathologies. Indeed, more stable NETs produced by TET2^{Mut} neutrophils might foster vasculature occlusion and promote platelet activation and aggregation, facilitating thrombus formation. In addition, our work contributes to start untangling the recent association established between individuals with TET2 mutations and COPD,[17] a disease highly linked to airway neutrophilia and alterations in neutrophil degranulation and excessive NET presence.[72,73] On these lines, we report here together with the extended NET presence the increased neutrophilia in the lung and alterations in granule content derived from TET2 mutations. Nevertheless, to directly link the development of COPD or atherosclerosis with the aberrant function of human TET2^{Mut} neutrophil, new disease models in the background of immunodeficient mice will need to be developed. In such humanized mouse models, it will remain a challenge to elucidate the different contributions to the pathology of the different "preleukemic immune cells," as all the immune progenies will inherit TET2 mutations from HSPCs. Future lines of investigation should also address the analysis of whether reconstituting the loss of TET2 activity rescues the phenotype of neutrophils and their immune effector functions. For example, ascorbate has been reported to rescue TET2 deficiency phenotypes in HSCs.[48,74] Thus, it is exciting to speculate that treatment with ascorbate might help to reduce the aberrant neutrophil development and NET formation to ultimately reduce the risk to develop NET-mediated pathologies.

Overall, our findings place neutrophils and NETs as cellular and molecular players in the aberrant feedback loop model proposed to take place in the CH stage. Moreover, we hypothesize that beyond the cell-autonomous impact of preleukemic neutrophils on the pathology of CH individuals described here, the aberrant immune function of mutant neutrophils could also affect the response of other immune players such as monocytes/macrophages. For example, the putative pro-inflammatory properties of the "preleukemic NET" or the possibility that the aberrant NETs released by preleukemic neutrophils could exacerbate/dampen macrophage responses remain open questions of the investigation. Finally, the fact that the reduced granule complexity and immature pattern described in our humanized mice model were validated in two independent human patient cohorts supports the use of this model to further investigate potential therapeutic targets.

Limitations of the study

We used NSG-SGM3 cKit$^{w41/w41}$ mice to generate our humanized mice and study the impact of TET2 loss in human HSCs. Therefore, the impact of TET2 mutations we report using this mouse strain could be influenced by two stress scenarios. The first stress is associated with HSC transplantation and repopulation procedure despite being reduced as no conditioning treatment is necessary for this mouse model. The second one is associated with the presence of high expression of human SCF, IL-3, and GM-CSF, which allow for robust myeloid human differentiation but may contribute to hematopoietic stress. In this regard, we performed initial experiments in NSG-cKit$^{w41/w41}$ and obtain similar observations, ruling out the supra-physiological level of human cytokines as the main cause of the differences seen in TET2^{Mut} HSCs. Then, due to the reduced yield of human myeloid and neutrophil reconstitution, we mainly work with NSG-SGM3 cKit$^{w41/w41}$ mice. Moreover, we also provided data from two independent human cohorts that validate part of the phenotype described in the mouse model (granule complexity and imbalance of immature/mature neutrophils). Therefore, the experimental conditions mentioned above might, if something, further exacerbate the impact of TET2 mutations. In the future, it would be interesting to delineate the contribution of the different stress scenarios to the phenotype we described here. Regarding the human data provided, it would have been nice to further validate the observations from the humanized mouse model and sort and analyze the functional properties of neutrophils from these individuals, but we were limited by the access to fresh blood samples from these patients to isolate and analyze neutrophils.

STAR★METHODS

Detailed methods are provided in the online version of this paper and include the following:

- KEY RESOURCES TABLE
- RESOURCE AVAILABILITY
  - Lead contact
  - Materials availability
  - Data and Code Availability
- EXPERIMENTAL MODEL AND STUDY PARTICIPANT DETAILS
  - Generation of humanized mice reconstituted with control or TET2 mutant primary human HSCs
  - Introduction of TET2 loss of function mutations in primary human HSCs (hHSCs)
  - Clinical Data from human patients
- METHOD DETAILS
  - CyTOF for the immunophenotype of the human hematopoietic system derived from TET2^{Mut} hHSPCs
  - Flow cytometry analysis and cell sorting of human neutrophils
  - Immunostaining and confocal microscopy of NETosis steps
  - Quantification of fluorescence granule signal
  - Adoptive transfer and CFU assay of human neutrophil progenitors
  - LPS in vivo challenge
  - *Candida albicans* in vivo challenge
  - Lung sections and staining
  - Human neutrophils from peripheral blood healthy donors
  - RNA extraction, library preparation and RNA-sequencing of human neutrophils
  - Analysis of control and preleukemic human neutrophils with datasets GSE150021 (neutrophils form human bone marrow) and GSE109467 (neutrophils from mouse)
  - Antimicrobial functional assay

- ○ DNA methylome analysis
- ○ Western blot
- ○ ATACSeq sample preparation, sequencing and analysis
- ○ Time-lapse imaging and NET area quantification assay
- ○ ROS assay
- ○ NET degradation analysis and quantification
- ○ Nuclear morphology quantification
- ○ Scanning electron microscopy
- ○ Transmission electron microscopy
- ● QUANTIFICATION AND STATISTICAL ANALYSIS

SUPPLEMENTAL INFORMATION

Supplemental information can be found online at https://doi.org/10.1016/j.stem.2023.05.004.

ACKNOWLEDGMENTS

We thank all members of Haematopoietic Stem Cell Laboratory for helpful discussions and suggestions and special thanks to Giuseppe D'Agostino for help analyzing the RNA-seq data. We would like to acknowledge the Francis Crick core flow cytometry, advance sequencing, electron microscopy, advanced light microscopy, and biological research facility STPs. This work was supported by the Francis Crick Institute, which receives its core funding from Cancer Research UK (CC1045 and CC1129), the UK Medical Research Council (CC1045 and CC1129), and the Wellcome Trust (CC1045 and CC1129). H.H.E. was funded by the Kay Kendall Leukaemia Foundation (KKL1397). I.V.A. was funded by an EMBO LTF (ALTF 113-2019) and subsequently a Sir Henry Wellcome fellowship (SHWF 222825/Z/21/Z).

AUTHOR CONTRIBUTIONS

H.H.E. conceived and designed the study, carried out experiments, analyzed data, and wrote the manuscript. I.V.A. designed and carried out experiments, analyzed data, and helped write the manuscript. M.G.-A. helped with CyTOF and ATAC-seq analysis, and M.G.A., M.L.S., and R.G. helped with RNA-seq and RRBS analysis. A.S. analyzed NETosis live-imaging experiments. M.P. designed and set up CRISPR protocol of human HSPCs. A.F., A.S., C.M.M., H.W., D.P., Q.W., and L.A.-M. assisted with experiments and study design. M.-C.D. performed electron microscopy experiment and helped with the analysis. T.F. performed and analyzed microscopy experiments. K.G., K.B., D.H.W., and R.G. were involved in obtaining human neutrophils from the clinics and their analysis. V.P. provided advice and technical help on neutrophil studies and interpretation of the data and helpful discussion on the project and help write the paper. D.B. conceived and supervised the study and wrote the manuscript. All authors provided critical feedback on the manuscript pre-submission.

DECLARATION OF INTERESTS

The authors declare no competing interests.

Received: October 28, 2022
Revised: March 27, 2023
Accepted: May 3, 2023
Published: July 6, 2023

REFERENCES

1. Dancey, J.T., Deubelbeiss, K.A., Harker, L.A., and Finch, C.A. (1976). Neutrophil kinetics in man. J. Clin. Invest. *58*, 705–715. https://doi.org/10.1172/JCI108517.
2. Dinh, H.Q., Eggert, T., Meyer, M.A., Zhu, Y.P., Olingy, C.E., Llewellyn, R., Wu, R., and Hedrick, C.C. (2020). Coexpression of CD71 and CD117 identifies an early unipotent neutrophil progenitor population in human bone marrow. Immunity *53*. 319.e6–334.e6. https://doi.org/10.1016/j.immuni.2020.07.017.
3. Zhu, Y.P., Padgett, L., Dinh, H.Q., Marcovecchio, P., Blatchley, A., Wu, R., Ehinger, E., Kim, C., Mikulski, Z., Seumois, G., et al. (2018). Identification of an early unipotent neutrophil progenitor with pro-tumoral activity in mouse and human bone marrow. Cell Rep. *24*. 2329.e8–2341.e8. https://doi.org/10.1016/j.celrep.2018.07.097.
4. Ng, L.G., Ostuni, R., and Hidalgo, A. (2019). Heterogeneity of neutrophils. Nat. Rev. Immunol. *19*, 255–265. https://doi.org/10.1038/s41577-019-0141-8.
5. Evrard, M., Kwok, I.W.H., Chong, S.Z., Teng, K.W.W., Becht, E., Chen, J., Sieow, J.L., Penny, H.L., Ching, G.C., Devi, S., et al. (2018). Developmental analysis of bone marrow neutrophils reveals populations specialized in expansion, trafficking, and effector functions. Immunity *48*. 364.e8–379.e8. https://doi.org/10.1016/j.immuni.2018.02.002.
6. Buscarlet, M., Provost, S., Zada, Y.F., Barhdadi, A., Bourgoin, V., Lépine, G., Mollica, L., Szuber, N., Dubé, M.P., and Busque, L. (2017). DNMT3A and TET2 dominate clonal hematopoiesis and demonstrate benign phenotypes and different genetic predispositions. Blood *130*, 753–762. https://doi.org/10.1182/blood-2017-04-777029.
7. Chan, S.M., and Majeti, R. (2013). Role of DNMT3A, TET2, and IDH1/2 mutations in pre-leukemic stem cells in acute myeloid leukemia. Int. J. Hematol. *98*, 648–657. https://doi.org/10.1007/s12185-013-1407-8.
8. Steensma, D.P., Bejar, R., Jaiswal, S., Lindsley, R.C., Sekeres, M.A., Hasserjian, R.P., and Ebert, B.L. (2015). Clonal hematopoiesis of indeterminate potential and its distinction from myelodysplastic syndromes. Blood *126*, 9–16. https://doi.org/10.1182/blood-2015-03-631747.
9. Watson, C.J., Papula, A.L., Poon, G.Y.P., Wong, W.H., Young, A.L., Druley, T.E., Fisher, D.S., and Blundell, J.R. (2020). The evolutionary dynamics and fitness landscape of clonal hematopoiesis. Science *367*, 1449–1454. https://doi.org/10.1126/science.aay9333.
10. Bowman, R.L., Busque, L., and Levine, R.L. (2018). Clonal hematopoiesis and evolution to hematopoietic malignancies. Cell Stem Cell *22*, 157–170. https://doi.org/10.1016/j.stem.2018.01.011.
11. Florez, M.A., Tran, B.T., Wathan, T.K., DeGregori, J., Pietras, E.M., and King, K.Y. (2022). Clonal hematopoiesis: mutation-specific adaptation to environmental change. Cell Stem Cell *29*, 882–904. https://doi.org/10.1016/j.stem.2022.05.006.
12. Abegunde, S.O., Buckstein, R., Wells, R.A., and Rauh, M.J. (2018). An inflammatory environment containing TNFα favors Tet2-mutant clonal hematopoiesis. Exp. Hematol. *59*, 60–65. https://doi.org/10.1016/j.exphem.2017.11.002.
13. Hormaechea-Agulla, D., Matatall, K.A., Le, D.T., Kain, B., Long, X., Kus, P., Jaksik, R., Challen, G.A., Kimmel, M., and King, K.Y. (2021). Chronic infection drives Dnmt3a-loss-of-function clonal hematopoiesis via IFNγ signaling. Cell Stem Cell *28*. 1428.e6–1442.e6. https://doi.org/10.1016/j.stem.2021.03.002.
14. Jaiswal, S., Natarajan, P., Silver, A.J., Gibson, C.J., Bick, A.G., Shvartz, E., McConkey, M., Gupta, N., Gabriel, S., Ardissino, D., et al. (2017). Clonal hematopoiesis and risk of atherosclerotic cardiovascular disease. N. Engl. J. Med. *377*, 111–121. https://doi.org/10.1056/NEJMoa1701719.
15. Jaiswal, S., Fontanillas, P., Flannick, J., Manning, A., Grauman, P.V., Mar, B.G., Lindsley, R.C., Mermel, C.H., Burtt, N., Chavez, A., et al. (2014). Age-related clonal hematopoiesis associated with adverse outcomes. N. Engl. J. Med. *371*, 2488–2498. https://doi.org/10.1056/NEJMoa1408617.
16. Bolton, K.L., Koh, Y., Foote, M.B., Im, H., Jee, J., Sun, C.H., Safonov, A., Ptashkin, R., Moon, J.H., Lee, J.Y., et al. (2021). Clonal hematopoiesis is associated with risk of severe Covid-19. Nat. Commun. *12*, 5975. https://doi.org/10.1038/s41467-021-26138-6.
17. Miller, P.G., Qiao, D., Rojas-Quintero, J., Honigberg, M.C., Sperling, A.S., Gibson, C.J., Bick, A.G., Niroula, A., McConkey, M.E., Sandoval, B., et al. (2022). Association of clonal hematopoiesis with chronic obstructive pulmonary disease. Blood *139*, 357–368. https://doi.org/10.1182/blood.2021013531.

CellPress
OPEN ACCESS

18. Fuster, J.J., MacLauchlan, S., Zuriaga, M.A., Polackal, M.N., Ostriker, A.C., Chakraborty, R., Wu, C.L., Sano, S., Muralidharan, S., Rius, C., et al. (2017). Clonal hematopoiesis associated withTET2 deficiency accelerates atherosclerosis development in mice. Science *355*, 842–847.

19. Zhang, Q., Zhao, K., Shen, Q., Han, Y., Gu, Y., Li, X., Zhao, D., Liu, Y., Wang, C., Zhang, X., et al. (2015). Tet2 is required to resolve inflammation by recruiting Hdac2 to specifically repress IL-6. Nature *525*, 389–393. https://doi.org/10.1038/nature15252.

20. Meisel, M., Hinterleitner, R., Pacis, A., Chen, L., Earley, Z.M., Mayassi, T., Pierre, J.F., Ernest, J.D., Galipeau, H.J., Thuille, N., et al. (2018). Microbial signals drive pre-leukaemic myeloproliferation in a Tet2-deficient host. Nature *557*, 580.

21. Cull, A.H., Snetsinger, B., Buckstein, R., Wells, R.A., and Rauh, M.J. (2017). Tet2 restrains inflammatory gene expression in macrophages. Exp. Hematol. *55*. 56.e13–70.e13. https://doi.org/10.1016/j.exphem.2017.08.001.

22. Döring, Y., Drechsler, M., Soehnlein, O., and Weber, C. (2015). Neutrophils in atherosclerosis: from mice to man. Arterioscler. Thromb. Vasc. Biol. *35*, 288–295. https://doi.org/10.1161/ATVBAHA.114.303564.

23. Döring, Y., Libby, P., and Soehnlein, O. (2020). Neutrophil extracellular traps participate in cardiovascular diseases: recent experimental and clinical insights. Circ. Res. *126*, 1228–1241. https://doi.org/10.1161/CIRCRESAHA.120.315931.

24. Warnatsch, A., Ioannou, M., Wang, Q., and Papayannopoulos, V. (2016). Neutrophil extracellular traps license macrophages and Th17 cells for cytokine production in atherosclerosis*349*, pp. 316–320. https://doi.org/10.1126/science.aaa8064.Neutrophil.

25. Grabcanovic-Musija, F., Obermayer, A., Stoiber, W., Krautgartner, W.D., Steinbacher, P., Winterberg, N., Bathke, A.C., Klappacher, M., and Studnicka, M. (2015). Neutrophil extracellular trap (NET) formation characterises stable and exacerbated COPD and correlates with airflow limitation. Respir. Res. *16*, 59. https://doi.org/10.1186/s12931-015-0221-7.

26. Papayannopoulos, V., Staab, D., and Zychlinsky, A. (2011). Neutrophil elastase enhances sputum solubilization in cystic fibrosis patients receiving DNase therapy. PLoS One *6*. e28526. https://doi.org/10.1371/journal.pone.0028526.

27. Khandpur, R., Carmona-Rivera, C., Vivekanandan-Giri, A., Gizinski, A., Yalavarthi, S., Knight, J.S., Friday, S., Li, S., Patel, R.M., Subramanian, V., et al. (2013). NETs are a source of citrullinated autoantigens and stimulate inflammatory responses in rheumatoid arthritis. Sci. Transl. Med. *5*. 178ra40. https://doi.org/10.1126/scitranslmed.3005580.

28. Metzler, K.D., Fuchs, T.A., Nauseef, W.M., Reumaux, D., Roesler, J., Schulze, I., Wahn, V., Papayannopoulos, V., and Zychlinsky, A. (2011). Myeloperoxidase is required for neutrophil extracellular trap formation: implications for innate immunity. Blood *117*, 953–959. https://doi.org/10.1182/blood-2010-06-290171.

29. Jiménez-Alcázar, M., Rangaswamy, C., Panda, R., Bitterling, J., Simsek, Y.J., Long, A.T., Bilyy, R., Krenn, V., Renné, C., Renné, T., et al. (2017). Jiménez-Alcázar, M., Rangaswamy, C., Panda, R., Bitterling, J., Simsek, Y.J., Long, A.T., Bilyy, R., Krenn, V., Renné, C., Renné, T., et al.. (2017). Host DNases prevent vascular occlusion by neutrophil extracellular traps. Science 358, 1202–1206. 10.1126/science.aam8897.

30. Fuchs, T.A., Brill, A., Duerschmied, D., Schatzberg, D., Monestier, M., Myers, D.D., Wrobleski, S.K., Wakefield, T.W., Hartwig, J.H., and Wagner, D.D. (2010). Extracellular DNA traps promote thrombosis. Proc. Natl. Acad. Sci. USA *107*, 15880–15885. https://doi.org/10.1073/pnas.1005743107.

31. Wolach, O., Sellar, R.S., Martinod, K., Cherpokova, D., McConkey, M., Chappell, R.J., Silver, A.J., Adams, D., Castellano, C.A., Schneider, R.K., et al. (2018). Increased neutrophil extracellular trap formation promotes thrombosis in myeloproliferative neoplasms. Sci. Transl. Med. *10*, 1–11. https://doi.org/10.1126/scitranslmed.aan8292.

32. Cook, E.K., Luo, M., and Rauh, M.J. (2020). Clonal hematopoiesis and inflammation: partners in leukemogenesis and comorbidity. Exp. Hematol. *83*, 85–94. https://doi.org/10.1016/j.exphem.2020.01.011.

33. Hidalgo, A., and Tall, A.R. (2019). Leducq transatlantic network on clonal hematopoiesis and atherosclerosis. Circ. Res. *124*, 481–483. https://doi.org/10.1161/CIRCRESAHA.119.314677.

34. Wunderlich, M., Chou, F.S., Sexton, C., Presicce, P., Chougnet, C.A., Aliberti, J., and Mulloy, J.C. (2018). Improved multilineage human hematopoietic reconstitution and function in NSGS mice. PLoS One *13*. e0209034. https://doi.org/10.1371/journal.pone.0209034.

35. McIntosh, B.E., Brown, M.E., Duffin, B.M., Maufort, J.P., Vereide, D.T., Slukvin, I.I., and Thomson, J.A. (2015). Nonirradiated NOD,B6.SCID Il2rγ-/- Kit(W41/W41) (NBSGW) mice support multilineage engraftment of human hematopoietic cells. Stem Cell Rep. *4*, 171–180. https://doi.org/10.1016/j.stemcr.2014.12.005.

36. Parmley, R.T., Rice, W.G., Kinkade, J.M., Gilbert, C., and Barton, J.C. (1987). Peroxidase-containing microgranules in human neutrophils: physical, morphological, cytochemical, and secretory properties. Blood *70*, 1630–1638. https://doi.org/10.1182/blood.v70.5.1630.1630.

37. Lévy, Y., Wiedemann, A., Hejblum, B.P., Durand, M., Lefebvre, C., Surénaud, M., Lacabaratz, C., Perreau, M., Foucat, E., Déchenaud, M., et al. (2021). CD177, a specific marker of neutrophil activation, is associated with coronavirus disease 2019 severity and death. iScience *24*. 102711. https://doi.org/10.1016/j.isci.2021.102711.

38. Källquist, L., Hansson, M., Persson, A.M., Janssen, H., Calafat, J., Tapper, H., and Olsson, I. (2008). The tetraspanin CD63 is involved in granule targeting of neutrophil elastase. Blood *112*, 3444–3454. https://doi.org/10.1182/blood-2007-10-116285.

39. Lizcano, A., Secundino, I., Döhrmann, S., Corriden, R., Rohena, C., Diaz, S., Ghosh, P., Deng, L., Nizet, V., and Varki, A. (2017). Erythrocyte sialoglycoproteins engage Siglec-9 on neutrophils to suppress activation. Blood *129*, 3100–3110. https://doi.org/10.1182/blood-2016-11-751636.

40. Montaldo, E., Lusito, E., Bianchessi, V., Caronni, N., Scala, S., Basso-Ricci, L., Cantaffa, C., Masserdotti, A., Barilaro, M., Barresi, S., et al. (2022). Cellular and transcriptional dynamics of human neutrophils at steady state and upon stress. Nat. Immunol. *23*, 1470–1483. https://doi.org/10.1038/s41590-022-01311-1.

41. Perez, C., Botta, C., Zabaleta, A., Puig, N., Cedena, M.T., Goicoechea, I., Alameda, D., San José-Eneriz, E.S., Merino, J., Rodríguez-Otero, P., et al. (2020). Immunogenomic identification and characterization of granulocytic myeloid-derived suppressor cells in multiple myeloma. Blood *136*, 199–209. https://doi.org/10.1182/blood.2019004537.

42. Wu, X., and Zhang, Y. (2017). TET-mediated active DNA demethylation: mechanism, function and beyond. Nat. Rev. Genet. *18*, 517–534. https://doi.org/10.1038/nrg.2017.33.

43. Tulstrup, M., Soerensen, M., Hansen, J.W., Gillberg, L., Needhamsen, M., Kaastrup, K., Helin, K., Christensen, K., Weischenfeldt, J., and Grønbæk, K. (2021). TET2 mutations are associated with hypermethylation at key regulatory enhancers in normal and malignant hematopoiesis. Nat. Commun. *12*, 6061. https://doi.org/10.1038/s41467-021-26093-2.

44. Buitrago, D., Labrador, M., Arcon, J.P., Lema, R., Flores, O., Esteve-Codina, A., Blanc, J., Villegas, N., Bellido, D., Gut, M., et al. (2021). Impact of DNA methylation on 3D genome structure. Nat. Commun. *12*, 3243. https://doi.org/10.1038/s41467-021-23142-8.

45. Khoyratty, T.E., Ai, Z., Ballesteros, I., Eames, H.L., Mathie, S., Martín-Salamanca, S., Wang, L., Hemmings, A., Willemsen, N., von Werz, V., et al. (2021). Distinct transcription factor networks control neutrophil-driven inflammation. Nat. Immunol. *22*, 1093–1106. https://doi.org/10.1038/s41590-021-00968-4.

46. Grassi, L., Pourfarzad, F., Ullrich, S., Merkel, A., Were, F., Carrillo-de-Santa-Pau, E., Yi, G., Hiemstra, I.H., Tool, A.T.J., Mul, E., et al. (2018). Dynamics of transcription regulation in human bone marrow myeloid differentiation to mature blood neutrophils. Cell Rep. *24*, 2784–2794. https://doi.org/10.1016/j.celrep.2018.08.018.

47. Kunimoto, H., Fukuchi, Y., Sakurai, M., Sadahira, K., Ikeda, Y., Okamoto, S., and Nakajima, H. (2012). Tet2 disruption leads to enhanced self-renewal and altered differentiation of fetal liver hematopoietic stem cells. Sci. Rep. *2*, 273. https://doi.org/10.1038/srep00273.

48. Nakauchi, Y., Azizi, A., Thomas, D., Corces, M.R., Reinisch, A., Sharma, R., Cruz Hernandez, D., Köhnke, T., Karigane, D., Fan, A., et al. (2022). The cell type–specific 5hmC landscape and dynamics of healthy human hematopoiesis and TET2-mutant preleukemia. Blood Cancer Discov. *3*, 346–367. https://doi.org/10.1158/2643-3230.BCD-21-0143.
49. Moran-Crusio, K., Reavie, L., Shih, A., Abdel-Wahab, O., Ndiaye-Lobry, D., Lobry, C., Figueroa, M.E., Vasanthakumar, A., Patel, J., Zhao, X., et al. (2011). Tet2 loss leads to increased hematopoietic stem cell self-renewal and myeloid transformation. Cancer Cell *20*, 11–24. https://doi.org/10.1016/j.ccr.2011.06.001.
50. Papayannopoulos, V., Metzler, K.D., Hakkim, A., and Zychlinsky, A. (2010). Neutrophil elastase and myeloperoxidase regulate the formation of neutrophil extracellular traps. J. Cell Biol. *191*, 677–691. https://doi.org/10.1083/jcb.201006052.
51. Urban, C.F., Reichard, U., Brinkmann, V., and Zychlinsky, A. (2006). Neutrophil extracellular traps capture and kill Candida albicans yeast and hyphal forms. Cell. Microbiol. *8*, 668–676. https://doi.org/10.1111/j.1462-5822.2005.00659.x.
52. Branzk, N., Lubojemska, A., Hardison, S.E., Wang, Q., Gutierrez, M.G., Brown, G.D., and Papayannopoulos, V. (2014). Neutrophils sense microbe size and selectively release neutrophil extracellular traps in response to large pathogens. Nat. Immunol. *15*, 1017–1025. https://doi.org/10.1038/ni.2987.
53. Urban, C.F., Ermert, D., Schmid, M., Abu-Abed, U., Goosmann, C., Nacken, W., Brinkmann, V., Jungblut, P.R., and Zychlinsky, A. (2009). Neutrophil extracellular traps contain calprotectin, a cytosolic protein complex involved in host defense against Candida albicans. PLoS Pathog. *5*. e1000639. https://doi.org/10.1371/journal.ppat.1000639.
54. Papayannopoulos, V. (2018). Neutrophil extracellular traps in immunity and disease. Nat. Rev. Immunol. *18*, 134–147. https://doi.org/10.1038/nri.2017.105.
55. Ko, M., Bandukwala, H.S., An, J., Lamperti, E.D., Thompson, E.C., Hastie, R., Tsangaratou, A., Rajewsky, K., Koralov, S.B., and Rao, A. (2011). Ten-eleven-translocation 2 (TET2) negatively regulates homeostasis and differentiation of hematopoietic stem cells in mice. Proc. Natl. Acad. Sci. USA *108*, 14566–14571. https://doi.org/10.1073/pnas.1112317108.
56. Challen, G.A., and Goodell, M.A. (2020). Clonal hematopoiesis: mechanisms driving dominance of stem cell clones. Blood *136*, 1590–1598. https://doi.org/10.1182/blood.2020006510.
57. Caiado, F., Pietras, E.M., and Manz, M.G. (2021). Inflammation as a regulator of hematopoietic stem cell function in disease, aging, and clonal selection. J. Exp. Med. *218*, 1–19. https://doi.org/10.1084/jem.20201541.
58. Mian, S.A., Anjos-Afonso, F., and Bonnet, D. (2021). Advances in human immune system mouse models for studying human hematopoiesis and cancer immunotherapy. Front. Immunol. *11*. 619236. https://doi.org/10.3389/fimmu.2020.619236.
59. Abarrategi, A., Mian, S.A., Passaro, D., Rouault-Pierre, K., Grey, W., and Bonnet, D. (2018). Modeling the human Bone Marrow niche in mice: From host bone marrow engraftment to bioengineering approaches. J Exp Med. *215*, 729–743. https://doi.org/10.1084/jem.20172139.
60. Minoda, Y., Virshup, I., Leal Rojas, I.L., Haigh, O., Wong, Y., Miles, J.J., Wells, C.A., and Radford, K.J. (2017). Human CD141+ dendritic cell and CD1c+ dendritic cell undergo concordant early genetic programming after activation in humanized mice in vivo. Front. Immunol. *8*, 1419. https://doi.org/10.3389/fimmu.2017.01419.
61. Evren, E., Ringqvist, E., Tripathi, K.P., Sleiers, N., Rives, I.C., Alisjahbana, A., Gao, Y., Sarhan, D., Halle, T., Sorini, C., et al. (2021). Distinct developmental pathways from blood monocytes generate human lung macrophage diversity. Immunity *54*. 259.e7–275.e7. https://doi.org/10.1016/j.immuni.2020.12.003.
62. Rao, S., Yao, Y., Soares de Brito, J., Yao, Q., Shen, A.H., Watkinson, R.E., Kennedy, A.L., Coyne, S., Ren, C., Zeng, J., et al. (2021). Dissecting ELANE neutropenia pathogenicity by human HSC gene editing. Cell Stem Cell *28*. 833.e5–845.e5. https://doi.org/10.1016/j.stem.2020.12.015.
63. Porwit, A., Van De Loosdrecht, A.A., Bettelheim, P., Brodersen, L.E., Burbury, K., Cremers, E., Della Porta, M.G., Ireland, R., Johansson, U., Matarraz, S., et al. (2014). Revisiting guidelines for integration of flow cytometry results in the WHO classification of myelodysplastic syndromes – proposal from the International/European LeukemiaNet Working Group for Flow Cytometry in MDS. Leukemia*28*, pp. 1793–1798. https://doi.org/10.1038/leu.2014.191.
64. Bento, L.C., Correia, R.P., Pitangueiras Mangueira, C.L., De Souza Barroso, R., Rocha, F.A., Bacal, N.S., and Marti, L.C. (2017). The use of flow cytometry in myelodysplastic syndromes: a review. Front. Oncol. *7*, 270. https://doi.org/10.3389/fonc.2017.00270.
65. Dawoud, A.A.Z., Tapper, W.J., and Cross, N.C.P. (2020). Clonal myelopoiesis in the UK Biobank cohort: ASXL1 mutations are strongly associated with smoking. Leukemia *34*, 2660–2672. https://doi.org/10.1038/s41375-020-0896-8.
66. Kar, S.P., Quiros, P.M., Gu, M., Jiang, T., Mitchell, J., Langdon, R., Iyer, V., Barcena, C., Vijayabaskar, M.S., Fabre, M.A., et al. (2022). Genome-wide analyses of 200,453 individuals yield new insights into the causes and consequences of clonal hematopoiesis. Nat. Genet. *54*, 1155–1166. https://doi.org/10.1038/s41588-022-01121-z.
67. Banks, K.M., Lan, Y., and Evans, T. (2021). Tet proteins regulate neutrophil granulation in zebrafish through demethylation of socs3b mRNA. Cell Rep. *34*. 108632. https://doi.org/10.1016/j.celrep.2020.108632.
68. Ostrander, E.L., Kramer, A.C., Mallaney, C., Celik, H., Koh, W.K., Fairchild, J., Haussler, E., Zhang, C.R.C., and Challen, G.A. (2020). Divergent effects of Dnmt3a and Tet2 mutations on hematopoietic progenitor cell fitness. Stem Cell Rep. *14*, 551–560. https://doi.org/10.1016/j.stemcr.2020.02.011.
69. Ito, K., Lee, J., Chrysanthou, S., Zhao, Y., Josephs, K., Sato, H., Teruya-Feldstein, J., Zheng, D., Dawlaty, M.M., and Ito, K. (2019). Non-catalytic roles of Tet2 are essential to regulate hematopoietic stem and progenitor cell homeostasis. Cell Rep. *28*. 2480.e4–2490.e4. https://doi.org/10.1016/j.celrep.2019.07.094.
70. Ballesteros, I., Rubio-Ponce, A., Genua, M., Lusito, E., Kwok, I., Fernández-Calvo, G., Khoyratty, T.E., van Grinsven, E., González-Hernández, S., Nicolás-Ávila, J.Á., et al. (2020). Co-option of neutrophil fates by tissue environments. Cell *183*. 1282.e18–1297.e18. https://doi.org/10.1016/j.cell.2020.10.003.
71. Tsourouktsoglou, T.D., Warnatsch, A., Ioannou, M., Hoving, D., Wang, Q., and Papayannopoulos, V. (2020). Histones, DNA, and citrullination promote neutrophil extracellular trap inflammation by regulating the localization and activation of TLR4. Cell Rep. *31*. 107602. https://doi.org/10.1016/j.celrep.2020.107602.
72. Hoenderdos, K., and Condliffe, A. (2013). The neutrophil in chronic obstructive pulmonary disease? Am. J. Respir. Cell Mol. Biol. *48*, 531–539. https://doi.org/10.1165/rcmb.2012-0492TR.
73. Dicker, A.J., Crichton, M.L., Pumphrey, E.G., Cassidy, A.J., Suarez-Cuartin, G., Sibila, O., Furrie, E., Fong, C.J., Ibrahim, W., Brady, G., et al. (2018). Neutrophil extracellular traps are associated with disease severity and microbiota diversity in patients with chronic obstructive pulmonary disease. J. Allergy Clin. Immunol. *141*, 117–127. https://doi.org/10.1016/j.jaci.2017.04.022.
74. Cimmino, L., Dolgalev, I., Wang, Y., Yoshimi, A., Martin, G.H., Wang, J., Ng, V., Xia, B., Witkowski, M.T., Mitchell-Flack, M., et al. (2017). Restoration of TET2 function blocks aberrant self-renewal and leukemia progression. Cell *170*. 1079.e20–1095.e20. https://doi.org/10.1016/j.cell.2017.07.032.
75. Huerga Encabo, H., Ulferts, R., Sharma, A., Beale, R., and Bonnet, D. (2021). Infecting human hematopoietic stem and progenitor cells with SARS-CoV-2. Star Protoc. *2*. 100903. https://doi.org/10.1016/j.xpro.2021.100903.
76. Lindeboom, R.G.H., Supek, F., and Lehner, B. (2016). The rules and impact of nonsense-mediated mRNA decay in human cancers. Nat. Genet. *48*, 1112–1118. https://doi.org/10.1038/ng.3664.
77. Hu, L., Li, Z., Cheng, J., Rao, Q., Gong, W., Liu, M., Shi, Y.G., Zhu, J., Wang, P., and Xu, Y. (2013). Crystal structure of TET2-DNA complex: insight into

TET-mediated 5mC oxidation. Cell *155*, 1545–1555. https://doi.org/10.1016/j.cell.2013.11.020.

78. Gupta, R., Gant, V.A., Williams, B., and Enver, T. (2020). Increased Complement Receptor-3 levels in monocytes and granulocytes distinguish COVID-19 patients with pneumonia from those with mild symptoms. Int. J. Infect. Dis. *99*, 381–385.

79. Bagwell, C.B., Inokuma, M., Hunsberger, B., Herbert, D., Bray, C., Hill, B., Stelzer, G., Li, S., Kollipara, A., Ornatsky, O., et al. (2020). Automated data cleanup for mass cytometry. Cytometry A *97*, 184–198. https://doi.org/10.1002/cyto.a.23926.

80. Chevrier, S., Crowell, H.L., Zanotelli, V.R.T., Engler, S., Robinson, M.D., and Bodenmiller, B. (2018). Compensation of signal spillover in suspension and imaging mass cytometry. Cell Syst. *6*. 612.e5–620.e5. https://doi.org/10.1016/j.cels.2018.02.010.

81. Bankhead, P., Loughrey, M.B., Fernández, J.A., Dombrowski, Y., McArt, D.G., Dunne, P.D., McQuaid, S., Gray, R.T., Murray, L.J., Coleman, H.G., et al. (2017). QuPath: open source software for digital pathology image analysis. Sci. Rep. *7*. 16878. https://doi.org/10.1038/s41598-017-17204-5.

82. Corces, M.R., Trevino, A.E., Hamilton, E.G., Greenside, P.G., Sinnott-Armstrong, N.A., Vesuna, S., Satpathy, A.T., Rubin, A.J., Montine, K.S., Wu, B., et al. (2017). An improved ATAC-seq protocol reduces background and enables interrogation of frozen tissues. Nat. Methods *14*, 959–962. https://doi.org/10.1038/nmeth.4396.

83. Berg, S., Kutra, D., Kroeger, T., Straehle, C.N., Kausler, B.X., Haubold, C., Schiegg, M., Ales, J., Beier, T., Rudy, M., et al. (2019). Ilastik: interactive machine learning for (bio)image analysis. Nat. Methods *16*, 1226–1232. https://doi.org/10.1038/s41592-019-0582-9.

84. Stirling, D.R., Swain-Bowden, M.J., Lucas, A.M., Carpenter, A.E., Cimini, B.A., and Goodman, A. (2021). CellProfiler 4: improvements in speed, utility and usability. BMC Bioinformatics *22*, 433. https://doi.org/10.1186/s12859-021-04344-9.

85. Meechan, K., Guan, W., Riedinger, A., Stankova, V., Yoshimura, A., Pipitone, R., Milberger, A., Schaar, H., Romero-Brey, I., Templin, R., et al. (2022). Crosshair, semi-automated targeting for electron microscopy with a motorised ultramicrotome. eLife *11*, 1–28. https://doi.org/10.7554/eLife.80899.

STAR★METHODS

KEY RESOURCES TABLE

REAGENT or RESOURCE	SOURCE	IDENTIFIER
Antibodies		
anti-human CD45-PerCP-Cy5.5	BD Bioscience	AB_2744405
anti-human CD16-APC-Cy7	Biolegend	AB_314217
anti-human CD14-PE-Cy7	Invitrogen	AB_1582276
anti-human CD66b-APC	Biolegend	AB_2566606
anti-human CD15-BV786	BD Bioscience	AB_2740635
anti-human CD71-FITC	Biolegend	AB_1236432
anti-human CD49d-PE	Biolegend	AB_314429
anti-mouse CD45-BV421	BD Bioscience	AB_2651151
CyTOF antibodies listed in Table S1	Fluidigm/Crick STP	N/A
anti-MPO	R&D Systems	AB_2250866
anti-Neutrophil elastase NP47	GeneTex	AB_383332
anti-PR3	Abcam	AB_2936820
anti-human CD45-FITC HI30	Biolegend	AB_10852703
Anti-mouse Ly6G-Alexa647 1A8	Biolegend	AB_1134162
Rabbit Anti-Candida albicans Antibody	Acris Antibodies	AB_973716
Bacterial and virus strains		
Candida albicans	clinical isolate SC5314	N/A
Biological samples		
Human umbilical cord blood	Royal London Hospital (London, U.K.)	N/A
Human peripheral blood	The Francis Crick Institute (London, U.K.)	N/A
Chemicals, peptides, and recombinant proteins		
DAPI	BD Biosciences	564907
Cas9	IDT	1081060
Zymosan bioparticles from *S.cerevisae*	ThemoFisher	Z23373
PMA (Phorbol 12-myristate 13-acetate)	Sigma	P8139
Hoechst 33342	Cell Signalling	4082
Luminol	Sigma	A8511
Sytox green	Invitrogen	S7020
Peroxidase from horseradish	Sigma	P6140-10KU
Deoxyribonuclease I from bovine pancreas	Sigma	D5025
Critical commercial assays		
Human/mouse chimera isolation kit	StemCell technologies	19849
Human Progenitor Cell Enrichment Kit	StemCell technologies	17936
Methylcellulose for CFU assay	StemCell technologies	M3434
Deposited data		
Original raw and analyzed data	This paper	GEO: GSE213771
RNASeq: Neutrophils from human bone marrow	Perez et al.[41]	GEO: GSE150021
RNASeq: Neutrophils from mouse bone marrow	Evrard et al.[5]	GEO: GSE109467
RNASeq: Neutrophil development from TET2-mutant hHSCs	This paper	GEO: GSE228077
TEM images of control and TET2-mutant neutrophils	This paper	BioImage Archive: S-BIAD650

(Continued on next page)

Continued

REAGENT or RESOURCE	SOURCE	IDENTIFIER
Videos from neutrophils derived from TET2Mut HSCs undergoing NETosis	This paper	https://data.mendeley.com/datasets/g673bgpwxc/1
Experimental models: Organisms/strains		
Mouse: NSG-S cKit$^{w41/w41}$	This paper	N/A
Oligonucleotides		
gRNA for TET2 editing	This paper: Table S1	N/A
Primers for TET2 sequencing	This paper: Table S1	N/A
Software and algorithms		
FACSDiva 6.2	BD Biosciences	N/A
FCS Express 7	DeNovo	N/A
Imagej2 (v2.3.0/1.53f)	Courtesy of Spike Walker	N/A
Prism 9 Version 9.1.2 (225)	GraphPad	N/A
Other		
Nikon eclipse T2 inverted wide-field microscope	Imaging core facility	N/A
Leica TCS SP8 inverted confocal microscope	Imaging core facility	N/A

RESOURCE AVAILABILITY

Lead contact

Further information and requests for resources and reagents should be directed to and will be fulfilled by the lead contact, Dominique Bonnet (Dominique.bonnet@crick.ac.uk).

Materials availability

All biological materials used in this study are available from the lead contact upon request or from commercial sources. This study did not generate new unique reagents.

Data and Code Availability

RNA-sequencing, reduced-representation bisulfite sequencing and ATAC-sequencing data have been deposited at GEO and are publicly available as of the date of publication. Accession numbers are listed in the key resources table. Electron microscopy data have been deposited at BioImage Archive and are publicly available as of the date of publication. Videos from neutrophils derived from TET2Mut HSCs undergoing NETosis have been deposited in Mendeley. Accession numbers are listed in the key resources table. This paper analyzes existing, publicly available data. These accession numbers for the datasets are listed in the key resources table.

This paper does not report original code.

Any additional information required to reanalyze the data reported in this paper is available from the lead contact upon request.

EXPERIMENTAL MODEL AND STUDY PARTICIPANT DETAILS

Generation of humanized mice reconstituted with control or TET2 mutant primary human HSCs

NOD.Cg-Kit$^{W\text{-}41J}$ Prkdc^{scid} Il2rg^{tm1} and NSG-SGM3 (NOD. Prkdc^{scid} Il2rg^{tm1} Tg(CMV-IL3, CSF2, KITLG) mice were originally obtained from The Jackson Laboratory, bred for 7 generations together to produce the NSG-S cKit$^{w41/w41}$ used in this experiment. Mice are bred in isolators with aseptic standard operating procedures in the Biological Research Facility of The Francis Crick Institute. Once weaned, mice were kept in ventilated cages. All animal experiments were performed under the U.K Home Office project license (70/8904) in accordance with The Francis Crick Institute animal ethics committee guidance. Males and females NSG-S cKit$^{w41/w41}$ mice age between 8 – 12 weeks, were used in these experiments. These mice were injected with hHSC (10,000-20,000 Lin-CD34^{+}CD38^{-} cells/mouse) via intravenous administration. Engraftment and mutation efficiency within the reconstituted human hematopoietic system reconstituted was analyzed for each mouse by bone marrow aspiration at 8-10 weeks post injection and the mice were sacrificed between 12-14 weeks post-transplantation.

Introduction of TET2 loss of function mutations in primary human HSCs (hHSCs)

Umbilical Cord Blood (UCB) samples were obtained from full term donors after informed consent at the Royal London Hospital (London, U.K.) and under approval by the East London Ethical Research committee. Mononuclear cells (MNCs) were isolated by density

centrifugation using Ficoll-Paque (GE 67 Healthcare). MNCs were depleted for lineage marker positive cells using an EasySep Human Progenitor Cell Enrichment Kit (Stem Cell Technologies) and HSPCs isolated as described in detail previously.[75] After sorting human HSCs ($CD34^{+}CD38^{-}$) were seeded in StemSpanSFEM (Stem Cell Technologies) supplemented with 100 ng/mL rhFLT-3L, 100 ng/mL rhSCF and 100 ng/mL rhTPO. After 48 hours cells were collected for CRISPR editing using the NEON Transfection system (Thermofisher). Cells were counted 48 hours after and used for xenograft and CRISPR efficiency was analyzed by next generation sequencing. Small guide RNA targeting TET2 and primers to assess CRISPR efficiency are listed in Table S1. All experiments analyzed included mice engrafted with human HSPCs with >80% efficiency in human reconstitution. We have validated that in all our experiments CRISPR efficiency was >90% of TET2 mutations that cause premature stop in exon 11 (From Y1576 the insertion of an A base pair in the DNA sequence leads to 7aa and a stop codon) and is therefore likely to be degraded via the NMD pathway or generate a truncated protein.[76] The truncated protein lacks key residues in the catalytic domain required for interaction with methyl cytosine (1902 and 1094), the stabilization of the substrate 2-oxoglutarate (H1881, S1898, R1896) and Fe ion chelation (H1881).[77]

Clinical Data from human patients

For "UCLH cohort" data: median side scatter (SSC) of mature granulocytes in adult human bone marrow was measured by retrospectively re-analysing archived flow cytometry data files from patients with suspected blood disorders who had liquid BM biopsies for routine diagnostic purposes in the Specialist Integrated Haematological Malignancy Diagnostic Service (SIHMDS) at University College London Hospitals (UCLH, London UK). The UCLH SIHMDS laboratory is fully clinically accredited by The United Kingdom Accreditation Service (UKAS). Specimens were collected, processed, and analyzed according to a standard diagnostic algorithm used for all patients, which set out below:

(a) 3-4ml BM was aspirated from the iliac crest and placed into sterile containers coated with lyophilised EDTA. Samples were processed within 18 hours of collection.
(b) 0.2ml BM was prepared for flow cytometry using the TQ-Prep lysis system (Beckman Coulter) as previously described https://doi.org/10.1016/j.ijid.2020.08.004.[78] Antibody staining was with a Duraclone 'General Orientation' lyophilised antibody panel (cat. no. B93642, Beckman Coulter). This panel is in routine clinical use at UCLH but is bespoke and not commercially available. Details of the antibodies are provided in Table S7). A minimum of 50000 events were collected with a Navios EX 10 colour flow cytometer (Beckman Coulter). Data analysis was with Kaluza (Beckman Coulter) and the gating strategy for identifying granulocytes and assessing their SSC and percentage is shown in Figure S8C.
(c) Targeted NGS of whole BM DNA was performed using the Archer VariantPlex Myeloid panel (IDT) with a detection sensitivity of 2.5%. This panel analyses 75 gene loci including TET2
(d) Chromosomal microarray (CMA) was performed with 60K oligonucleotide arrays (Agilent), and analyzed with Agilent CytoGenomics v2.11.0 at an average resolution of 50Kb. Sample DNA was hybridized against commercially available same-sex control DNA (Promega).

No sample underwent additional laboratory investigations for the purposes of this study. All data were anonymized in accordance with local institutional guidelines. 22 patients were identified with isolated truncation mutations of TET2 but who had no other mutations in either the NGS or CMA studies, and their archived flow cytometry datafiles were selected for reanalysis. Their characteristics and hematological diagnoses are given in Table S7.

For "Manchester cohort" data: neutrophils were isolated by immunomagnetic negative isolation from 2-5 ml of fresh peripheral blood taken from either healthy volunteers or CMML patients using EasySep Direct neutrophil isolation kit (Stem cell Technology Cat No: 19666). Peripheral blood samples from CMML patients were obtained from the Manchester Cancer Research Centre Tissue Biobank (initiated with the approval of South Manchester Research Ethics Committee). Peripheral blood samples from healthy volunteers were obtained with the approval from HRA (REC:19/LO/0564). Isolated neutrophils are stained with antibodies (dilutions 1:100) CD14-FITC, CD16-APC-Cy7, CD66b-APC, and CD11b-PE and analysed on LSRII (BD Biosciences). Their characteristics are given in Table S7.

METHOD DETAILS

CyTOF for the immunophenotype of the human hematopoietic system derived from $TET2^{Mut}$ hHSPCs

Where available, antibodies were purchased pre-conjugated with metals from Fluidigm. The remaining antibodies were purchased from other manufacturers and custom conjugated by staff in the Flow Cytometry core facility at the Francis Crick Institute using Maxpar Antibody Labelling Kits (Fluidigm). The panel of antibodies used is listed in Figure S1 and Table S1.

Sorted cells were washed with phosphate-buffered saline without calcium or magnesium (specified PBS) (Gibco). Viability staining was performed by incubation with 1μM cisplatin intercalator (Fluidigm) for 5 minutes at room temperature. Cells were then washed twice with cell staining medium (CSM, specified PBS with 5mg/mL protease-free bovine serum albumin (Sigma-Aldrich)) and incubated with Human TruStain FcX Fc receptor blocking solution (Biolegend) diluted 1:2 in specified PBS for 10 minutes at room temperature. Cell surface staining was performed with a premixed cocktail of 34 metal-conjugated antibodies (Table S1) for 30 minutes at room temperature. Cells were again washed twice with CSM and incubated in fixation/permeabilization solution (ThermoFisher) for 45 minutes at 4°C. Two washes were performed with permeabilization buffer (ThermoFisher), before intracellular staining with a

premixed cocktail of 2 metal-conjugated antibodies (Table S1) for 30 minutes at 4°C. Cells were washed with permeabilization buffer and fixed in 1.6% paraformaldehyde (PFA) (ThermoFisher) for 10 minutes at room temperature prior to incubation in 0.25μM iridium intercalator (Fluidigm) in 1.6% PFA overnight at 4°C. Stained cells were washed once with Maxpar Cell Staining Buffer (Fluidigm) and twice with Cell Acquisition Solution Plus (CAS+) (Fluidigm). These cells were resuspended in CAS^{+} and EQ6 beads (Fluidigm) were added prior to acquisition in a CyTOF XT mass cytometer (Fluidigm) in the Flow Cytometry core facility at the Francis Crick Institute. To allow generation of a spillover matrix for compensation during data analysis, thirty-six aliquots of OneComp eBeads Compensation Beads (ThermoFisher) were each stained with a single metal-conjugated antibody from the panel (Table S1) and acquired separately in the CyTOF XT mass cytometer. CyTOF XT files were extracted as.fcs files. Analysis was performed using FlowJo (BD Biosciences) for clean-up/gating and the CATALYST R package, for normalization, removal of the EQ6 bead signal, generation of the spillover matrix and compensation, as previously described for subsequent analysis.[79,80] The resulting normalized.fcs files were uploaded to the R environment using the flowCore package and a Seurat object was created using the Seurat package under the R environment. Data was scaled and variable features were found and PCA and UMAP dimensionality reductions were performed. Sub-setting of cell subsets 0, 3, 5 and 15, identified as granulocytes ($CD66b^{+}CD14^{-}$), was performed and the UMAP was re-run to obtain optimal number of clusters. For heatmap and violin visualizations ggplot package and VlnPlot function were used. Next, a pseudo-time analysis was developed using the Monocle package on R. For the pseudo-time analysis, a sub-setting of 500 cells per cluster was done and analyzed.

Flow cytometry analysis and cell sorting of human neutrophils

All experiments were analyzed at the Flow Cytometry core facility of The Francis Crick Institute using the LSR FORTESSA (BD Biosciences) equipped with a 488-nm laser, a 561-nm laser, a 633-nm laser, and a 405-nm laser. For sorting, cell suspensions were filtered through a 35-μm nylon mesh (Falcon, Cat# 352235) and sorted in a BD FACS FUSION cell sorter equipped with 488-nm, 561-nm, 633-nm, and 405-nm lasers. The antibodies used to sort, and phenotype human neutrophils were: CD45-PerCP-Cy5.5 (clone HI30, BD Bioscience), CD16-APC-Cy7 (clone 3G8, Biolegend), CD14-PE-Cy7 (clone 61D3, Invitrogen), CD66b-APC (clone G10F5, Biolegend), CD15-BV786 (clone W6D3, BD Bioscience), CD71-FITC (clone CY1G4, Biolegend, CD49d-PE (clone 9F10, Biolegend). Exclusion of dead cells was done by staining with the fluorescent dye DAPI (1 μg/ml; BD Biosciences, Cat# 564907) and exclusion of remaining mouse hematopoietic cells by including anti-mouse CD45-BV410 (clone 30F11, BD Bioscience) and gating out the positive cells. All experiments were analyzed with FACSDiva 6.2 (BD Biosciences) and FCS Express 7 software.

Immunostaining and confocal microscopy of NETosis steps

$5x10^{4}$ sorted neutrophils were seeded in 24-well cell culture plates containing glass coverslips in HyClone HBSS +Ca, +Mg, - Phenol red (GE Healthcare) supplemented with 10mM HEPES (Invitrogen). Neutrophils were stimulated with 100nM PMA and fixed for 20min at room temperature with 2% paraformaldehyde at different timepoints. Cells were then washed and permeabilized with 0.5% Triton X-100 in PBS. Nonspecific binding was blocked with 2% BSA (Sigma) and 2% donkey serum (Sigma) in PBS. Samples were stained with 4′,6-diamidino-2-phenylindole dihydrochloride (DAPI; Life Technologies) and the primary antibodies: anti-MPO (R&D Systems), anti-Neutrophil elastase (GTX72042) and anti-PR3. Donkey anti-rabbit alexa488, anti-mouse alexa568 and anti-goat647. Fluorescence imaging was performed at a Leica TCS SP8 inverted confocal microscope using the sequential scan in between frames mode with a 63x objective.

Quantification of fluorescence granule signal

Intensity based thresholding mask was used on the PR3 channel (Otsu), particle analysis with a minimum size threshold of 10 squared microns was used to segment individual cells. Cells touching the image border were excluded from the analysis. Intensity of individual cells in the MPO and NE channel was measured and exported into a csv file.

Adoptive transfer and CFU assay of human neutrophil progenitors

Neutrophil progenitors were sorted from primary humanized recipient mice using FACS as described in Figure S4C in collection media (PBS+10%FBS). After cell sorting, neutrophil progenitors were pelleted and resuspended in order to inject 200,000 cells / 150μl / mouse. We use NSG-S $cKit^{w41/w41}$ as secondary recipient mice. After 4 days, we isolated the bone marrow of secondary recipient mice and use human/mouse chimera isolation kit (StemCell technologies) to remove the mouse hematopoietic compartment before staining to analyze by flow cytometry the presence of human neutrophils. CountBright Absolute Counting beads (Invitrogen) were added prior the sample acquisition to compare the absolute number of human neutrophils isolated after 4 days of $TET2^{Mut}$ or control neutrophil progenitor injection. For the colony forming unit (CFU) assay we sort and seeded 10,000 progenitor cells in methylcellulose (Methocult M3434, StemCell technologies) and after 10 days colonies were counted.

LPS in vivo challenge

Humanized mice reconstituted with wild type or TET2 mutant hHSCs were reversibly anaesthetized using 2.5% isoflurane. 50 μl of 0.25 mg/ml of LPS derived from *Escherichia coli* strain 0111:B4 (L4391, Sigma) or PBS were administered intranasally. Lungs were harvested on day 1 post-challenge.

Candida albicans in vivo challenge

Humanized mice reconstituted with wild type or TET2 mutant hHSCs were reversibly anaesthetized using 2.5% isoflurane. $1x10^6$ *C.albicans* CFUs in 100 μl 1xPBS or 1xPBS control was intratracheally administered. Lungs were harvested on day 1 post-challenge.

Lung sections and staining

Freshly extracted organs were fixed in 10% formalin, embedded in optimal cutting temperature (OCT) cryo-embedding media (VWR Chemicals BDH) and flash-frozen in a dry ice/100%ethanol. Frozen sections (8μm thickness) were dried and permeabilized with 0.5% Triton X-100 in PBS. Nonspecific binding was blocked with 2% BSA (Sigma) and 2% donkey serum (Sigma) in PBS. Samples were stained with 4′,6-diamidino-2-phenylindole dihydrochloride (DAPI; Life Technologies) and the following primary antibodies: 33G FITC-hCD45 (clone HI30, Biolegend), anti-MPO (R&D Systems) and anti-Ly6G-647 (clone 1A8, BioLegend), When required, fluorescently labelled secondary antibodies were used: donkey anti-goat IgG (Invitrogen). Fluorescence imaging was performed at a Leica TCS SP8 inverted confocal microscope using the sequential scan in between frames mode with a 40x objective and were scanned with an Olympus VS120 slide scanner. Scanned lung sections were analyzed using QuPath v0.4.3.[81] Cells on the selected tissues were segmented using the DAPI channel for cell detection with a minimum area of 10 and maximum area of 400 μm^2 and a cell expansion of 5 μm. hCD45+ cells or/and MPO positive cells were identified using intensity based single measurement classifier and composite classifier. *Candida albicans* area was measured using intensity based thresholding pixel classification on the *C.albicans* channel.

Human neutrophils from peripheral blood healthy donors

Blood from healthy volunteers was collected, after consent in heparin tubes and layered on Histopaque 1119 (Sigma-Aldrich) and centrifuged for 20 min at 800x g. The plasma, PBMC and -neutrophil layers were collected and neutrophils further fractionated on a discontinuous Percoll (GE Healthcare) gradient consisting of layers with densities of 1105 g/ml (85%), 1100 g/ml (80%), 1093 g/ml (75%), 1087 g/ml (70%), and 1081 g/ml (65%) by centrifugation for 20 min at 800x g. Neutrophil enriched layers were collected and washed.

RNA extraction, library preparation and RNA-sequencing of human neutrophils

Sorted neutrophils were pelleted and RNA was extracted using RNeasy Micro Kit (Qiagen, Cat#74004). Total RNA quality was verified in an Agilent 2100 Bioanalyzer (Agilent) and samples with RNA integrity number (RIN) of 8 or above were used prior to library preparation. Libraries were prepared using KAPA Stranded with RiboErase RNA-seq kit (according to the manufacturer's instructions). Briefly, 17–25 ng of starting RNA were subjected first to cytoplasmic and mitochondrial ribosomal RNA (rRNA) depletion by hybridization of complementary DNA oligonucleotides, followed by treatment with RNase H and DNase to remove rRNA duplexed to DNA and original DNA oligonucleotides. Samples depleted of rRNA were then subjected to 94 °C for 6 min in the 2×Fragment, Prime, and Elute Buffer in order to obtain 200–300 bp fragments. cDNA synthesis was run in two steps following the manufacturer's instructions. The ligation step consisted of a final volume of 110 μl of the adaptor ligation reaction mixture with 60 μl of input cDNA, 5μl of diluted adaptor, and 45 μl of ligation mix (50 μl of ligation buffer + 10 μl of DNA ligase). The Kapa Dual- Indexed Adaptors stock concentration was diluted to 1.5mM to get the best adaptor concentration for library construction. The ligation cycle was run according to the manufacturer's instructions. To remove short fragments such as adaptor dimers, 2X AMPure XP bead clean-ups were done (0.63 SPRI and 0.7 SPRI). To amplify the library, 15 PCR cycles were applied to the cDNA KAPA mix. Amplified libraries were purified using AMPure XP. The quality and fragment size distributions of the purified libraries were assessed with D1000 ScreenTape assay and reagents using Tapestation 42000 systems (Agilent Technologies, USA). Sequencing was then performed in a NovaSeq 6000 Sequencing System (Illumina) with 25 million single-end 100 bp reads/sample. Obtained FASTQ files were uploaded to the R environment where adapter and quality trimming was performed, and a quality control report was generated. Alignment was performed using the Salmon strategy under the R environment where FASTQ files were aligned to the grch38 human genome under the Salmon reference format. Obtained raw counts were then uploaded and analyzed using the package Deseq2 on R. Data normalization, regularization, and batch correction was performed from which PCA charts and pairwise comparisons between control and mutated cells were obtained. RNA-Seq analysis of the different stages of the neutrophil development was carried out in R using DESEq2 using a model that accounted for replicate, litter, and experimental group. Gene counts were normalised using variance stabilised transformation (VST), which were used for PCA analysis using the plotPCA function of DESeq2. Differential genes between groups were determined within DESeq2 using the IHW method for independent hypothesis weighting and the ashr method for log fold change shrinkage. To produce heatmaps of top differentially expressed genes, for each comparison, genes with an adjusted p-value of <= 0.05 were selected and ranked by their $\log_2$ fold change values. 500 differential genes were selected from the top and bottom of these lists to capture equal numbers of up and down regulated genes. These gene lists were concatenated, the VST normalised counts retrieved for all samples and transformed to z-scores. These z-score values were plotted using the pheatmap package within R. Gene set enrichment analysis (GSEA), was performed within R using the fgsea package against the Hallmark pathways from the Molecular Signatures database, using the ranked stat values from the non-shrunken differential analysis results. Scatterplots comparing the $\log_2$ fold changes of selected cell-type comparisons against genotype comparisons were produced by collecting genes with adjusted p-values of <= 0.05 in the cell-type comparison and matching these genes with the results from the genotype comparison. Log_2 fold changes for each gene for both comparisons could then be plotted as x vs y coordinates. A line of best fit was calculated using a linear model within R and plotted using ggplot2.

Analysis of control and preleukemic human neutrophils with datasets GSE150021 (neutrophils form human bone marrow) and GSE109467 (neutrophils from mouse)

To compare the transcriptional signature of the human neutrophils isolated from the bone marrow of humanized mice with the neutrophils isolated from human bone marrow or mouse bone marrow, we downloaded GSE150021 and GSE109467 datasets, respectively from GEO database. GSE150021 dataset was uploaded to the Deseq2 package along with the raw counts from control and mutated samples where normalization and batch effect correction were performed. For the dataset GSE109467 containing mouse samples, the ortholog genes between mouse and human were obtained for both datasets and used for the analysis. Pairwise comparisons and PCA analyses were performed for both analyses.

Antimicrobial functional assay

Candida albicans culture

Wild-type (used for hyphal growth assay and in vivo challenge) or Yeast locked (used for phagocytosis assay) *C. albicans* was cultured overnight shaking at 37°C in YEPD. A sub-culture was made the next day to an optical density (A600) of 0.4-0.8 in YEPD (for yeast) or Roswell Park 26 Memorial Institute 1640 medium (RPMI; Thermo Fisher Scientific) (for hyphae) for 4 hours.

Hyphal growth assay

Live hyphae were used at an MOI=0.5 in a 96-well plate containing 1.5x10^5 human neutrophils in HyClone HBSS +Ca, +Mg, - Phenol red (GE Healthcare) containing 10mM HEPES (Invitrogen). DNA of live cells was stained with 4μg/ml Hoechst (membrane permeable; Thermo Scientific) and DNA from dead cells with 0.2 μM Sytox-orange (membrane impermeable; Invitrogen). The cells were imaged on an inverted Nikon wide-field microscope system at 37°C and CO_2 (5%). Four fields of view were acquired per well every 30 mins for 10 hours using a 40x objective. The length of single hyphae was quantified using Fiji/ImageJ version 2.0.0 software for snapshots obtained every 60 min.

Phagocytosis assay

Heat inactivated yeast locked *C. albicans* were used at an MOI=10 or opsonized 488-Zymosan bioparticles from *S.cerevisae* (Z23373 ThemoFisher) used following the manufacturers instruction, was added to 5x10^4 human neutrophils in HyClone HBSS + Ca, +Mg, - Phenol red (GE Healthcare) containing 10mM HEPES (Invitrogen) and imaged using a wide-field microscope system at 37°C and CO_2 (5%). Phagocytic events were quantified by detecting the number of phagocytosed particles per cell using Fiji.

DNA methylome analysis

1x10^5 human neutrophils were sorted from each humanized mice (we include 2 biological replicates), pelleted and sent to ActiveMotif for Reduced Representation Bisulfite Sequencing (RRBS). According to manufacture instructions, sequencing and analysis of the data were done as follows. Single-end 75 nucleotide sequencing reads are generated by Illumina sequencing using a NextSeq 500. The Illumina adapter sequence is trimmed from reads using Trim Galore, and then a custom script is used to trim additional bases that are added during the library creation process to facilitate sequencing. Reads are mapped to the genome using Bismark and Bowtie 2 allowing for no mismatches and a seed substring length of 20. Following alignment, PCR duplicates are removed using a custom analysis script. Each read has a randomized barcode and if more than one read has the same start and end coordinates and the same barcode, then all but one of the reads are discarded. Alignment information of the remaining reads are stored in BAM files. Methylation at CpG sites is then extracted from the BAM files using Bismark and reported in bedGraph, coverage, and CpG report formats. CpG reports from the Bismark alignment are processed with the methylKit R package, and only CpG sites covered with at least 10 reads are retained for the downstream analyses. The average methylation at each chromosome and at each annotated transcript are determined on a per-sample basis. In addition, summary graphics showing the amount of CpG methylation and read coverage are generated per sample. For the region-based analysis, the genome is tiled into 1 kilobase regions and significantly differentially methylated tiles are identified. For pairwise comparisons a Fisher's exact test is used to identify differentially methylated sites. Statistical significance of a given differential region or base is indicated by adjusted p-value (q-value) after false-discovery correction with methylKit's default SLIM method. Volcano Plots and bar plots were generated in an R environment (v 4.1.1) from differentially methylated CpG bases or differentially methylated regions, respectively (Table S3) using the packages ggplot2 (v 3.3.5) and tidyverse (v 1.3.1). Pathway analysis was carried out using the function "enrichGO" from the package "clusterProfiler" (v 4.2.2) within the R environment mentioned before.

Western blot

Cells were lysed in 300 μl RIPA lysis buffer (Sigma, SLCC5042) with 100X Protease inhibitor Cocktail (Sigma, P8340) for 30 minutes on ice and then sonicated for 5 minutes with Pico Bioraptor. Protein concentration was quantified using the BCA assay (Bio-Rad, Cat#5000116). Gel electrophoresis was performed for 30 minutes at 20 V and for 2 hours at 100 V using 4 to 12%, Bis-Tris, Mini Protein Gels (Invitrogen, Cat#NP0335BOX). Wet transfer was performed with Immuno-Blot PDVF membranes (Bio-Rad, Cat#162-0177) for 2.5 hours at 100 V and 400mA on ice. Membranes were blocked with 5% milk solution and immunoblotted with specific primary antibodies anti-TET2 (Cell Signaling, Cat#45010) or anti- β-Actin (Cell Signaling, Cat#3700), both diluted in 1:1000. Membranes were then washed 3 times with TBS-Tween 0.1% and incubated with anti-Rabbit Ig or anti-Mouse Ig horseradish peroxidase (HRP) conjugated secondary antibody (Cell Signaling, Cat#7076 and #7074 respectively) for 3 hours at room temperature. After washing three times with TBS-Tween 0.1%, membranes were incubated for 5 minutes with Immobilon Crescendo Western HRP

substrate (Millipore, Cat#WBLUR0500) and then visualized using Amersham ImageQuant 800Imager (Cytiva Life Sciences, Cat#29399481).

ATACSeq sample preparation, sequencing and analysis

$5x10^4$ control and $5x10^4$ $TET2^{Mut}$ neutrophils were sorted from the same humanized mouse engrafted with a mix of wild-type and $TET2^{Mut}$ hHSPCs as shown in Figure S2B (4 biological replicates were included). Improved OMINI-ATAC protocol was followed as described previously.[82] Library PCR amplification was done with 9 cycles and double-sided size selection was performed using KAPA Pure beads (Roche, 07983280001). Sequencing was then performed in a HiSeq4000 Sequencing System (Illumina) with a loading molarity of 200pM plus 10% Phix (illumine). Each sample was sequenced to achieve 30 million Pair-end 100 bp reads/sample. For the analysis of percentage of fragmented DNA and differentially annotated peaks we use the nf-core pipeline within the nextflow bioinformatics workflow framework (version 19.10.0). FASTQ files were aligned to the GRCh38 human genome. For the transcription factor motif enrichment analysis, we used gProfiler, where we uploaded the peaks annotated to promoter regions and with Score > 2000. To represent Figure S3F the number of genes regulated from different motifs for the same TF were pooled.

Time-lapse imaging and NET area quantification assay

$5x10^4$ human neutrophils were seeded in a black 96-well plate (PerkinElmer) in HyClone HBSS +Ca, +Mg, - Phenol red (GE Healthcare) containing 10mM HEPES (Invitrogen). DNA of live cells was stained with 4μg/ml Hoechst (membrane permeable; Thermo Scientific) and DNA from dead cells with 0.2 μM Sytox-green (membrane impermeable; Invitrogen). The cells were stimulated with 100 nM PMA at the start of the microscope acquisition. An inverted Nikon wide-field microscope system at 37°C and CO_2 (5%) was used for the imaging. Four fields of view were acquired per well every 15 mins for 10-15 hours using a 40x objective. NET area was quantified at min 200 for Figure 4D using FIJI. Otsu intensity, based thresholding, was used to generate a mask to quantify the Sytox signal area.

ROS assay

$1x10^5$ neutrophils were seeded in a white 96-well plate (Nunc) in 100 μl HyClone HBSS +Ca, +Mg, - Phenol red (GE Healthcare) containing 10mM HEPES (Invitrogen). Additional 50 μl containing 1.2 U/ml of HRP and 100 μM Luminol was added to each well. The cells were stimulated with 100 nM PMA and chemiluminescence signal was recorded every 60 seconds for 6 hours at 37°C in a TECAN Spark platereader.

NET degradation analysis and quantification

Pixel classification with the "pixROI" method

For each video, we classified the pixels into three regions of interest: background, netotic and necrotic pixels, using a method referred to as pixROI. The classification was determined by a criterion based on the general aspect of the pixel's Sytox signal curve.

First, the Sytox video was smoothed along the time axis with a gaussian convolution. Then the Sytox signal was normalized as follows. A common background was roughly estimated by intersecting all non-trivial regions in the frames with values below the Otsu threshold of the frame. The common background was thus only contained pixels which had never been part of a cell or a net. Then, averaging over space at each timepoint gave the mean common background signal. This corresponded to a time-variable scaling curve that we used to normalize the Sytox signal and obtain a background-rescaled signal. This allowed for standardizing the aspect of all pixel signal curves by ensuring that the aspect of the rescaled curves was not due to video contrast variations and outlier frames, but only attributed to biological changes in the signal intensity.

Next, we determined the timepoints at which the Sytox curves were decreasing or increasing by computing the signs of the discrete derivative over time. Then we applied the following criteria to determine which region the pixel belonged to:

- If the maximal value of the background-rescaled signal was greater than 5 and the curve spent more than 50% of the time increasing after its minimal value and its final value was more than 90% of the maximum, the pixel was classified as necrotic
- If the maximal value of the background-rescaled signal was greater than 5 and the curve spent more than 50% of the time decreasing after its maximal value and its final value was less than the maximum, the pixel was classified as netotic
- Otherwise, the pixel was classified as a background pixel.

After dividing this pixel-wise classification into three regions with pixROI, the temporary background-rescaled signals and common background were not used in any further analysis.

Netotic signals and peaking times

For each video, we sampled 1000 random pixels classified as netotic by the pixROI method, and collected their *raw* (unscaled, smoothed) Sytox signals. We also retained their peaking times. As individual cells could undergo netosis at different timepoints, we aligned the selected Sytox signals to their respective peaking time to focus on the post-peak signal behavior.

For each experimental condition (a row), we separated the collected signals into two groups corresponding to control and TET2 mutant types. The post-peak degradation of the signal intensity was then revealed by computing the median value of all the signals, as well the 25% and 75% quartiles Q1 and Q3.

NET lifetimes and normalization

To study the relative speed of NET degradation, the collected signals were normalized between 0 (minimal value before peak) and 1 (maximal value). We computed the median signal of all the normalized signals, as well their 25% and 75% quartiles signal Q1 and Q3. Then we computed the *50%-lifetime and 80%-lifetime* of the median, Q1 and Q3 curves, by measuring the amount of time needed for the curves to decrease from 1 (normalized peak) to.5 or.2.

Cell counting

We estimated the initial number of cells in the field of view with the first frame in the DAPI channel. We also estimated the number of necrotic cells by using the pixROI classification. In both cases, we counted components whose area was comprised between two values (between 1000 and 4000 pixels covered for valid cell kernels in the DAPI channel; between 5000 and 10000 pixels covered for valid necrotic cells). Then, under the simplifying assumption that the initial number of cells corresponded to the sum of the number of netotic cells and that of necrotic cells, we obtained an estimation of the number of netotic cells as well.

Net span

The net span for each experimental condition (row) and type (control or TET2 mutant) was estimated as follows. For each video (site), we computed the total net coverage given by the pixROI classification. Then, we divided this total area by the estimated number of netotic cells obtained above. We thus obtained a value *underestimating the actual individual net span per netotic cell*, since nets would often overlap with each other.

Nuclear morphology quantification

Segmentation of the multilobed nuclei was performed using a 2-class pixel random-forest classifier trained in Ilastik Version 1.4.0.[83] The two classes (background and foreground) were manually annotated and then used to perform classification and segmentation across the entire image set (control and $Tet2^{Mut}$). The multilobed nature of neutrophil nuclei does not allow for star-convex segmentation methods such as StarDist, or any segmentation method which minimizes the perimeter of a nucleus as a convex hull. For this reason, we used the random forest pixel based classifier Ilastik.

These nuclear segmentation images were imported into CellProfiler version 4.2.1.[84] The ConvertImageToObjects module of CellProfiler was used to convert the segmentation image into individual primary objects. All objects that were touching the image border were filtered out, and the remaining objects were filtered by a minimum size (250 pixels) to discard any fractured nuclei. The MeasureObjectSizeShape module was then used to measure object shape parameters, including perimeter, area and form factor. These measurements were made for each primary object in each image, and exported into a csv file.

Scanning electron microscopy

A monolayer of neutrophils was seeded, stimulated, and fixed in glass coverslips as for fluorescence microscopy. After fixation samples were treated with 1% reduced osmium for 60 mins at 4°C. Cells were then dehydrated in a graded ethanol series of 70%/90%/100%. Samples were then subjected to a Leica CPD300 critical point dryer and a platinum coat was applied with a Quorum 150R Sputter Coater. Samples were imaged using the scanning electron microscope QUANTA250 SEM.

Transmission electron microscopy

For electron microscopy, 250K neutrophils were fixed in 4% (v/v) formaldehyde (Taab Laboratory Equipment Ltd, Aldermaston, UK) in 0.1M phosphate buffer (PB) pH7.4 at 4 degrees for 1h and stored overnight in 1% (v/v) formaldehyde (Taab) in 0.1M PB pH7.4 at 4 degrees. The day after, cells were processed using a Pelco BioWave Pro+ microwave (Ted Pella Inc, Redding, USA). Each step was performed in the Biowave, except for 3, 3'-diaminobenzidine (DAB) staining and PB / dH_2O washes, which consisted of 1 wash on the bench and two washes in the Biowave without vacuum (at 250W for 40s). Each step before embedding in agarose was followed by centrifugation at 1000g for 5min in a swinging bucket centrifuge. All the chemical incubations were performed in the Biowave for 14min under vacuum in 2min cycles alternating with/without 100W power, with SteadyTemp plate set to 21ºC. In brief, the pellets were fixed in 2.5% (v/v) glutaraldehyde (Taab) / 4% (v/v) formaldehyde in 0.1M PB pH 7.4, washed in 0.1M PB, and stored in 1% (v/v) formaldehyde in 0.1M PB pH 7.4 at 4 degrees until further processing. Pellets were then stained with 2% (v/v) aqueous osmium tetroxide (Taab), washed in dH_2O. Cells were then stained with 0.5 mg/mL DAB / 0.02% hydrogen peroxide in 0.1M PB pH 7.4 for 25 min on ice and washed in dH_2O before being mixed 1:1 with 3% aqueous low melting point agarose (Sigma), spun down at 1000 g for 10 min at 30ºC and put on ice for 20min. Agarose blocks were then removed from the tube, transferred to a 24-well plate and incubated in 2% (w/v) aqueous uranyl acetate (Agar Scientific, Stansted, UK). The samples were then washed in dH_2O and dehydrated in a graded ethanol series (25%, 50%, 70%, 90%, and 100%, twice each), and in acetone (3 times) at 250 W for 40s without vacuum. Exchange into Epon 812 resin (Taab) was performed in 25%, 50% and 75% resin in acetone steps, followed by 4 pure resin steps, at 250 W for 3 min, with vacuum cycling (on/off at 30 sec intervals), before embedding at 60ºC for 24 h. The blocks were then mounted for micro-computed tomography (micro-CT) on a cylindrical specimen holder. Tomographic imaging was conducted in an Xradia Versa 510 (Carl Zeiss Ltd, Cambridge, UK). A low-resolution scan was captured at 40kV/3W, with a pixel size of 3-5μm. The data were exported as tiff and the region of interest was identified in each block using Crosshair plugin in fiji.[85] 70nm sections were cut using a Leica UC7 ultramicrotome (Leica Microsystems, Vienna, Austria) and picked up on 1x2mm slot copper grids (Gilder Grids Ltd., Grantham, UK). A section through the pellet containing at least 100 cells was viewed in each condition using a 120kV JEOL

JEM-1400Flash Electron Microscope (JEOL Ltd., Welwyn Garden City, UK) and images were captured with JEOL's Matataki Flash camera. A subset of neutrophils with two or more nuclear lobes, identifiable neutrophil granules and good ultrastructural preservation and DAB staining was used for quantification.

QUANTIFICATION AND STATISTICAL ANALYSIS

Statistical analyses were performed using GraphPad Prism software (GraphPad). Results are depicted with the individual values and indicating mean ± SEM in each case. The statistical test used is specified in each figure legend. In brief, for CyTOF analysis, two-way ANOVA test was used and for flow cytometry analysis unpaired t-test was used. For ATACSeq, statistical analysis was done by paired t-test comparison between the control and TET2^{Mut} neutrophils coming from the same mouse. For the functional assays, Mann-Whitney test for single comparisons between WT and TET2^{Mut} was used.

Cell Stem Cell, Volume *30*

Supplemental Information

Loss of TET2 in human hematopoietic stem cells alters the development and function of neutrophils

Hector Huerga Encabo, Iker Valle Aramburu, Manuel Garcia-Albornoz, Marion Piganeau, Henry Wood, Anna Song, Alessandra Ferrelli, Aneesh Sharma, Carlos M. Minutti, Marie-Charlotte Domart, Despoina Papazoglou, Kristian Gurashi, Miriam Llorian Sopena, Robert Goldstone, Todd Fallesen, Qian Wang, Linda Ariza-McNaughton, Daniel H. Wiseman, Kiran Batta, Rajeev Gupta, Venizelos Papayannopoulos, and Dominique Bonnet

Figure Supplementary 1

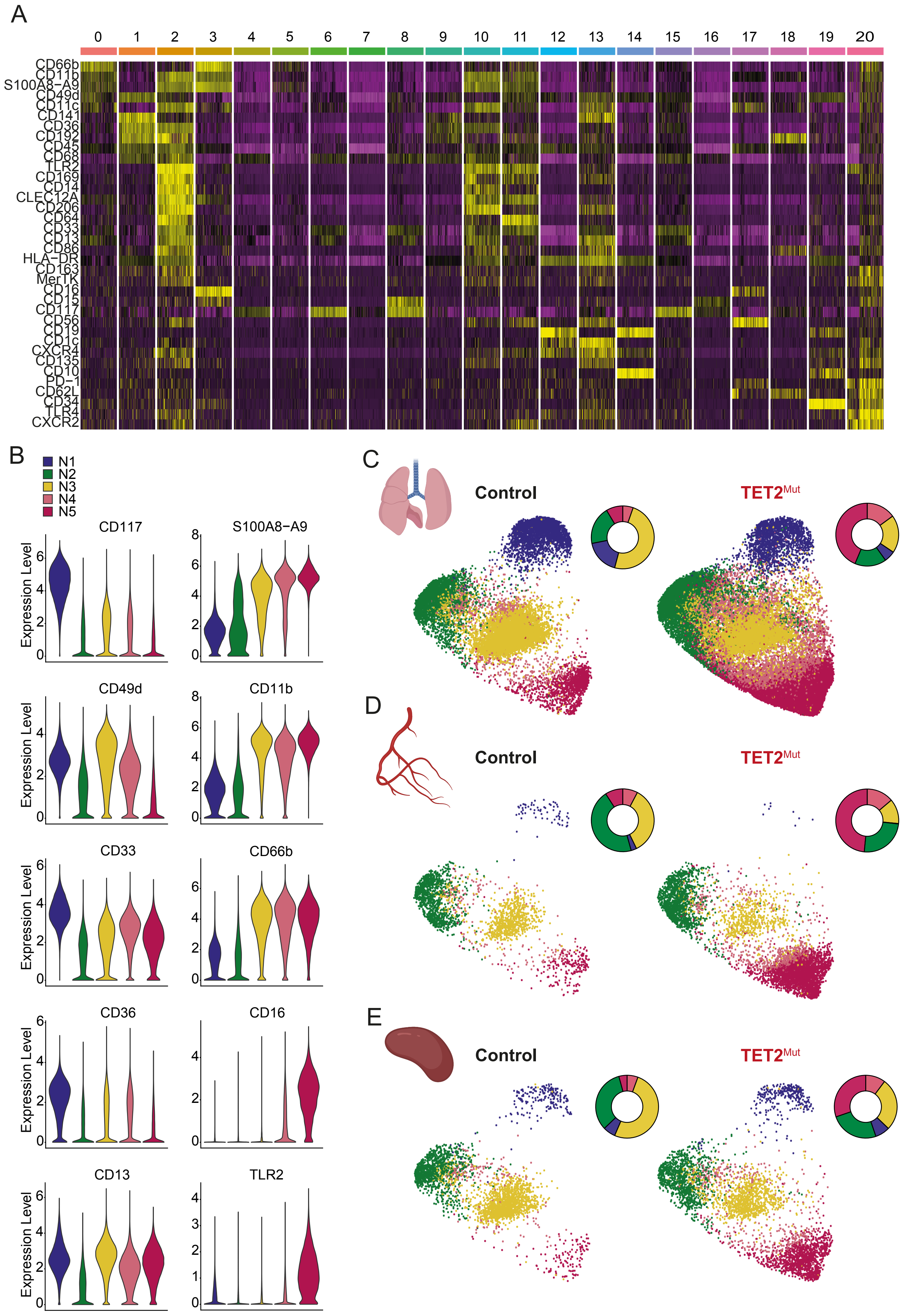

Figure S1. Mass cytometry analysis of neutrophil subsets in bone marrow and peripheral tissues of NSG mice engrafted with $TET2^{Mut}$ hHSCs. Related to Figure 1.

A. Heatmap showing the expression of the markers used in the CyTOF panel for each of the 21 clusters annotated in Fig. 1B-C.

B. Violin plots showing the expression of markers associated with different stages of the neutrophil differentiation across N1-N5 subsets.

C. Comparison of the neutrophil heterogeneity in the lung derived from control (14,983 cells) and $TET2^{Mut}$ (34,695 cells) hHSPCs by UMAP and pie-chart representing the percentage of each subset.

D. Comparison of the neutrophil heterogeneity in the blood derived from control (2283 cells) and $TET2^{Mut}$ (5,337 cells) hHSPCs by UMAP and pie-chart representing the percentage of each subset.

E. Comparison of the neutrophil heterogeneity in the spleen derived from control (3,093 cells) and $TET2^{Mut}$ (3,943 cells) hHSPCs by UMAP and pie-chart representing the percentage of each subset.

Figure Supplementary 2

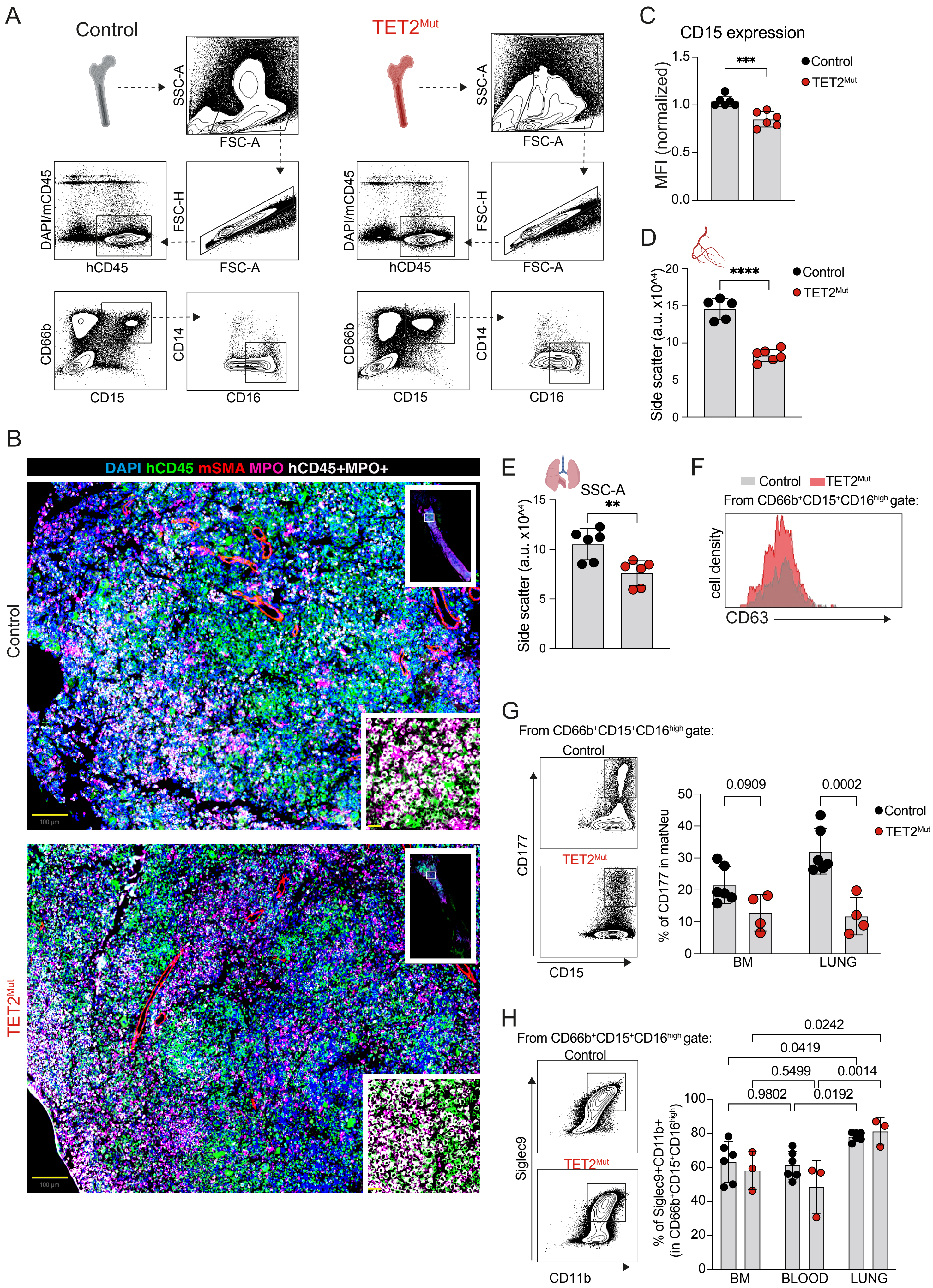

Figure S2. TET2^{Mut} hHSPCs exhibit exacerbated neutrophilia in the bone marrow and the lung. Related to Figure 2.

A. Gating strategy to identify human mature neutrophils in the bone marrow of mice reconstituted with control (black) or TET2^{Mut} (red) hHSPCs.

B. Representative large images from bone sections (top right) of mice engrafted with control or TET2^{Mut} hHSPCs. Enlarged areas (original magnification, 20X, scale bar: 100µm) to visualize the human hematopoietic system (green), vasculature (red) and MPO signal (pink). Amplified sections (bottom right, scale bar: 20µm) displaying human neutrophils co-stained with hCD45 and MPO (white signal).

C. Quantification of mean fluorescence intensity (MFI) of CD15 expression in CD66b^{+}CD15^{+}CD16^{high} neutrophils. (n = 6 mice, data from 3 independent experiments). For each experiment, CD15 MFI of one control neutrophil population was normalized to 1. Unpaired t test, *** p<0.005.

D-E. Quantification of the side scatter-area (SSC-A) of human CD66b^{+}CD15^{+}CD16^{high} neutrophils from the blood (D) and the lung (E) of humanized mice. (n = 5-6 mice, data representative of 3 independent experiments). Unpaired t test used for significance, ** p<0.01; **** p<0.001.

F. Representative histogram of CD63 expression in CD66b^{+}CD15^{+}CD16^{high} neutrophils. See Figure 2E for quantification.

G. Representative density plots of CD177^{+} neutrophils within the CD66b^{+}CD15^{+}CD16^{high} cell population in the bone marrow (BM) and quantification of these cells in BM and lung of humanized mice reconstituted with control (black, n = 6) or TET2^{Mut} (red, n = 3) hHSPCs. Unpaired t test used for significance. p-values are displayed.

H. Representative density plots of Siglec9^{+}CD11b^{+} neutrophils within the CD66b^{+}CD15^{+}CD16^{high} cell population in the bone marrow (BM) and quantification of these cells in the BM, blood and lung of humanized mice reconstituted with control (black, n = 6) or TET2^{Mut} (red, n = 3) hHSPCs. Unpaired t test used for significance. p-values are displayed.

Figure Supplementary 3

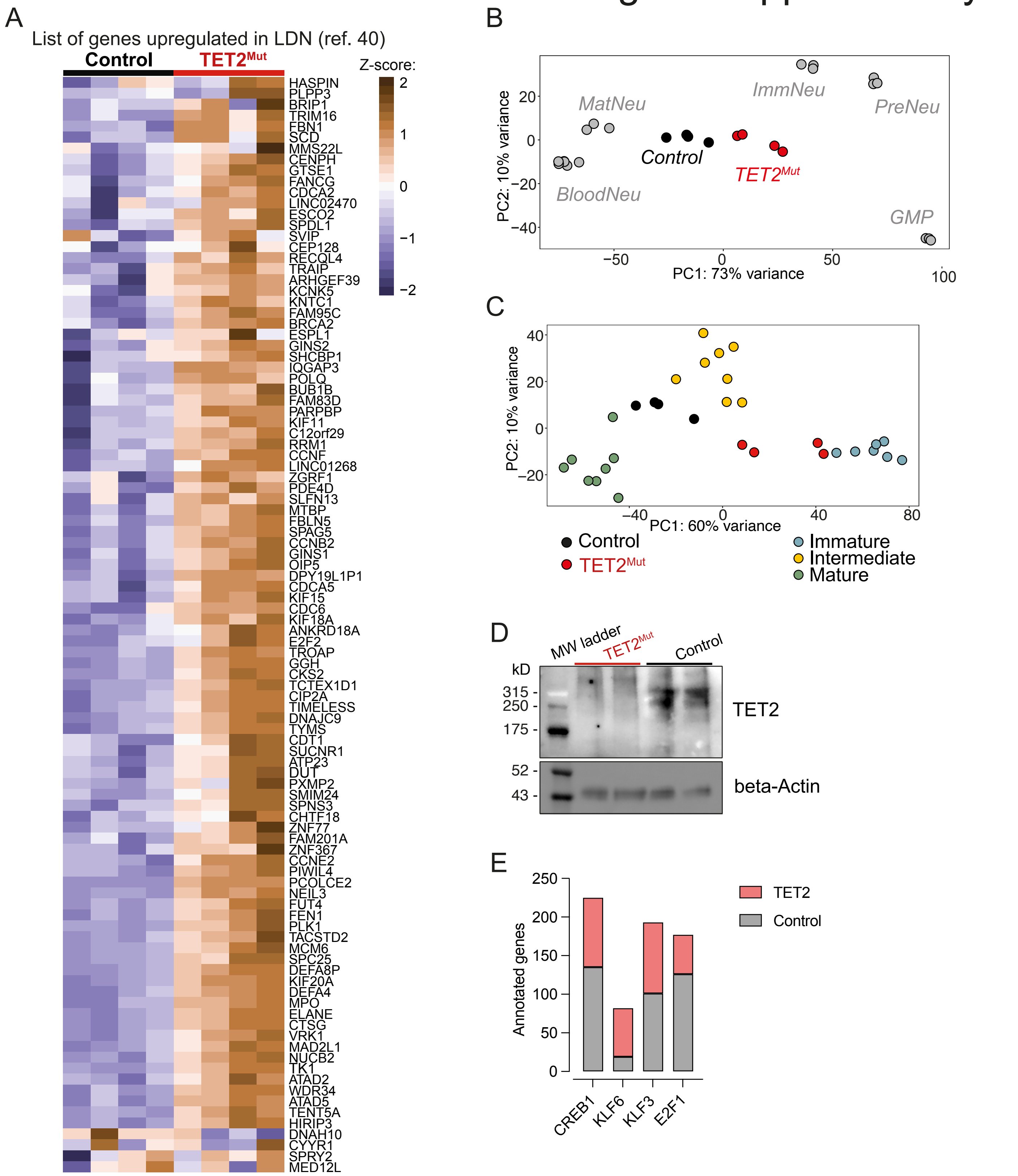

Figure S3. The transcriptome of TET2^{Mut} neutrophils is related to immature neutrophils. Related to Figure 3.

A. Heatmap showing the expression level of the gene set previously associated with LDN in neutrophils derived from control (black) or TET2^{Mut} (red) hHSPCs.

B. Principal component analysis comparing the transcriptional profile of our control (black) and TET2^{Mut} (red) neutrophils with the different bone marrow neutrophil subsets identified in the dataset GSE109467: GMP (granulocyte-myeloid progenitor), PreNeu (pre-neutrophils), ImmNeu (immature neutrophils), MatNeu (mature neutrophils) and BloodNeu (blood neutrophils). Analysis was done after filtering for ortholog genes between human and mouse.

C. Principal component analysis comparing the transcriptional profile of our control (black) and TET2^{Mut} (red) neutrophils with the 3 subsets (immature, intermediate and mature) of human neutrophils identified in the dataset GSE150021.

D. Western blot of TET2 performed in human CD66b+ cells isolated from the bone marrow of mice engrafted with control (black) or TET2^{Mut} (red) hHSPCs.

E. Transcription factor motif enrichment analysis from annotated peaks in control (grey) or TET2^{Mut} (red) neutrophil genome generated by gProfiler. The bars represent the number of annotated genes regulated by each transcription factor. See Table S3 for complete list of TF motifs and annotated genes.

Figure Supplementary 4

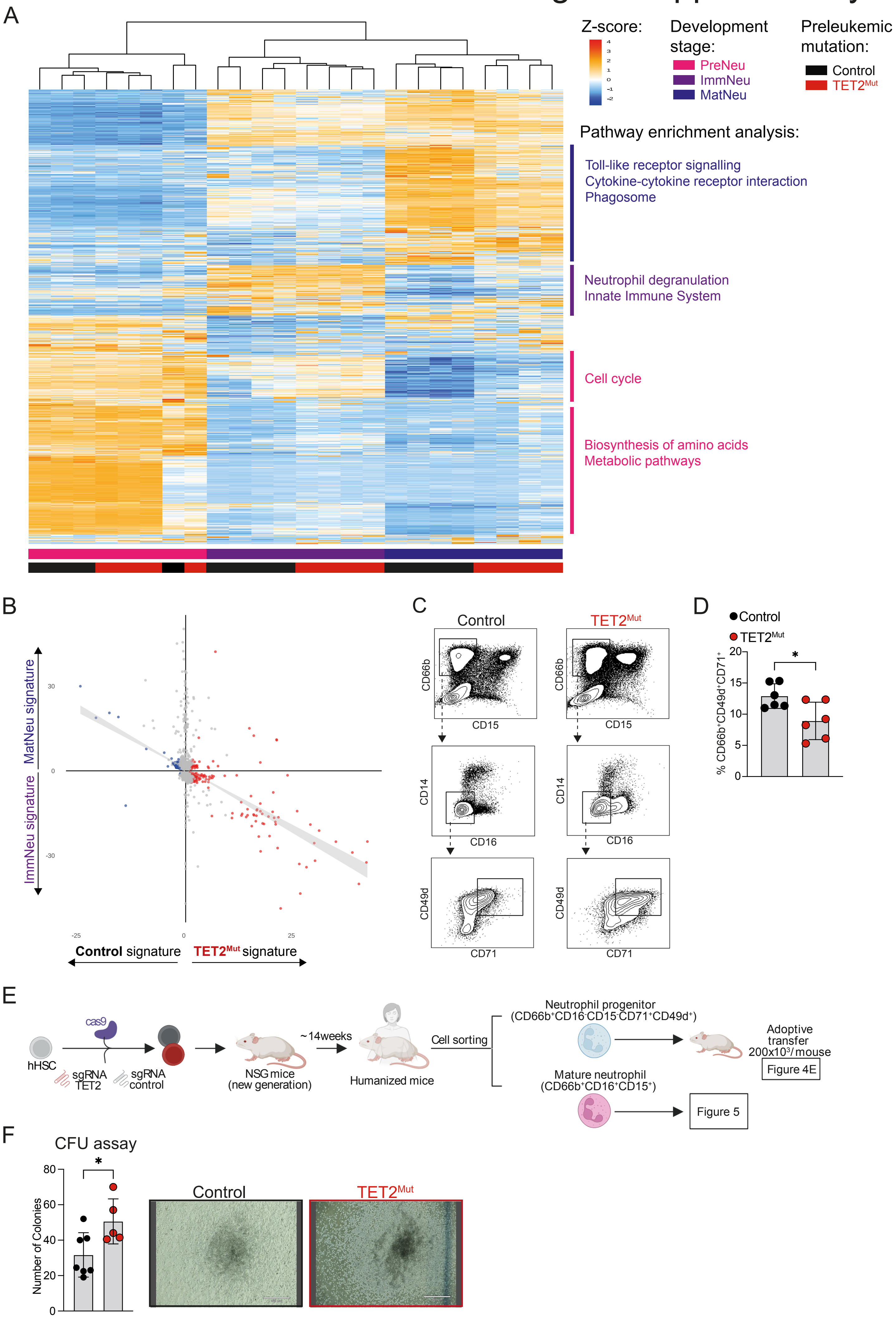

Figure S4. TET2 mutations impact on neutrophil precursors and transcriptome identity over different stages of the neutrophil development. Related to Figure 4.

A. Heatmap of the top 500 differentially expressed genes among the different cell populations. Enrichment pathway analysis was done by selecting characteristic gene sets for each cell state as indicated. See Table S4.

B. Scatterplots comparing the log2 fold changes of genes with adjusted p-values of <= 0.05 in the immNeu vs matNeu (y axis) comparison with the log2 fold changes of genes with adjusted p-values of <= 0.05 that appear downregulated (blue) or upregulated (red) in $TET2^{Mut}$ matNeu compared to control counterparts (x axis).

C. Representative gating strategy to identify and sort the neutrophil precursors (defined as $CD66b^{+}CD49d^{+}CD71^{+}CD14^{-}CD15^{-}CD16^{-}$) to perform the adoptive transfer.

D. Percentage of neutrophil precursors in the bone marrow of control or $TET2^{Mut}$ humanized mice. n = 6 mice, data representative of 3 independent experiments, each performed with hHSPCs from different human donors. Each dot represents one humanized mouse and error bars: mean ± SEM. Unpaired t test used for significance, * p<0.05.

E. Schematic representation of the generation humanized mice to study TET2-derived CH and the functional impact of TET2 mutations in human neutrophil progenitors and mature neutrophils. Created with BioRender.

F. Quantification of the colonies counted in the in vitro CFU assay. n = 5-7 mice, data from 2 independent experiments; each dot represents one biological donor and error bars: mean ± SEM. Unpaired t test used for significance, * p<0.05; Representative pictures of individual colonies are displayed at the right.

Figure Supplementary 5

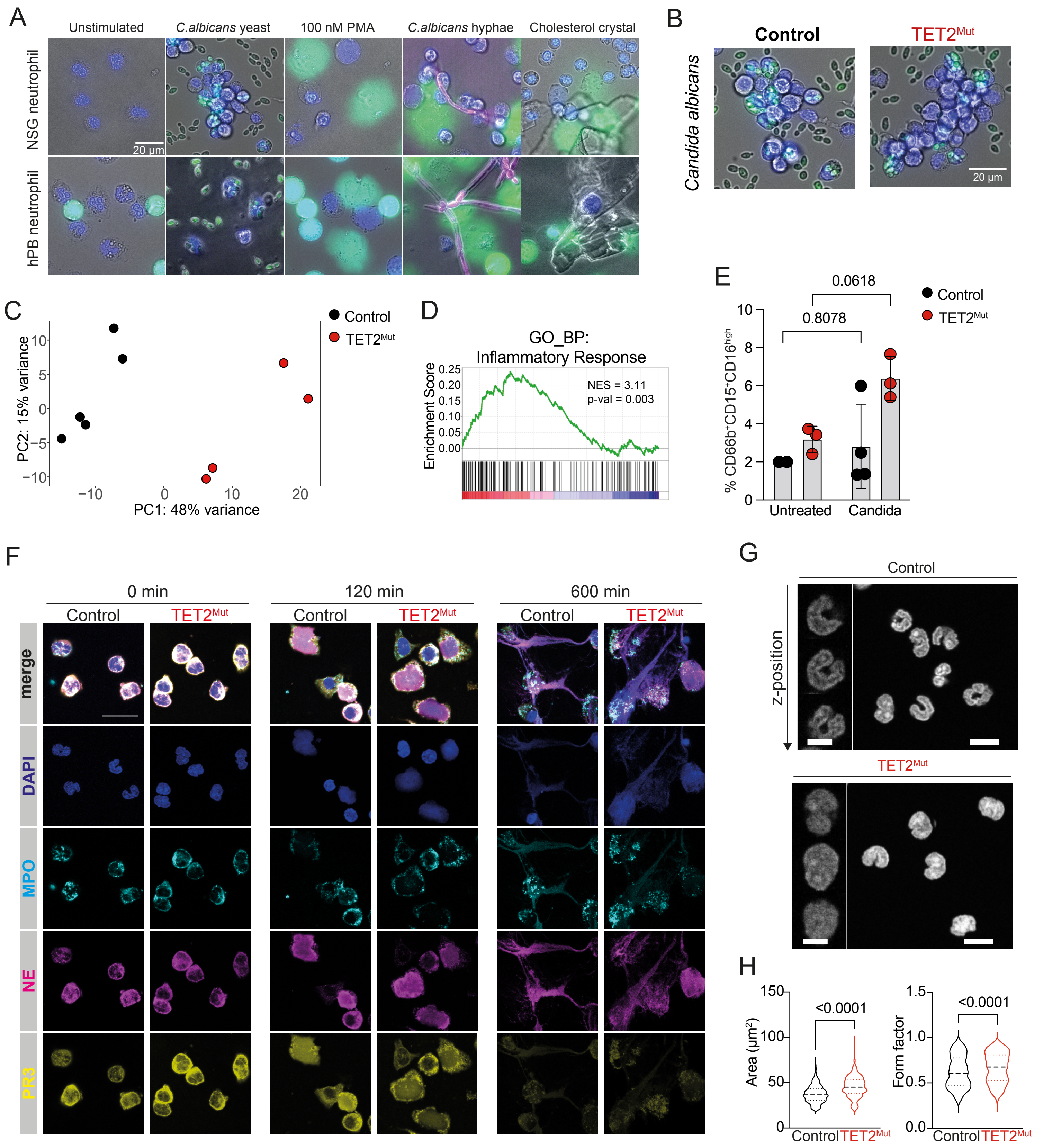

Figure S5. Exacerbated inflammatory response by TET2^{Mut} neutrophil upon LPS or *C.albicans* stimulation. Related to Figure 5.

A. Selection of snapshots of wide-field fluorescence microscopy images from timelapse imaging. Sorted human neutrophils from humanized mice (upper panel) and neutrophils isolated from peripheral human blood (lower panel) were stimulated with 100 nM PMA, yeast heat inactivated *C.albicans* (green), life *C.albicans* hyphae (magenta) and cholesterol crystals. Nuclei are stained with Hoechst and NETs are stained with sytox green. Scale bar 20 microns.

B. Representative fields of view used in the quantifications of phagocytosis capacity of control and TET2^{Mut} neutrophils of yeast heat inactivated *C.albicans*. Nuclei are stained with Hoechst and *C.albicans* are stained with sytox green. Scale bar 20 microns.

C. RNA-sequencing principal component analysis of control (black) and TET2^{Mut} (red) neutrophils after 3 hours of LPS stimulation (1μg/ml). n = 4 mice.

D. Gene Set Enrichment Analysis (GSEA) showing upregulation of inflammatory response pathway using the Gene Ontology biological process (GO_BP) database. See Table S5 for complete list of pathways.

E. Quantification by flow cytometry of human neutrophil percentage in the lung of mice engrafted with control (black) or TET2^{Mut} (red) hHSPCs in homeostasis (untreated) or 24 hours post *C.albicans* challenge. One-way ANOVA test used for significance; p-values are displayed in the figure.

F. Representative confocal microscopy images of sorted human control and TET2^{Mut} neutrophils stimulated with 100 nM PMA and fixed at timepoints 0, 120 and 600 minutes. Cells were stained with antibodies against MPO (cyan), NE (magenta) and PR3 (yellow). Nuclei are stained with DAPI. Scale bar 20 microns.

G-H. Representative confocal microscopy image (G) and quantification (H) of nuclear shape from sorted human control and TET2^{Mut} neutrophils stained with DAPI shown as a z-montage of a selected nucleus (scalebar 5 microns) or a selection of nuclei (scalebar 10 microns). H. Violin plot of quantified area or form factor per cell of neutrophils obtained from 3 different mice per group. Unpaired t test was used for significance.

Figure Supplementary 6

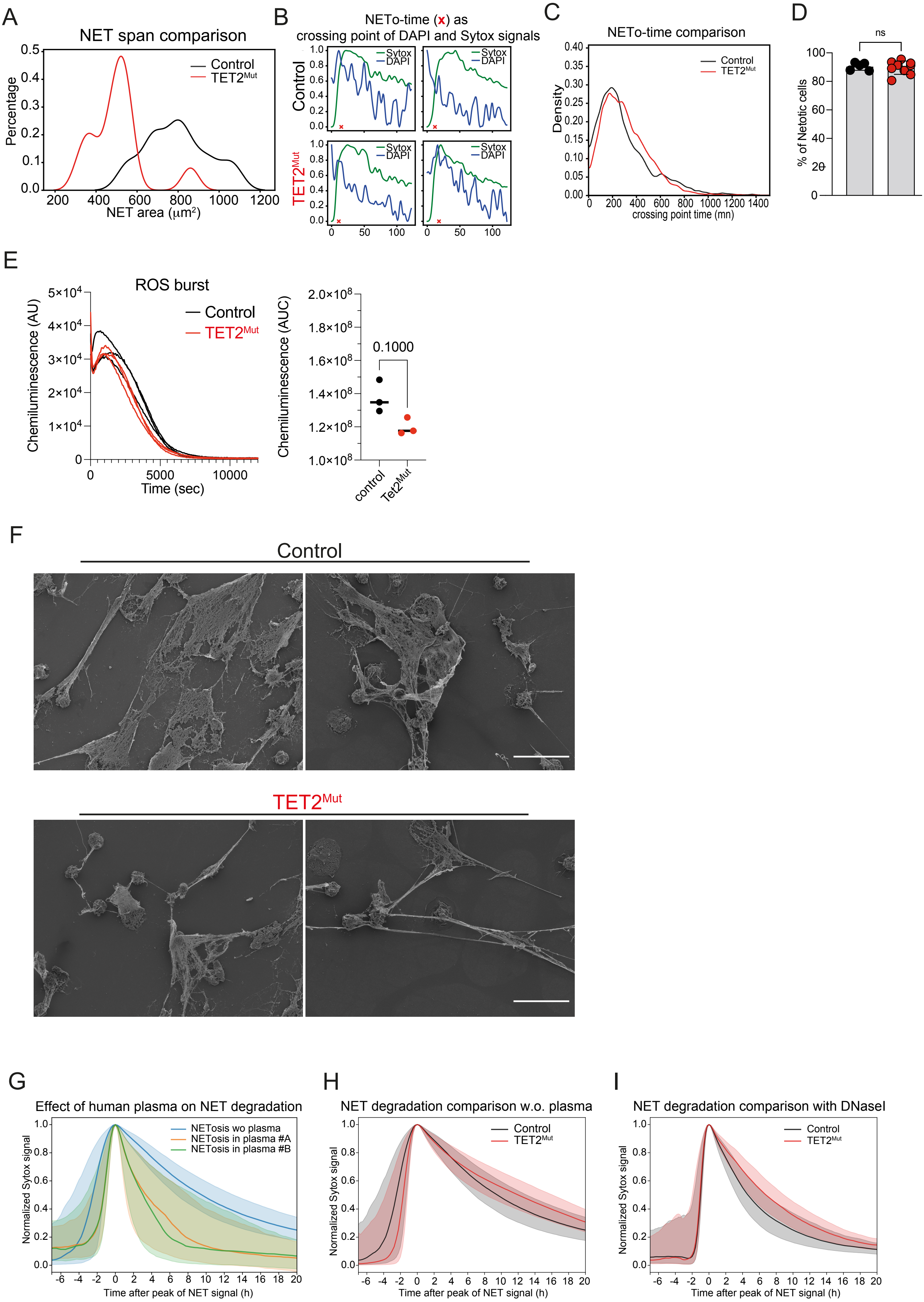

Figure S6. Analysis of NET formation and clearance in TET2^{Mut} neutrophils. Related to Figure 6.

A. Distribution of NET spans for control and TET2^{Mut} neutrophils. The NET span was estimated for each video as the contribution of an individual netotic cell to the total NET coverage based on the pixROI classification. The total NET coverage corresponds to the maximal spatial span ever reached by all possible NETs formed over time.

B. Examples of crossing time points between Sytox and DAPI signal used to estimate times of NETosis. NETosis is assumed to happen when the normalized DAPI signal crosses the normalized Sytox signal.

C. Distributions of NETo-times (times of NETosis) for control and TET2^{Mut} neutrophils. The distribution is computed after gathering 1000 random netotic crossing points for all videos corresponding to the mouse type and experimental condition.

D. Percentage of control and TET2^{Mut} neutrophils undergoing NETosis upon 100nM PMA. Each dot represents one biological replicate. Data representative from 2 independent experiments. Control neutrophil (black dots) n = 5; TET2^{Mut} neutrophils (red dots) n = 9.

E. ROS production by control and TET2^{Mut} neutrophils upon PMA stimulation, detected by chemiluminescence of luminol over time (left panel). Area under the curve (AUC) of each ROS burst (right panel). n=3 per group, representative of 2 independent experiments.

F. Scanning electron microscopy images of NETs formed upon 100 nM PMA stimulation from control or TET2^{Mut} neutrophils sorted from the bone marrow of humanized mice. 1000x magnification was used with dwell times of 10 microseconds. Scale-bar 30 microns.

G. NET degradation traces from control neutrophils in the absence and presence of human plasma. Median curves (solid lines) in the absence of plasma (blue line) or in the presence of 2 different plasmas (orange and green lines) of netotic pixel signals aligned and normalized to their peak as classified by the pixROI method. Shaded regions are bounded by the Q1 and Q3 curves corresponding to the 25% and 75% quartiles. The curves are computed on signals gathering 1000 randomly sampled pixels for all videos corresponding to control neutrophils.

H. NET degradation profiles of control and TET2^{Mut} neutrophils in the absence of plasma. Median curves (solid lines) of netotic pixel signals aligned and normalized to their peak as classified by the pixROI method. Shaded regions are bounded by the Q1 and Q3 curves corresponding to the 25% and 75% quartiles. The half-life was 585 min (Q1 = 420 min; Q3 = 780 min) for control NETs and 720 min (Q1 = 495 min; Q3 = 975 min) for TET2^{Mut} NETs. The curves are computed on signals gathering 1000 randomly sampled pixels for all videos corresponding to the mouse type and experimental condition.

I. NET degradation profiles of control and TET2^{Mut} neutrophils when adding DNAse. Median curves (solid lines) of netotic pixel signals aligned and normalized to their peak as classified by the pixROI method. Shaded regions are bounded by the Q1 and Q3 curves corresponding to the 25% and 75% quartiles. The half-life of control NETs was 288 min (Q1 = 204 min; Q3 = 408 min) and the half-life of TET2^{Mut} NETs was 408 min (Q1 = 300 min; Q3 = 540 min). The curves are computed on signals gathering 1000 randomly sampled pixels for all videos corresponding to the mouse type and experimental condition.

Figure Supplementary 7

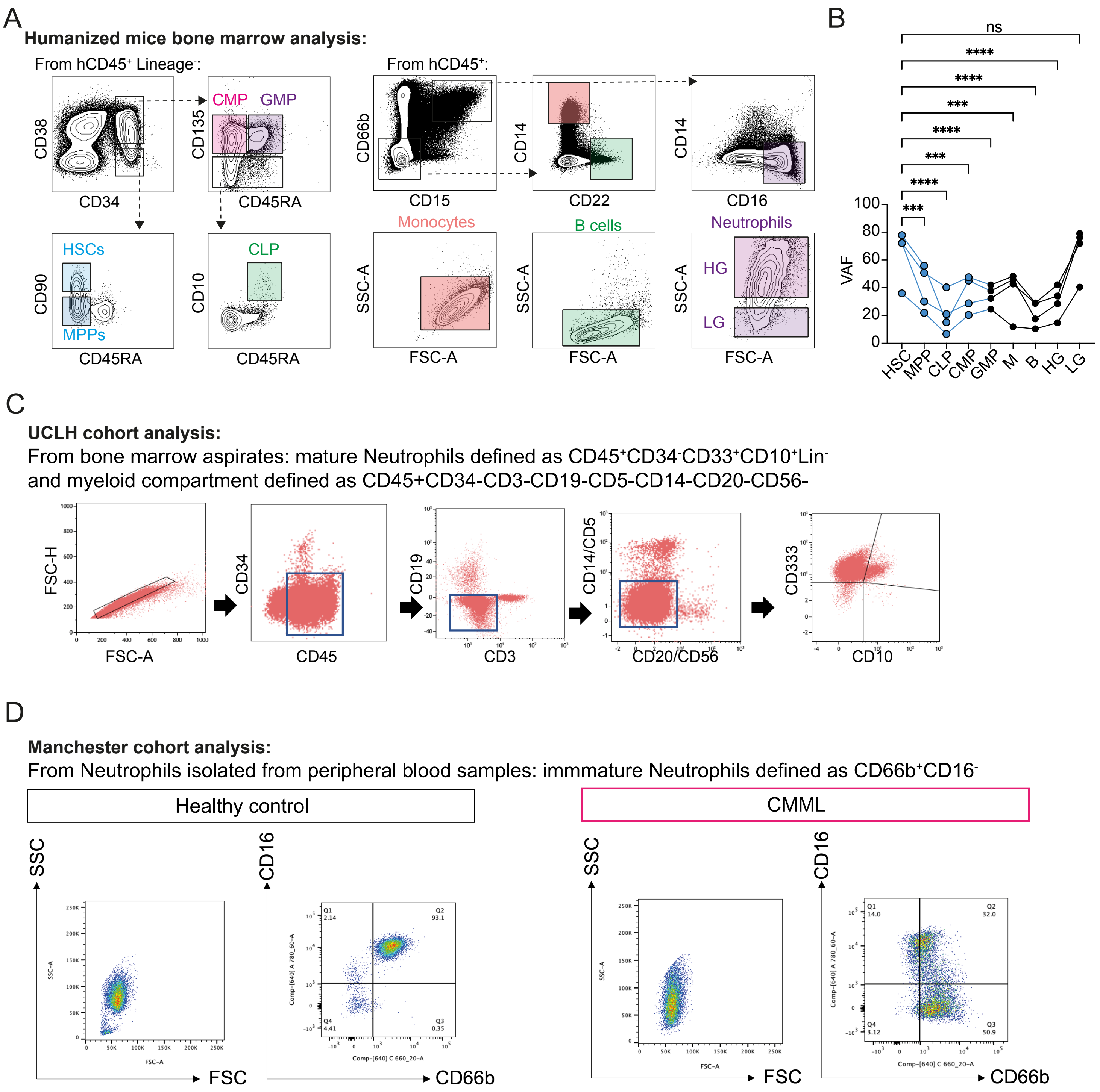

Figure S7. Isolation and identification of the different immune cells in TET2-derived human clonal hematopoiesis. Related to Figure 7.

A. Representative sorting strategy to analyze the TET2 VAF in different stem and progenitor cells and in differentiated hematopoietic cells.

B. Sequencing raw data from the different cell populations analyzed in the bone marrow to show that VAF of HSC is significantly higher compared to all populations except to LG neutrophils. Two-way ANOVA test, * $p<0.05$; ** $p<0.01$; *** $p<0.005$; **** $p<0.001$.

C. Representative flow gating strategy to analyze mature neutrophils defined as $CD45^{+}CD34^{-}CD3^{-}CD19^{-}CD5^{-}CD14^{-}CD20^{-}CD56^{-}CD33^{+}CD10^{+}$ in the bone marrow aspirate of the 22 UCLH cohort patients.

D. Representative flow gating strategy after the neutrophil isolation from peripheral blood samples of the 24 Manchester cohort patients.
